# A human-specific microRNA controls the timing of excitatory synaptogenesis

**DOI:** 10.1038/s41467-026-74753-y

**Published:** 2026-07-06

**Authors:** Michael Soutschek, Alessandra Lo Bianco, Simon Galkin, Tatjana Wüst, Koen Wentinck, David Colameo, Tomas Germade, Fridolin Gross, Lukas von Ziegler, Johannes Bohacek, Betina Elfving, Pierre-Luc Germain, Jochen Winterer, Tatjana Kleele, Gerhard Schratt

**Affiliations:** 1 Laboratory of Systems Neuroscience, Institute for Neuroscience, Department of Health Science and Technology, ETH Zurich, Zurich, Switzerland; 2 Department of Biology, Institute of Biochemistry, ETH Zurich, Zurich, Switzerland; 3 Laboratory of Molecular and Behavioural Neuroscience, Institute for Neuroscience, Department of Health Science and Technology, ETH Zurich, Zurich, Switzerland; 4 Translational Neuropsychiatry Unit A601, Department of Clinical Medicine, Aarhus University, Aarhus, Denmark; 5 Lab of Statistical Bioinformatics, IMLS, University of Zurich, Zurich, Switzerland; 6 Present Address: CNRS UMR5164 ImmunoConcEpT, University of Bordeaux, Bordeaux, France

**Keywords:** Molecular neuroscience, Cellular neuroscience

## Abstract

Neural circuit development in the human cortex is considerably prolonged in comparison to non-human primates, a trait that contributes to the remarkable cognitive capacity of modern humans. Here, we explore the regulatory role of non-coding RNAs, which dramatically expanded during brain evolution, in synapse development of human induced pluripotent stem-cell derived neurons. We found that inhibition of a human-specific microRNA, miR-1229-3p, alters the trajectory of human neuronal maturation and enhances excitatory synaptic transmission. Transcriptome analysis following miR-1229 knockdown revealed a downregulation of mitochondrial DNA (mtDNA) encoded genes. We further show that miR-1229 regulates mitochondrial morphology, mtDNA abundance as well as mitophagy, and that stimulation of mitochondrial metabolism rescues decreased calcium buffering in miR-1229-3p depleted neurons. Accordingly, miR-1229 directly targets an entire network of genes involved in mitochondrial function and ER-associated protein homeostasis. Our findings reveal an important function of human-specific miR-1229-3p in developmental timing of human synaptogenesis and generally implicate non-coding RNAs in the control of human connectivity and cognition.

## Abstract (web summary)

Here, the authors identify a human-specific microRNA, miR-1229-3p, which controls the timing of excitatory synapse development in human iPSC-derived glutamatergic neurons by tuning mitochondrial homeostasis.

## Introduction

The human brain enables remarkable cognitive achievements that are unparalleled in any other species. It contains more neurons and shows an increased size in comparison to the brains of other primates, particularly in the cerebellum and cortex^1^. However, this expansion represents an expected scaling across hominids^2^, which does not correlate with cognition^3^, suggesting that different mechanisms account for human-specific cognitive abilities. In this regard, human neurons lag in development when compared to the developmental timing of other species (a concept called human neoteny), illustrated by a slower reach of the maximal synapse density followed by a delayed pruning of the acquired synapses^1^. The resulting prolonged synaptogenesis and synaptic plasticity period has been suggested to be key in the enhanced social and cultural learning of humans^4^.

Little is known regarding the genetic mechanisms underlying human-specific aspects of synaptogenesis and plasticity. For example, human-specific non-synonymous base pair substitutions in the *Foxp2* gene, which is mutated in a monogenic speech disorder, lead to increased neurite outgrowth and synaptic plasticity of corticostriatal circuits along with behavioral phenotypes in mice^5^. Moreover, expression of the human-specific *Srgap2c* in mice, which originates from a gene duplication, delays synapse maturation in cortical pyramidal neurons^1,6,7^. However, due to the generally high conservation of protein-coding genes, it has been suggested early on that most phenotypic differences between humans and other primates might be rather caused by changes in non-coding DNA regulating gene expression^8^. Regulatory regions of the genome that evolved particularly fast in humans, such as the human accelerated regions (HAR) and human gained enhancers (HGE), represent interesting candidates in this regard^9,10^. In addition to fulfilling gene regulatory functions *in cis*, non-coding DNA is pervasively transcribed into a plethora of non-coding RNA (ncRNA) families that control gene expression in trans, such as long non-coding RNAs (lncRNAs) and microRNAs (miRNAs)^11^. An expansion in the repertoire of miRNAs was suggested to contribute to the increased complexity of the brain throughout evolution^12,13^. Human miRNA expression in the brain has been profiled previously in adulthood and development from post-mortem tissue samples^14–17^. Moreover, the human-specific miR-941 has been investigated with regard to its expression levels in the brain and putative functionality in cell lines^18^. However, despite their well-documented role in neuronal development in vertebrates^19^, it is unknown whether miRNAs control aspects of human neoteny, such as delayed neuronal maturation and synaptogenesis.

Here, we identify the human-specific microRNA miR-1229-3p to regulate synapse development in human neurons employing a protocol to culture functional human neurons without the addition of animal glial cells. We perform an in-depth molecular profiling from these neurons and integrate this data with a morphological characterization, revealing miR-1229-3p to closely follow the dynamics of synapse development in its expression pattern. Depletion of miR-1229-3p leads to an increase in synaptic density at early developmental time points, followed by a decrease in dendritic complexity later during differentiation. These cellular phenotypes are accompanied by a specific regulation of mitochondrial DNA (mtDNA) - encoded RNAs, a reduction in mitochondrial length and increased lactate production, together indicating reduced metabolic activity of mitochondria in neurons lacking miR-1229-3p. Overexpression of miR-1229-3p implies a compendium of miRNA targets associated with mitochondrial ER contact sites. Mechanistically, we therefore propose a model in which depletion of miR-1229-3p increases mitochondrial-ER interactions, initially promoting synaptic development before causing detrimental effects later during development.

## Results

### A glial-free neuronal cell culture model to study human excitatory synaptogenesis

**Fig. 1:**
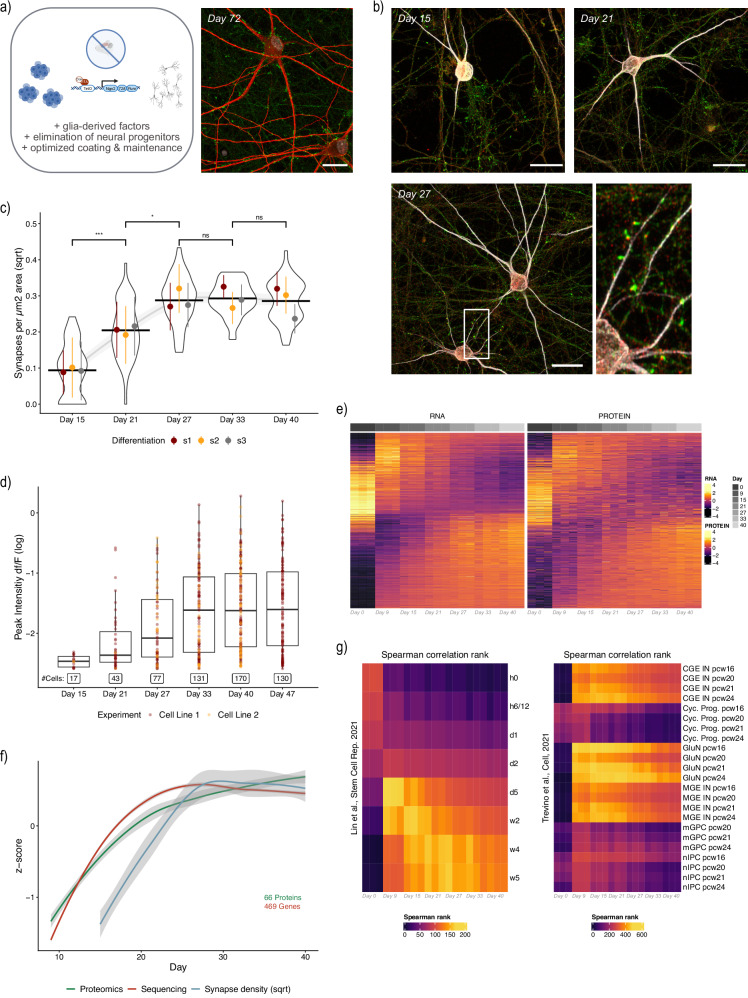
A glial-free protocol to study human excitatory synapse development. **a** Overview of our protocol to differentiate human neurons without the requirement to add glial cells, together with an example image of day 70 igNeurons stained with MAP2 (red), SYN1 (green) and Hoechst (grays). The scale bar indicates 15 µm. **b** Example images of igNeurons used for synapse quantification stained with MAP2 (grays), SYN1 (green) and PSD95 (red). Scale bars indicate 15 µm. **c** Synapse co-cluster quantification over five time points. Shown are violin plots of all data points together with the mean and standard deviation of each differentiation. Displayed are only statistical comparisons between neighboring time points. (* = *p*-value < 0.05, *** = *p*-value < 0.001). Further information on statistical analyses, the number of biological replicates and exact *p*-values are provided in the Supplementary Data S1. **d** Quantification of the normalized Ca-imaging amplitude (df/F) over the time course of neuronal differentiation. Measured were two different cell lines. Shown are boxplots including the median, hinges at the 25^th^ & 75^th^ percentiles and whiskers extending to a max. of 1.5x inter-quartile range. **e** Heatmap indicating scaled expression values of mRNAs and corresponding proteins. Each line depicts one gene, each column one replicate. Plotted are the top 1000 genes significantly changing during the neuronal time points (FDR < 0.01) and the corresponding proteins. In total, 11’799 genes (FDR < 0.01 & logFC > 2) and 5’280 proteins (FDR < 0.01) changed significantly over the full time course. The igNeurons time course data can be further accessed at https://ethz-ins.org/igNeuronsTimeCourse. **f** Smoothed means of scaled RNA expression levels of synapse-associated genes (Cluster 9, see Supplementary. Fig. S6f, g), the corresponding protein expression levels and the results of the synaptic co-cluster analysis as presented in (**c**). See Supplementary Data S2 for the individual genes used for this plot. **g** Heatmaps showing ranked Spearman correlations of our igNeuron time course RNA sequencing dataset to pseudo-bulk values of single-cell RNA sequencing datasets of induced NGN2 neurons cultured together with astrocytes (left, data from Lin et al.^21^) and the developing human cortex (right, data from Trevino et al.^22^). Figure panel (**a**) was created with BioRender. Wüst, T. (2025) https://BioRender.com/8d5ckza.

To systematically profile human miRNA expression over the course of synaptogenesis, we established glial-free 2D cultures of human induced pluripotent stem cell (hiPSC) derived excitatory cortical neurons by modifying a previously published NGN2-overexpression protocol^20^ (Fig. 1a and Supplementary Fig. S1a–c; materials and methods). miRNAs are highly conserved between different cell types and across species. The omission of glial cells was therefore a prerequisite for faithful detection of miRNAs exclusively in excitatory cortical neurons. Based on immunostaining for excitatory synaptic marker proteins PSD95 and SYN1, neurons cultured with our protocol (hereafter referred to as induced glial-free neurons, or igNeurons for short) begin to form structural synapses (synaptic co-clusters) at 15 days of differentiation (Fig. 1b). We further observed spine-like structures, a hallmark of excitatory synapse maturation, from day 27 on (Supplementary Fig. S1d). When quantifying three independent igNeurons differentiations, we observed that PSD95-Synapsin co-cluster density steadily increases until day 27, before reaching a plateau (Fig. 1c and Supplementary Fig. S2). On the other hand, dendritic complexity expands over the entire time course, with the steepest rise after day 27 (Supplementary Fig. S3). Calcium (Ca-) imaging upon transduction with the calcium indicator GCaMP6f shows spontaneous activity of igNeurons from day 21 on (Fig. 1d and Supplementary Fig. S4a, b). Activity can be blocked by the AMPAR antagonist NBQX, suggesting that calcium signals are generated by synaptic activity (Supplementary Fig. S4c, d). Patch-clamp recordings further showed that igNeurons could generate multiple action potentials upon depolarizing current injections, confirming their ability to maintain repetitive firing (Supplementary Fig. S4e). Taken together, human igNeurons form functional excitatory synapses within 3-4 weeks, which is comparable to previous protocols using NGN2-induced neurons in the presence of mouse astrocytes^20^. Molecular characterization of igNeurons using ribosomal depletion RNA-seq and label-free proteomics (Supplementary Fig. S5–S7) confirmed efficient neuronal maturation, and the absence of glial cells, as key pluripotency and astrocytic markers are either not expressed or decline during neuronal time points (Supplementary Fig. 6c, d). We further observe widespread gene expression changes until day 27, consistent with prevailing morphological changes, such as the formation of excitatory synapses, in this time window (Fig. 1e). For example, expression of genes encoding for excitatory synaptic proteins (SYN1, GRIA2/4, GRIN2A/B) follows the pattern of our synaptic co-cluster and calcium imaging quantification (Supplementary Fig. 6e). Plotting the scaled expression of synapse-associated genes (Supplementary Fig. S6f, g), together with the corresponding proteins and the values obtained from the synapse co-cluster analysis (Fig. 1c), shows that all three measurements follow a similar pattern (Fig. 1f), whereby RNA expression precedes the morphological development by roughly one time point or six days (Fig. 1g). We further compared RNA expression in our human igNeurons to two published single cell RNA-seq datasets. Neurons differentiated with NGN2 in the presence of astrocytes^21^ displayed a similar maturation trajectory compared to igNeurons (Fig. 1g, left panel). Furthermore, expression profiles of glutamatergic and upper-layer neurons at developmental stages pcw 16 – pcw 24 obtained from human post-mortem tissue^22,23^ showed a strong correlation with those from igNeurons at days 9-40 (Fig. 1g and Supplementary Fig. S8). Thus, developmental gene expression profiles in igNeurons should be useful to predict potential functional regulators of excitatory synaptogenesis.

### The ncRNAome during human excitatory synaptogenesis

**Fig. 2:**
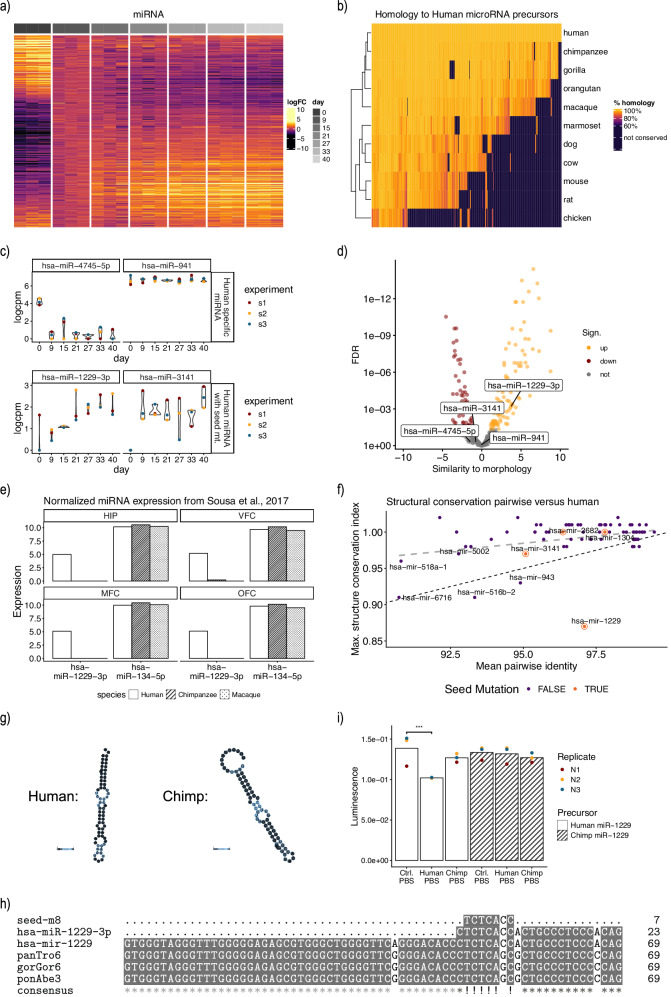
The ncRNAome during human excitatory synapse development. **a** Heatmap of significantly changing miRNAs over the time course of excitatory synapse development (FDR < 0.05, logFC normalized to day 9). **b** Heatmap showing the percent homology of expressed human miRNA precursors with orthologs identified by reciprocal blast in selected species. Sequences with less than 60% homology were considered non-homologous. **c** Expression examples for human-specific miRNAs not found in other genomes (upper row) and miRNAs with a human-specific seed mutation (lower row) during human neuronal differentiation. **d** Volcano plot showing the significance and strength of a regression of each miRNA’s expression on excitatory synaptic co-cluster formation across days. **e** Normalized expression of miR-134-5p and miR-1229-3p in four selected brain areas (data obtained by Sousa et al.^14^, the full overview of all profiled brain regions can be found in Supplementary Fig. S1^3^). HIP = hippocampus, VFC = ventrolateral prefrontal cortex, MFC = medial prefrontal cortex, OFC = orbital prefrontal cortex. **f** Pairwise structural conservation analysis of expressed human miRNA precursors with at least one mismatch identified in analyzed species across primates (excluding marmoset). Plotted is the mean identity of the contrasted precursor sequences on the *x*-axis and the structural conservation index generated by RNAz on the *y*-axis (following an idea of McCreight et al.^87^). The gray dashed line indicates the linear regression of all analyzed precursors. The black reference line (slope = 0.01 and intercept = 0) roughly marks a threshold below which miRNA precursors can be regarded as not structurally conserved. **g** RNAfold secondary structure prediction of human pre-miR-1229 and the putative ortholog found in chimpanzees. **h** Sequence alignments of hsa-miR-1229-3p, its seed sequence and precursor as well as orthologs among hominids (panTro6 = chimpanzee, panGor6 = gorilla, panAbe3 = orangutan). **i** Luciferase measurements of miR-1229 overexpression constructs transfected together with miRNA perfect binding site reporters in HEK cells (*** = *p*-value < 0.001). Further information on statistical analyses, the number of biological replicates and exact *p*-values are provided in the Supplementary Data S1.

Of note, mRNA and protein expression trajectories are uncorrelated for a group of genes (Supplementary Fig. S7g, h), suggesting the involvement of post-transcriptional regulatory molecules, such as ncRNAs, in human excitatory synaptogenesis. In agreement with this, we observed extensive developmental gene expression changes of regulatory long non-coding RNAs, such as lncRNAs and circRNAs (Supplementary Fig. S9, S10), in our Ribo-minus RNA-seq dataset. However, for this initial study, we decided to focus on miRNAs due to their well-established role in synapse development in other vertebrates. Therefore, we performed small RNA sequencing on the same differentiations that were processed for previous analyses (Supplementary Fig. S11, cf. Figure 1). We identified 338 significantly changing miRNAs over the entire time course, of which 182 were significantly changing when considering only neuronal days (day 9 – day 40) (FDR < 0.05) (Fig. 2a). In addition, we also quantified snoRNAs (with 81 significantly changing, FDR < 0.05) and piRNAs (122 significantly changing, FDR < 0.05) (Supplementary Fig. S11c). Clustering the miRNA expression dataset according to similar expression trajectories rendered a total of 5 independent clusters (Supplementary Fig. S12a). Many miRNAs which are known to be involved in neuronal morphogenesis based on rodent studies (e.g., miR-181, miR-124, miR-134) are found in cluster 2 (“begin up”; Supplementary Fig. S12b), which features miRNAs that are rapidly induced after the stem cell - neuron transition (day 9 onwards). In contrast, miRNAs known to be involved in synaptic plasticity (e.g., miR-129)^19^ are present in cluster 5 (“late up”; Supplementary Fig. S12c), which is characterized by a later induction (day 27 onwards).

We next attempted to characterize human-specific features of miRNA regulation in more detail. Towards this aim, we first correlated our miRNA expression data to a small RNA sequencing dataset obtained from induced mouse neurons (see methods). We found that out of the 233 miRNAs commonly detected in both datasets, 180 change significantly during human neuronal differentiation (FDR < 0.05, all time points). Expression patterns of these 180 miRNAs were mostly correlated between human and mouse (Supplementary Fig. S12d), except for 14 miRNAs, which showed strongly anti-correlated expression dynamics, as exemplified by miR-708 (Supplementary Fig. S12e, f). We further searched for miRNAs whose sequences are not conserved in other species, including non-human primates. Employing the conservative approach of searching for orthologs in other species by reciprocal blasting of all listed human miRNA precursors of miRbase version 22 (see methods), we found that only a small fraction of miRNAs can be safely classified as human-specific (25 miRNAs with no more than 60% sequence conservation in any of the tested species, Supplementary Data S3–S7) in agreement with previous publications^18,24^. In addition, we discovered 52 miRNAs with human-specific seed changes, which are expected to have unique target genes in humans. Among this conservative estimate of a total of 77 “human-specific” miRNAs, only four (the newly evolved miR-4745-5p and miR-941 as well as the seed mutants miR-1229-3p and miR-3141) were expressed in our dataset (Fig. 2b, c).

While miR-941 is consistently expressed at high levels, miR-4745-5p and miR-3141 change mostly from stem cell to neuron conversion and stay rather constant during neuronal maturation. miR-1229-3p represents the only human-specific miRNA with a dynamic expression pattern during the neuronal days of differentiation, suggesting a role in human synaptogenesis. This finding was further corroborated when we performed gene-wise linear regression of miRNA expression profiles with the neurodevelopmental trajectory inferred by synapse density quantification (cf. Figure 1c) from the same neuronal differentiations (Fig. 2d). In contrast to the other three human-specific miRNAs, miR-1229-3p expression closely follows synapse density development, as do known regulators of synaptogenesis such as miR-181c-5p^25,26^. To obtain further information regarding the in vivo expression of miR-1229-3p, we screened existing literature and found that miR-1229-3p is consistently expressed across various regions of the human brain while being basically absent from chimpanzee and macaque postmortem samples (Fig. 2e and Supplementary Fig. S13). miR-1229-3p was further robustly expressed in post-mortem brain sections, fixed in paraformaldehyde (PFA), from a Danish cohort of psychiatric patients (Supplementary Fig. 14). These data demonstrate that miR-1229-3p is not only expressed in our in vitro igNeuron model of human synaptogenesis, but also in human brain regions relevant for cognition.

Based on a structural conservation analysis of all human miRNA precursors with mismatches in orthologs found in primates (excluding marmoset), miR-1229-3p shows the lowest structural conservation index, despite a high sequence similarity (Fig. 2f and Supplementary Fig. S15a). This lack of structural conservation is further illustrated when plotting the predicted secondary structures of human and chimp pre-miR-1229 (Fig. 2g). Since the predicted chimp pre-miR-1229 is lacking a 2-nucleotide overhang at the 3’end of the precursor required for efficient Dicer-mediated processing^27^, no production of mature miR-1229-3p is expected in chimpanzee. In addition, alignment of hsa-pre-miR-1229 with blasted sequences of higher apes shows the presence of four human-specific point mutations, one of which is located in the seed region (Fig. 2h). In agreement with the non-functionality of a putative chimp-miR-1229, expression of the chimp miR-1229 mirtron, in contrast to its human counterpart, was unable to downregulate a perfect miR-1229-3p binding site reporter in a luciferase assay (Fig. 2i and Supplementary Fig. S15b). Finally, to assess the conservation of predicted miR-1229-3p targets, we used scanMiR to inspect 3’UTR sequences that we extracted from the chimp genome by lifting over human coordinates from expressed 3’UTRs for potential miR-1229-3p binding sites (see methods). We noticed that almost all binding sites are very well conserved between human and chimpanzee (in contrast to predicted binding sites on mouse 3’UTRs), even regarding their predicted strength (Supplementary Fig. S15c, d). This pattern holds similarly true for all expressed miRNAs (Supplementary Fig. S15e), suggesting that evolutionary adaptation of miRNA regulation primarily occurred on the miRNA rather than on the target side. Taken together, four point-mutations within the miR-1229 gene enabled efficient Dicer-mediated processing and the recognition of evolutionary conserved target genes in human neurons.

### Human-specific miR-1229-3p regulates the timing of excitatory synaptogenesis

**Fig. 3:**
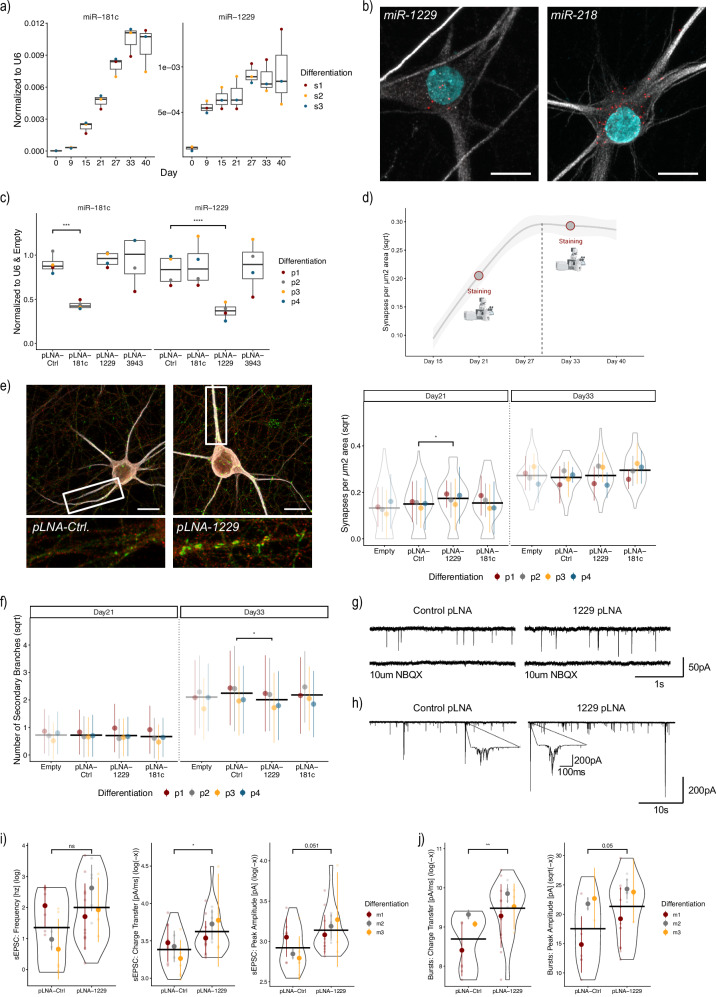
Human-specific miR-1229-3p restricts excitatory synapse development. **a** TaqMan miRNA qPCRs for miR-1229-3p and miR-181c-5p on the time course samples used for the small RNA sequencing (Fig. 2e, g). Shown are boxplots including the median, hinges at the 25^th^ & 75^th^ percentiles and whiskers extending to a max. of 1.5x inter-quartile range. **b** SmFISH signal (red) showing cytoplasmic localization of miR-1229-3p and miR-218-5p in igNeurons (day 40). MAP2 is displayed in gray and Hoechst in cyan (scale bars = 10 µm). **c** TaqMan qPCR for indicated miRNAs using RNA from igNeurons (day 21) following pLNA application at day 9. The primate-specific miR-3943 served as a specificity control. Displayed expression levels are normalized to U6 and the “Empty” condition. Shown are boxplots including the median, hinges at the 25^th^ & 75^th^ percentiles and whiskers extending to a max. of 1.5x inter-quartile range. **d** Smoothed mean and 95% level of confidence band of synapse densitites (Fig. 1c) with circles indicating the two time points (day 21 & day 33) at which morphology was assessed upon pLNA addition. **e** Representative images of pLNA-treated igNeurons (MAP2 in gray, PSD95 in red and SYN1 in green) are displayed on the left (scale bars = 10 µm). Quantification of synaptic co-clusters (PSD-95/SYN1) in pLNA-treated igNeurons at days 21 and 33 is shown on the right. Displayed are violin plots of all data points (sqrt) together with the mean and standard deviation of each differentiation. **f** Quantification of the number of secondary dendrite branches in pLNA treated igNeurons at day 21 and day 33. Shown are the mean and standard deviation of all data points (sqrt) of each differentiation. **g** Spontaneous excitatory postsynaptic current (sEPSC) traces of pLNA-treated igNeurons with or without NBQX. **h** Example traces of sEPSC bursts recorded by patch-clamp. **i**, **j** Quantification of sEPSC frequency, charge transfer and amplitude (recorded from 15 (pLNA-Ctrl) and 21 (pLNA-1229) neurons of three differentiations) (**i**), as well as quantification of charge transfer and amplitude in recorded sEPSC bursts (obtained from 11 (pLNA-Ctrl) and 21 (pLNA-1229) neurons of three differentiations) (**j**). Shown are individual datapoints, the resulting violin plots, as well as the mean and standard deviation of each differentiation. (* = *p*-value < 0.05, ** = *p*-value < 0.01, *** = *p*-value < 0.001, **** = *p*-value < 0.0001). Further information on statistical analyses, the number of biological replicates and exact *p*-values are provided in the Supplementary Data S1. Panel (**d**) was created using BioRender. Wüst, T. (2025) https://BioRender.com/b7ji9d4.

Using miRNA qPCR and single-molecule fluorescent in-situ hybridization (FISH), we confirmed that miR-1229-3p is dynamically expressed over the course of human neuron differentiation, in a similar way as miR-181c, a known regulator of neuronal morphogenesis in rodents (Fig. 3a, b)^25^. Together with the unique evolutionary features described above, this led us to consider the possibility that miR-1229-3p might be involved in the regulation of excitatory synapse development of human neurons. To interfere with miR-1229-3p expression, we delivered locked nucleic acid (LNA) modified antisense oligonucleotides directed against miR-1229-3p (pLNA-1229) to igNeurons via bath application in the culture media. In addition, we included pLNAs directed against miR-181c-5p (pLNA-181) and a corresponding scrambled control sequence (pLNA-Ctrl) in our experiments. Using a fluorescently labeled pLNA-Ctrl, we confirmed that pLNAs are taken up at nearly 100% efficiency, remain stable inside the cells and don’t cause detrimental effects on the neuron morphology even at high concentrations (Supplementary Fig. S16). miRNA qPCR further confirmed that the pLNAs specifically decrease the expression of the cognate miRNA (Fig. 3c).

To test the functional role of miR-1229-3p over the course of excitatory synapse development, we applied pLNAs from day 9 on and monitored synaptic co-cluster density and dendritogenesis. We focused on two time points, one during the linear synaptic growth phase (day 21) and one during the plateau phase (day 33) (Fig. 3d).

Remarkably, synapse co-cluster density analysis revealed a significant increase of synaptic co-clusters at day 21 upon depletion of miR-1229-3p compared to the pLNA-Ctrl and pLNA-181 conditions. This effect was no longer seen at day 33 (Fig. 3e and Supplementary Fig. S17). Inhibition of miR-181c-5p showed a trend towards more synapses at the plateau phase (day 33), which however, did not reach statistical significance. Surprisingly, quantification of dendritogenesis showed a decrease of dendritic complexity upon depletion of miR-1229-3p specifically at the later time point (Fig. 3f and Supplementary Fig. S18). Together, these results suggest that miR-1229-3p initially functions as a repressor of excitatory synapse formation but is required for dendritic branching during later stages of human neuron development.

To characterize the functional properties of igNeurons depleted from miR-1229-3p, we performed patch-clamp recordings of spontaneous excitatory postsynaptic currents (sEPSC) during the plateau phase when synapses had sufficiently matured (day 40–42) (Fig. 3g, h). At this time point, igNeurons displayed spontaneous activity – a proxy for functional synapses and cell-cell communication – at a similar frequency as human NGN2-neurons cultured together with astrocytes for 30-35 days^28^, further confirming the suitability of our protocol to investigate human excitatory synapse development. Interestingly, sEPSC quantification revealed a significantly higher charge transfer in miR-1229-3p depleted neurons in comparison to the control condition (Fig. 3i). Neurons treated with the pLNA-1229 showed in addition, a trend towards a higher sEPSC amplitude (*p*= 0.051), while the sEPSC frequency as well as rise and decay time were not changed (Fig. 3i and Supplementary Fig. S19a–c). Besides sEPSC-events, we also detected occasional large-amplitude bursts (Fig. 3h, inset). Focusing on these bursts, we again observed a significant increase in the total charge transfer upon depletion of miR-1229-3p, as well as a trend towards an increased burst peak amplitude (*p* = 0.05) (Fig. 3j). The burst duration remained unchanged (Supplementary Fig. S19d). Together, these results demonstrate that the human-specific miR-1229-3p controls the developmental timing of excitatory synaptogenesis and impacts excitatory synaptic function.

### miR-1229-3p depletion impairs the expression of mitochondrial genes

**Fig. 4:**
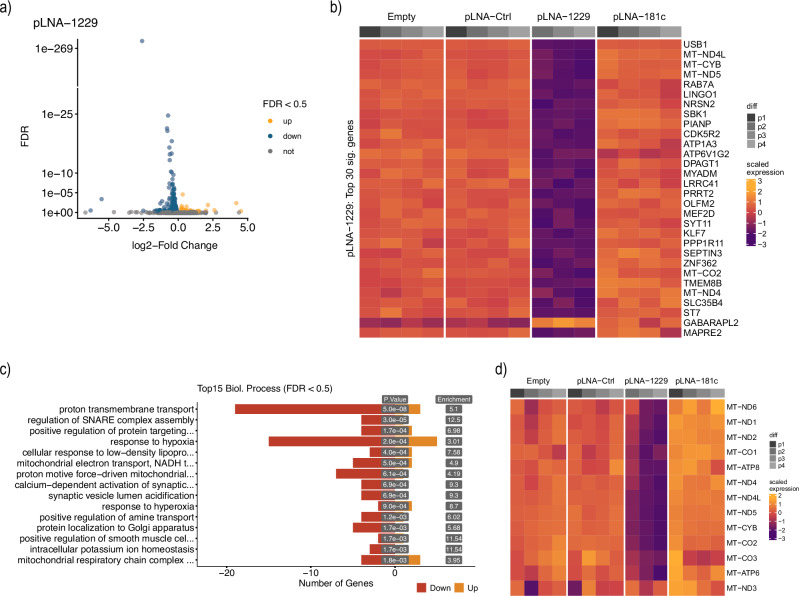
miR-1229-3p regulates genes involved in mitochondrial homeostasis. **a** Volcano plot indicating significantly changing genes upon miR-1229-3p depletion. Upregulated genes (FDR < 0.5) are displayed in yellow, downregulated genes (FDR < 0.5) in blue. **b** Heatmap (scaled gene expression) indicating the 30 most significantly changing genes upon pLNA-1229 treatment. **c** GO-Term analysis ("Biological Processes") of significantly changing genes (FDR < 0.5) in the pLNA-1229 condition. Displayed are the top 15 significant GO-Terms with less than 500 genes annotated. The number of significantly changing genes within each GO-term is represented by the red (downregulated) and yellow (upregulated) bars. **d** Heatmap showing the scaled expression of genes encoded by the mitochondrial genome in pLNA-treated igNeurons.

The observation that miR-1229 depletion leads to significant morphological and functional changes during human excitatory synapse development prompted us to further investigate the molecular mechanisms underlying these effects. We therefore profiled the protein-coding transcriptome of pLNA-1229, pLNA-181c, pLNA-Ctrl treated, as well as untreated igNeurons by polyA-RNA sequencing during the synaptic growth period at day 21 (cf. Fig. 3d and Supplementary Fig. S20, S21). When comparing the transcriptome of pLNA-1229 to pLNA-Ctrl treated and untreated neurons (see methods), we detected 414 candidate differentially expressed genes (cDEG) (FDR < 0.5; Fig. 4a). Strikingly, the vast majority of cDEGs were downregulated upon miR-1229-3p inhibition, suggesting that most of the observed changes are not a primary effect due to the loss of miRNA repression, but rather secondary gene expression changes (Fig. 4a, b). To gain further insight into the cellular pathways regulated by miR-1229-3p, we performed gene ontology (GO-term) analysis of cDEGs (Fig. 4c and Supplementary Fig. S20c). Among the top 15 biological processes enriched in the dataset, several were associated with mitochondrial function. While most mitochondrial proteins are transcribed from the nuclear genome, mitochondria still retain their own DNA (mtDNA), encoding for 13 proteins of the electron transport chain. Strikingly, plotting all protein-coding genes transcribed from mtDNA showed a specific downregulation of almost all of them specifically in the pLNA-1229 condition (Fig. 4d), while various genes related to mitochondrial metabolism and surveillance are upregulated upon miR-1229-3p knockdown (Supplementary Fig. S22a). Since neither the expression of nuclear-encoded mitochondrial complex I genes (Supplementary Fig. S22b) nor predicted nuclear targets of the mitochondrial transcription factor TFAM were altered (Supplementary Fig. S22c), we conclude that miR-1229-3p inhibition specifically affects mitochondrial gene expression. This effect is not due to increased mutation frequency in the mitochondrial genome, since the number of SNPs in mitochondrial transcripts is not altered by miR-1229-3p inhibition (Supplementary Fig. S22d, e). Moreover, we see that the expression levels of mtDNA encoded genes as well as the abundance of mitochondrial ribosomal proteins are highly dynamic throughout neuronal development (Supplementary Fig.  S23a, b). Considering these observations and the apparent importance of mitochondrial metabolism for the evolution of the human brain^29,30^, we decided to study a potential regulation of mitochondrial function by miR-1229-3p in further detail.

### miR-1229-3p controls mitochondrial function during human excitatory synaptogenesis

**Fig. 5:**
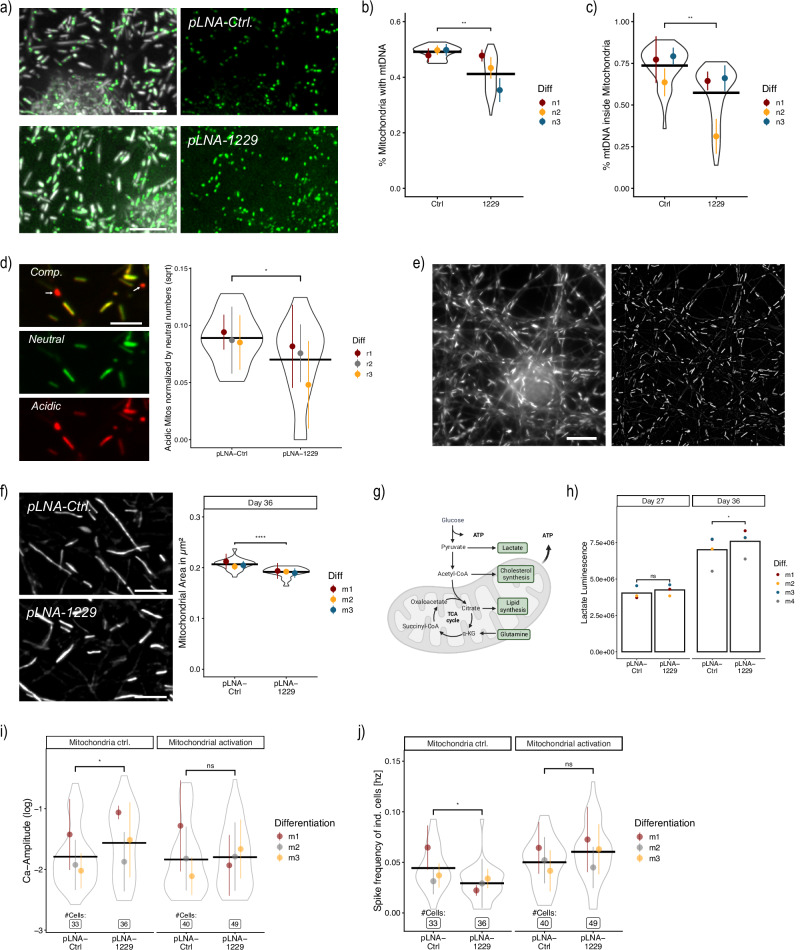
miR-1229-3p controls mitochondrial function during human excitatory synapse development. **a** iSIM images of mitochondria (PKMO, gray) and mtDNA (SYBR Gold, green) in pLNA-treated iND3 neurons at day 25 (scale bars = 5 µm). **b**, **c** Percentage of mitochondria containing mtDNA per image (**b**) and the percentage of mtDNA puncta inside mitochondria (compared to the total number of detected mtDNA puncta per image) (**c**) in iND3 neurons at day 25. Shown are violin plots of all data points together with the mean and standard deviation of each differentiation. **d** Example image zoom-in (left) and quantification (right) of igNeurons (day 23) that were transduced with a mtKeima virus to determine mitophagy. Acidic mitochondria that undergo mitophagy are highlighted with the white arrow (scale bar = 3 µm). Shown are violin plots of all data points together with the mean and standard deviation of each differentiation. **e** Non-processed (left) and processed (right) SIM image of mitochondria (PKMO) in igNeurons (day 29). The scale bar indicates 10 µm. **f** Zoom-in (left) and quantification (right) of mitochondrial area in igNeurons (day 36) treated with pLNA (scale bars = 3 µm). Shown are violin plots of all data points together with the mean and standard deviation of each differentiation. **g** Scheme illustrating neuronal energy supply by mitochondrial respiration and glycolysis. **h** Quantification of secreted lactate levels in media of pLNA-treated igNeurons at indicated days. **i**, **j** Calcium peak intensity (df / F) (**i**) and frequency (**j**) of human neurons recorded at day 37. Mitochondria were activated by adding GSK-2837808A and AlbuMAX to the media. Shown are violin plots of all data points together with the mean and standard deviation of each differentiation. (* = *p*-value < 0.05, ** = *p*-value < 0.01, **** = *p*-value < 0.0001). Further information on statistical analyses, the number of biological replicates and exact *p*-values are provided in the Supplementary Data S1. Panel (**g**) was created in BioRender. Wüst, T. (2026) https://BioRender.com/eg3htmo.

Since we observed a significant downregulation of mitochondrial gene expression in pLNA-1229 treated neurons (Fig. 4d), we monitored mitochondrial (mt) DNA abundance and localization in igNeurons using SYBR Gold (Fig. 5a). Consistent with our RNA-Seq results, this analysis revealed a significant decrease in the percentage of mitochondria with mtDNA in pLNA-1229 treated neurons as well as an increase in the fraction of mtDNA puncta localized outside of mitochondria (Fig. 5b, c). mtDNA release is a hallmark of mitochondrial stress and often observed if damaged mitochondria fail to activate proper quality control mechanisms, such as mitophagy^31^. Indeed, transducing igNeurons with a pH-sensitive lentiviral mito-Keima (mtKeima) construct^32,33^, revealed a significant decrease of acidic mitochondria in the processes of pLNA-1229 treated neurons (Fig. 5d), indicative of reduced mitophagy. Since mitochondrial fragmentation contributes to the removal of deleterious mtDNA^34^, we performed a detailed analysis of mitochondria ultrastructure using live-cell super-resolution imaging during human neuronal maturation. Consistent with enhanced mitochondrial fragmentation upon miR-1229-3p inhibition, mitochondrial organelle size (area and length) was significantly reduced in pLNA-1229 compared to pLNA-Ctrl treated igNeurons at day 25 and day 36 (Fig. 5e, f and Supplementary Fig. S24).

Despite these indications of mitochondrial stress, mitochondria in pLNA-1229 treated neurons seem to be overall functional with an intact mitochondrial membrane potential at day 23 (Supplementary Fig. 25). Moreover, we didn’t observe an increase in the expression of interferon receptors or genes involved in the cGAS/Sting pathway (Supplementary Fig. 26), as it has been described upon mtDNA release in other systems^31^. Given that the abundance of mitochondrial respiration-associated proteins increases constitutively during human neuronal maturation (Supplementary Fig. S7e, f), we hypothesized that the reductions of mtDNA content and related gene expression changes in pLNA-1229 treated neurons might reveal an impairment of oxidative phosphorylation at later stages of maturation. This, in turn, would shift the provision of energy supply towards glycolysis, reflected by enhanced lactate production (Fig. 5g). In line with a decreased mitochondrial metabolic activity upon sustained miR-1229-3p depletion, we observed a significantly increased lactate concentration in the media collected from pLNA-1229 treated neurons in comparison to control neurons following prolonged inhibition of miR-1229 at day 36 (Fig. 5h).

Besides energy provision, neuronal mitochondria play an important role in calcium buffering and uptake upon stimulation, particularly at presynaptic termini^35^. Accordingly, mitochondrial dysfunction has been associated with reduced mitochondrial calcium buffering, ultimately leading to elevated calcium levels in the cytoplasm and at synapses^36–38^. We therefore performed cytoplasmic Ca-imaging at day 37 of the neuronal differentiation using the calcium indicator GCaMP6f. Consistent with increased glycolytic activity upon sustained miR-1229-3p inhibition, we observed an increase of the calcium signal amplitude in miR-1229-3p depleted neurons in comparison to the control (Fig. 5i). The spike frequency was decreased in miR-1229-3p depleted neurons (Fig. 5j), while the peak duration was not changed (Supplementary Fig. S27). These results, particularly the increased spike amplitude and unchanged peak duration, corroborate the electrophysiological recordings of spontaneous bursts in igNeurons treated with pLNA-1229 (cf. Figure 3j and Supplementary Fig. S16d). Strikingly, activation of mitochondrial respiration by the addition of GSK-2837808A and AlbuMAX^30^ rescued the elevated calcium signal amplitude as well as the reduced frequency (Fig. 5i, j), demonstrating that impaired calcium buffering in miR-1229-3p depleted human neurons is a consequence of disturbed mitochondrial TCA cycle activity.

Taken together, several lines of evidence indicate that inhibition of miR-1229-3p leads to an impairment of mitochondria over the course of human neuronal differentiation, ultimately causing defects in calcium buffering and metabolic function.

### miR-1229-3p targets a network of genes involved in mitochondrial – ER homeostasis

**Fig. 6:**
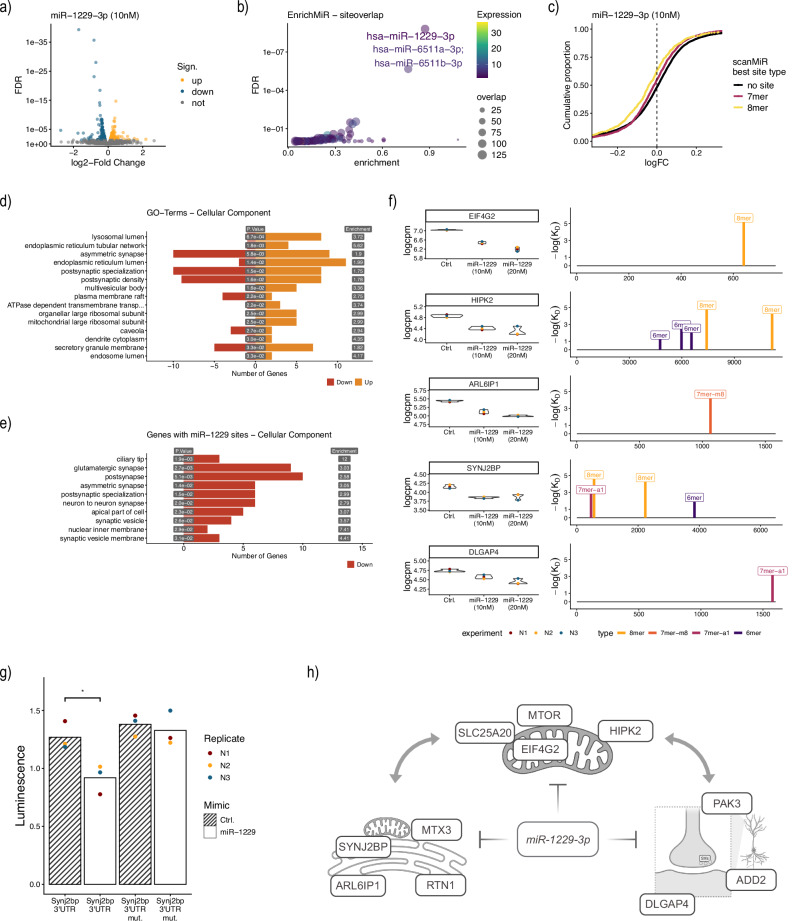
miR-1229-3p targets a network of genes involved in mitochondrial – ER homeostasis. **a** Volcano plot indicating gene expression changes (yellow: sign. upregulated, blue: sign. downregulated) upon overexpression of a miR-1229-3p mimic (10 nM) in SH-Sy5y neuroblastoma cells (FDR < 0.05). **b** miRNA binding motif enrichment analysis with enrichMiR^39^ on significantly downregulated genes from a) using the siteoverlap test on a collection of scanMiR predicted miRNA binding sites. Displayed is the FDR value on the *y*-axis and the log2-fold enrichment on the *x*-axis. See Supplementary Fig. S28c for further enrichMiR analyses. **c** Cumulative distribution plot (CD-Plot) demonstrating the downregulation of scanMiR predicted miR-1229-3p targets dependent on the site type. **d** GO-Term enrichment analysis on differentially expressed genes of the 10 nM DEA (FDR < 0.05). Displayed are the 15 most significant “Cellular Component” GO-Terms with > 15 & < 500 annotated genes. Bars indicate the number of significantly up- (yellow) or downregulated (red) genes associated with each GO-Term. **e** GO-Term enrichment analysis of significantly downregulated genes (FDR < 0.05, 10 nM DEA) with predicted miR-1229-3p 7mer or 8mer sites in their 3’UTR that are expressed in human neurons (logcpm > 2.5 in the pLNA sequencing dataset). Displayed are the 10 most significant “Cellular Component” GO-Terms ( > 15 annotated genes) and the number of the corresponding sign. downregulated genes (red bars). **f** Expression values (logcpm, left) and predicted canonical miR-1229-3p binding sites in the 3’UTR (right) of five sign. downregulated genes in SH-Sy5y cells transfected with a control (Ctrl.) or miR-1229-3p mimic (10 nM, 20 nM) as determined by RNA-seq. The binding site panels display log(K_D_) values (ScanMiR^86^), the site type and the respective 3’UTR position. **g** Luciferase measurement of Synj2bp 3’UTR constructs (2.3Kb fragment) containing wild-type or mutant miR-1229-3p binding sites, transfected into HEK-cells together with synthetical miR-1229-3p or control (Ctrl.) mimics. **h** Working model of the proposed miR-1229-3p effects leading to a repression of synapse development in human neurons. (* = *p*-value < 0.05). Further information on statistical analyses, the number of biological replicates and exact *p*-values are provided in the Supplementary Data S1. Panel (**h**) was created using BioRender. Wüst, T. (2026) https://BioRender.com/3715lkn.

The observed reductions in mitochondrial gene expression upon prolonged inhibition of miR-1229-3p are likely a consequence rather than a cause of mitochondrial dysfunction. To obtain a first insight into possible direct targets of miR-1229-3p upstream of mitochondrial stress, we performed a more acute manipulation of miR-1229-3p by expressing a miR-1229-3p hairpin construct at two different concentrations in a human neuroblastoma cell line (SH-Sy5y), followed by polyA RNA-seq 48 h later. Differential gene expression analysis revealed a total of 425 DEGs (202 upregulated & 223 downregulated, Fig. 6a). Motif enrichment analysis using EnrichMiR^39^ identified the miR-1229-3p seed match as the overall most enriched motif in downregulated genes at both concentrations (Fig. 6b and Supplementary Fig. S28a). Moreover, we observe a downregulation of mRNAs containing miR-1229-3p motifs depending on the strength of the binding site compared to the “no site” control group (Fig. 6c and Supplementary Fig. S28b). Together, these results confirm that our approach led to an effective downregulation of genes containing miR-1229-3p binding sites in their 3’UTRs. GO-Term and gene set enrichment analysis (GSEA) revealed several pathways associated with metabolism, the endoplasmic reticulum (ER) and neuronal differentiation (Fig. 5d and Supplementary Fig. S29). With a more refined analysis on the group of DEGs containing miR-1229-3p binding sites (Fig. 6d), we identified several terms associated with synapse development, which is consistent with our previous morphological results from miR-1229-3p inhibition in human iNeurons. In conclusion, miR-1229-3p overexpression leads to a downregulation of genes associated with neuronal development, the ER and metabolism.

To get a better understanding of the involved pathways, we took a closer look at some of the most significantly downregulated putative miR-1229-3p target genes (Fig. 6f, Supplementary Fig. S30 and Supplementary Data S8). Several of them are implicated in synapse formation and stability (e.g., *Dlgap4*, *Add2*, *Pak3*), mitochondrial function and homeostasis (e.g., *Eif4g2*, *Hipk2*, *Slc25a20*, *Mtor*) and mitochondrial-ER contact sites (MERCS; e.g., *Synj2bp*, *Mtx3*, *Rtn1*, *Arl6ip1*). Considering the importance of MERCS for mitochondrial calcium-signaling^40^, *Synj2bp* represents a particularly interesting candidate. The 3’UTR of *Synj2bp* contains three 7mer or 8mer miR-1229-3p sites, and *Synj2bp* has been previously shown to enhance the formation of MERCS^41,42^. Using luciferase assays, we validated *Synj2bp* as a direct miR-1229-3p target gene (Fig. 6g), raising the possibility that it could represent an important node in a network of genes controlling miR-1229-3p mediated changes in mitochondrial function. In conclusion, our results suggest that miR-1229-3p controls the expression of an entire network of genes which together coordinate synapse development and mitochondrial homeostasis during neuronal maturation (Fig. 6h).

## Discussion

The prolonged synaptogenesis period in specific areas of the human brain is likely one of the most important determinants of human-specific cognitive and behavioral traits. In this regard, the development of cellular systems (2D, 3D) that mimic human brain development has greatly facilitated the discovery of human-specific aspects of synaptogenesis. Here, we modified a neuronal differentiation protocol allowing us to profile ncRNA expression during the time course of human excitatory synapse development at unprecedented resolution (the full datasets are available at https://ethz-ins.org/igNeuronsTimeCourse). Thereby, we observed widespread dynamic regulation of several classes of ncRNAs during this period, highlighting the potential importance of the ncRNA regulatory layer in human brain development. By focusing on dynamically expressed, human-specific miRNAs, we identified miR-1229-3p as a potential regulator of developmental timing of excitatory synaptogenesis.

Four point-mutations within the *miR-1229* gene, which occurred at the transition from great ape to human, allowed its efficient processing to a mature miRNA. The fact that miR-1229 is a mirtron and bypasses the regulatory constraints of DROSHA mediated processing might have facilitated this evolutionary adaptation^43^. As most of the recently evolved miRNAs, miR-1229-3p is lowly expressed and therefore likely fine-tunes the expression of many targets within common cellular pathways^44^, a notion that fits well with the regulation of mitochondrial homeostasis by miR-1229-3p.

The synapse-promoting effect of miR-1229-3p depletion at early time points is apparently at odds with concurrent mitochondrial phenotypes (e.g., mitochondrial fragmentation, loss of mtDNA, reduced mitophagy), which are usually associated with mitochondrial stress. Interestingly, recent data suggests that many of these mitochondrial phenotypes are linked to the interaction of mitochondria with the ER, one of the cellular organelles that seems particularly affected by the overexpression of miR-1229-3p (Fig. 6). MERCs are important for mtDNA replication^45,46^, and ER stress has been associated with mitochondrial DNA release^31^. Further, MERCs have been connected with mitochondrial fission^47^ and fusion^48^, providing a direct link to the regulation of mitochondrial morphology. Finally, it has been postulated that ER stress, specifically in the context of neurodegenerative diseases, can inhibit autophagy^49^, and MERCs are essential for mitochondrial calcium signaling^40^.

Notably, the interaction between mitochondria and the ER is highly dynamic^40^, and there is a significant temporal component in the progression of the ER stress response. Accordingly, it has been shown that increased interactions of mitochondria with the ER during early cellular adaptations to ER stress promote mitochondrial metabolism, whereas sustained ER stress leads to a decrease in MERCs and concomitant decline in respiration^40,50^. This temporal component becomes particularly apparent during neuronal differentiation. The unfolded protein response (UPR), a direct consequence of ER stress, initially promotes neuronal development before ultimately causing detrimental effects^51–53^. Hence, increased ER stress poses a possible explanation for increased synaptogenesis at early time points after miR-1229-3p inhibition. We speculate that with advanced neuronal maturation and prolonged miR-1229-3p depletion, mechanisms that compromise mitochondrial function become dominant, thereby causing the observed defective neuronal dendritogenesis and increased cytosolic calcium spike amplitudes (Figs. 3, 5). Consistently, we found that activation of mitochondrial respiration rescues the changes in cytosolic calcium signaling upon miR-1229-3p depletion at a late time point of igNeuron development (Fig. 5). Thus, we speculate that human-specific regulation of MERCs by miR-1229-3p initially increases mitochondrial metabolism, but in the long run slows down mitochondrial respiration and associated neuronal maturation. This interpretation is consistent with a recent study, which showed that reduced mitochondrial metabolism is associated with a delayed maturation of human neurons in comparison to mouse neurons^30^. Future experiments should therefore more directly assess the role of miR-1229-3p in the induction and maintenance of the ER stress response and its consequences for mitochondrial homeostasis. Further, it will be important to assess whether altering mitochondrial metabolism is able to rescue the aberrant neuronal maturation in miR-1229-3p depleted human igNeurons.

Based on our transcriptomic results upon miR-1229-3p overexpression, it becomes apparent that miR-1229-3p regulates an entire network of genes associated with different aspects of mitochondrial homeostasis, the ER, as well as synapse formation and stability, likely concertedly causing the observed cellular phenotypes. Since many of the mitochondrial results obtained in igNeurons upon miR-1229-3p depletion can be linked to the abundance of MERCs, it was striking to see that some of the putative miR-1229 targets, such as *Synj2bp*^41^ and *Mtx3*^54^, are directly involved in the regulation of MERCs. Particularly, *Synj2bp* is an interesting candidate. It contains various strong miR-1229-3p binding sites, and its overexpression has been shown to increase the number of MERCs^41^. While interactions between the ER and mitochondria regulate several important processes in both organelles^40^, it has been shown that a sustained increased number of MERCs can cause mitochondrial dysfunction^55^.

Moreover, *Synj2bp* functions as an mRNA anchor at the outer mitochondrial membrane, thereby promoting the local translation of *Pink1*^56,57^. *Pink1* was among the few upregulated genes upon miR-1229-3p depletion containing a miR-1229-3p binding site and could be validated as a direct miR-1229-3p target (Supplementary Fig. S31). *Pink1* has been previously linked directly to synapse development^58^. Hence, it is possible that *Synj2bp* and *Pink1* collectively promote synapse development during early stages of igNeuron development upon miR-1229-3p knockdown, although the exact molecular mechanisms downstream of miR-1229-3p have to be determined in future studies.

From a clinical point of view, a SNP within the *miR-1229* gene that is present in around 3% of the human population (Supplementary Fig. S32) was recently associated with increased risk for Alzheimer’s disease^59^. Since mitochondrial fragmentation and mitophagy deficiencies have been implicated in Alzheimer’s disease and several other neurodegenerative disorders^60,61^, investigating the role of miR-1229-3p-target interactions in such conditions might be a promising direction for future research.

## Methods

### Research conducted for this manuscript complies with all relevant ethical regulations

#### Cell culture – iPSC Maintenance

Most experiments were performed with the SANi002-A induced pluripotent stem cell (iPSC) line (originated from a female donor)^62^. Ca-imaging was additionally conducted on MML6838.Cl2 cells (originated from a male donor)^63^. iPS cells were cultured on Matrigel (Corning, #354277) or Geltrex (Thermo - A1413302) in mTeSR Plus (StemCell # 100-0276). Cells were fed according to the weekend-free manufacturer’s protocol. Single-cell splitting was generally conducted every 6-8 days using TrypLE (Thermo 12604013). Negative scraping was performed to assure healthy stem cell morphology. Before counting, cells were manually dissociated using a serological pipette. Thiazovivin (Calbiochem - 420220) was added to the media for 24 h upon single-cell splitting in a concentration of 2 µM to improve the growth and proliferation of single cells. iPSCs were usually split a few times to assure robust proliferation before starting neuronal differentiations.

#### Cell culture – “igNeuron” differentiation

Differentiation of human neurons was done mainly following the protocol of Zhang et al.^20^. with a few modifications inspired by Qi et al.^64^, Nehme et al.^65^ and Ho et al.^66^. In brief, human neurons were cultured on PLL-Laminin instead of Matrigel (Supplementary Fig. S1a). Additionally, to accelerate their maturation, cultured neurons were supplemented with glia-derived growth factors (GDNF + CNTF) (Supplementary Fig. S1b). Finally, the growth inhibitor AraC was titrated in low concentration into the culture medium to eliminate the generation of neural progenitor cell clusters (Supplementary Fig. S1c).

To start a differentiation 80k to 150k stem cells (depending on cell confluency and proliferation), were split into each well of a 6-well plate on Day -2 (coated with Matrigel). Lentiviruses (pTet-O-Ngn2-puro and FUW-M2rtTA) were added on Day -1 after washing the cells with DMEM-F12. Viruses were acquired from the viral vector facility (VVF) at the UZH Zurich and tested for their efficiency with puromycin titration experiments. Cells were usually washed with DMEM-F12 16–18 h after virus addition before adding neuronal induction media (see media overview). The day after (day 1), the media was exchanged with new neuronal induction media containing puromycin in a concentration that would kill all cells without resistance (0.5 μg/ml). See the supplementary methods for exact media compositions. On day 2, cells were released with Accutase (Innovative Cell Technologies #AT104, distributed by StemCell), spun down, and resuspended in neuronal culture media I. Dissociation was conducted manually with a serological pipet. Around 80k cells were then seeded in 500 μl medium on a PLL-Lam coated coverslip (CVS) in a 24-well plate. In 6-well plates, around 350–400 k neurons were seeded in 2 ml medium on CVS coated with PLL-Lam. For the mitochondrial super-resolution imaging, 270k neurons were seeded on PLL-Lam coated ibidi dishes (Ibidi – 81158).

Before coating, CVS were treated with Boric Buffer (0.1 M Boric Acid, pH 8.5) over night, followed by thorough washing with water and baking overnight in a drying oven. PLL was diluted to 0.1 mg/ml in 0.1 M Boric Acid, and 400 μl of this dilution was added to each well in a 24-well plate (2 ml for 6-well plates or ibidi dishes). Subsequent incubation over night at 37 °C and washing with H2O, Laminin was added in a final concentration of 3.4 μg/ml and incubated at 37 °C for an hour. Finally, CVS (or ibidi dishes) were washed 2*x* with H2O and equilibrated with NB Plus right before usage.

An additional 300 μl of neuronal culture medium I (per well in a 24-well plate, 1 ml per well in a 6-well plate) were added to the neuronal cultures at day 5. AraC was added to the media on day 6, by collecting 510 μl of each 24-well, adding 310 μl fresh neuronal culture medium I and then adding AraC into this media mix at a final concentration of 50 nM. The remaining media was exchanged very carefully with the newly prepared AraC media. Neurons were subsequently fed every 3-4 days with neuronal culture medium II. Neuronal culture medium was stored for maximal 7 days at 4 °C, media supplements were aliquoted and only thawed before preparation of the media (besides Mouse Laminin and AraC that were kept at 4° degree for maximal one month).

To determine mtDNA content as well as to quantify mitochondrial morphology at day 25, we used iND3 neurons that were a kind gift of Mathias Müller from Novartis. These neurons were differentiated from IPS cells with a stably integrated doxycycline inducible NGN2-cassette that was introduced via the piggyBac system (see ref. ^67^). Neuronal differentiation was then induced by culturing these iPS cells for three days in a proliferation medium containing DMEM/F12 (Invitrogen – 31331), Pen/Strep (Invitrogen – 15070), B27 with Vit. A (Invitrogen - 17504-044), N2 supplements (Invitrogen - 17502-048), bhFGF at 10 ng/ml (Invitrogen – CTP 0261), hEGF at 10 ng/ml (Invitrogen – PHG 0315) and Doxycycline at 1 mg/L (Sigma D9891). At day 3, neurons were frozen and from then on cultured on PLL / Lam-coated dishes or coverslips in neuronal culture medium I. The day after thawing was counted as day 4.

#### Cell culture – pLNA addition

Custom miRCURY LNA Power Inhibitors (pLNAs) antisense to the respective microRNA were acquired from Qiagen (purified by HPLC + NA + salt exchange, Cat. Nr.: 339146). The corresponding Negative Control A (pLNA-Ctrl.) was used as a control. pLNAs were added in a concentration of ~ 100 nM at day 9 for igNeurons and at day 10 for iND3 neurons. Per 24-well, 330 μl media was collected, supplemented with 170 μl fresh media and the respective pLNA. The rest of the culture media (in fact, almost all of it to assure that wells didn’t get fully empty, which would cause neurons to detach) was then exchanged carefully with the prepared media-pLNA mix.

#### Cell culture – Mitochondrial activators

GSK-2837808A (diluted in DMSO to a 10 mM stock solution - 5 μM final concentration) and Albumax (diluted in H2O - 0.5% final concentration) were added from day 16 on, similarly as described for the pLNAs. DMSO and H2O were used for the control conditions. They were subsequently added freshly to the media before each feeding.

#### Cell culture – HEK cells

HEK-293 cells (Merck – 85120602-1VL) were cultured in DMEM with high glucose, no pyruvate, with glutamine (Thermo – 41965039) and 10% FBS (Fisher Scientific – 10500064). Pen-Strep was added to the media in a concentration of 1:100 (100U). Splitting of HEK cells was done using TrypLE (Thermo 12604013).

#### Cell culture – SH-Sy5y cells

SH-Sy5y neuroblastoma cells (ECACC) were cultured in DMEM/F12 with Glutamax (Invitrogen – 31331028), 10% heat inactivaed Fetal Bovine Serum (Fisher Scientific – 10500064) and Pen/Strep (100 U/ml). Splitting was performed every three to four days using TryplE (Thermo 12604013).

#### Cell culture – primary cortex neurons

Primary neurons were essentially cultured and prepared as described in Lackinger et al.^68^. Culturing medium consisted of Thermo NB Plus / B27 Plus (A3653401) supplemented with additional Glutamax (1.5 mM final concentration – add 1 mM to NB Plus) and Pen-Strep in a concentration of 1:100 (100U).

#### Luciferase measurements

Luciferase assays were performed with a modified dual luciferase pmirGLO vector containing two SV40 promoters in front of the renilla and firefly coding regions. *Pink1* 3’UTR (NM_032409.3) was amplified from gDNA (*Pink1* genome primers, see primer list in the supplementary methods) and then cloned into the multiple cloning site downstream of the firefly gene (see primer list). Seed mutations were introduced by mutagenesis PCR as described in https://human.bio.lmu.de/_webtools/MINtool/MINTagging_Protocol.pdf^69^. For the *Synj2bp* experiment, a region encompassing roughly 2.3 kb in the 3’UTR was amplified from gDNA (*Synj2bp* genome primers, see primer list) and then likewise cloned into the pmirGLO vector. In order to introduce mutations into the three canonical miR-1229-3p binding sites (plus two high-affinity non-canonical binding sites) in that region, we ordered the amplified *Synj2bp* fragment encompassing 2-3 point mutations in each of these binding sites from Thermo Fisher (see supplementary methods) and cloned it also into the pmirGLO reporter. Perfect binding site reporters comprising two fully complementary perfect binding sites separated by two nucleotides were likewise cloned into the pmirGLO vector at said position (see primer list). The miR-1229 precursor overexpression construct (minigene containing pre-miR-1229 and 1-2 surrounding exons) was cloned into a pcDNA3 vector under the control of a CMV promoter with primers designed according to Butkyte et al.^70^ (see primer list). Chimp mutations were introduced by cut and paste cloning of an ordered DNA fragment (Thermo, GeneArt - 815010DE) containing the respective mutations. Constructs were transfected into HEK cells or primary cortical neurons (at DIV 6) using Lipofectamine. Perfect binding site experiments were performed using 50 ng of reporter and 10pmol of synthetical miRNA mimic or control respectively. Cells were lysed 48 h after the transfection. For the Pink1 experiments, 50 ng of reporter was transfected into HEK cells and 100 ng of reporter into primary rat neurons. For the *Synj2bp* experiment, 50 ng of reporter was transfected together with 10pmol of mimic into HEK cells. HEK cells were lysed after 96 h, and rat cortical cells 72 h following the transfection. miR-1229 precursor experiments were conducted with 50 ng of perfect binding site reporter and 450 ng minigene constructs. Cells were lysed 96 h after the transfection. For all experiments in which HEK cells were lysed 96 h following the transfection, a medium change to DMEM (incl. Pen-Strep) without FBS was conducted 24 h after the transfection.

Measurements were performed on a Promega GloMax Luminometer with homemade solutions according to Baker & Boyce^71^. Before the assay, cells were lysed with passive lysis buffer (Promega - E1941) and then shaken at RT with low speed while luciferase solutions equilibrate.

#### Immunocytochemistry and FISH

Stainings were performed on neurons fixed with 4% PFA (including sucrose). Fixation time was generally limited to 10 min to assure intact post-synaptic compartments. Subsequent washing with PBS (washing must be performed extremely carefully to not lose the cells), CVS were transferred to a wet staining chamber and blocked and permeabilized with a Triton (0.1%) – NGS (10%) solution (PBS was used as dilutor), usually for 15 min. Neurons were incubated with primary antibodies (diluted in said Triton-NGS solution) for 90 min. Following 5 additional wash steps (alternating fast and 10 min washes), neurons were incubated for 45 min with the secondary antibody (diluted in Triton-NGS) in the dark. Afterwards, CVS were again washed 5 times (alternating fast and 10 min washes) and mounted to imaging slides with Aqua-Poly/Mount (Polysciences – 18606). We generally waited at least 24 h before imaging the CVS. Antibodies in the following dilutions were used for the experiments: MAP2 (Thermo - PA1-16751, 1:2000), SYN1 (Merck - AB1543, 1:1000), PSD95 (Biolegend – 810401, 1:200), LAMP2 (Novus biologicals - NB300-591, 1:250), TUJ1 (Lucerna-Chem / Covance - 801201 // MMS-435P-100, 1:2500), SOX2 (Merck - MAB4343, 1:1000), GFAP (Antibody has been a gift, 1:1000) and MAP2 (Sigma - M9942, 1:4000). Secondary antibodies include Hoechst, Phalloidin (Abcam - ab176759, 1:1000) and Thermo Alexa Fluor secondary antibodies diluted 1:1000 (specifically 647-goat-anti-chicken (A-21449), 546-goat-anti-chicken (A-11040), 488-donkey-anti-mouse (A-21202), 647 goat-anti-rabbit (A27040) and 488-goat-anti-rabbit (A-11008). Some secondary antibodies were at some point switched to the “Alexa Plus” version.

Single-molecule small RNA FISH was performed using the ViewRNA Cell Plus kit according to the manufacturers protocol (Thermo - 88-19000-99) with a probe for miR-1229-3p (VM1-33156-VCP) and miR-218-5p (VM1-10092-VCP). For miR-1229-3p, an EDC cross-labeling step was added before the protocol (Thermo – 22980), using solutions of the Affymetrix QuantiGene® ViewRNA miRNA ISH Cell Assay kit and following the manufacturer's described steps.

### Mitochondrial super resolution live-cell imaging (morphology & mtDNA content)

To image neuronal mitochondria at the super-resolution microscope (SIM – Zeiss Elyra 7, or iSIM - visitech), igNeurons were cultured on PLL-Laminin coated Ibidi Dishes (Ibidi – 81158). For the morphology experiments at day 29 and day 36 igNeurons were incubated for ~ 20 min with PKMO (1:1000) (PKmito Orange, SpiroChrome #SC053)^72^ and then washed once with PBS. Imaging was performed in Leibovitz’s L-15 Medium (Thermo – 21083027). 50 frames with an interval of 2 s were recorded for most videos. Postprocessing was done either with the SIM or the SIM^2^ algorithm from Zeiss.

Mitochondrial DNA content and morphology at day 25 were determined together in iND3 neurons. For this experiment, we stained neurons with SYBR Gold (Thermo Fisher - S11494, 1:50’000) and PKMDR (PKmito Deep Red, Spirochrome #SC055, 1:1’000) for roughly 30-35 min, followed by at least another hour of post-incubation. Imaging was performed in neuronal culture medium without phenol red, doxycycline and cAMP (Neurobasal minus phenol red (Thermo – 12348017) was used as the basis for the medium). We acquired z-stack images in live at the iSIM microscope for the quantification.

### mtKeima experiment

A mtKEIMA Lentivirus (LV-hCMV-(mt)mKeima-WPRE) produced by the VVF of the UZH Zurich (Addgene #131626) was added to the culture media at day 13 with a physical titer of roughly 1537 ng/µl. Imaging was conducted at day 23. The imaging buffer was prepared as Neuronal culture medium 1 without phenol red and cAMP (see above). Neurons were washed carefully with PBS, and then 1 mL of imaging buffer was added. Images were recorded on an inverted Nikon spinning disk microscope equipped with the Yokogawa Confocal Scanner Unit CSU-W1-T2 SoRa and a triggered Piezo z-stage (Mad City Labs Nano-Drive). It was used in spinning disk mode with a pinhole diameter of 50 µm combined with a 1.45 NA, 100*x* objective and controlled by the NIS Elements Software (Nikon). Images were acquired with a sCMOS Hamamatsu Orca Fusion BT camera (2304 × 2304 pixel, 6.5 × 6.5 µm pixel size). Imaging was performed at 37 °C on a single slice using 405nm excitation (neutral) and 561nm excitation (acidic), with a long-pass 600 nm filter for both. Regions of interest were found using the brightfield mode, and brightfield views were collected for soma annotation during the analysis.

### TMRE experiment

Mitochondria were imaged for membrane potential at day 23. TMRE dye was added at a concentration of 50 nM for ca. 30 min. Following the incubation, the cells were washed once with PBS and then imaged in the same imaging medium as for the mtKeima and mitochondrial calcium and mtDNA experiment at 37 °C on the iSIM microscope (visitech).

### Image analysis

For the synapse co-cluster analysis, images were acquired on a Zeiss LSM 880 airyscan confocal microscope. Images to quantify dendritogenesis were taken on a Zeiss Axio Observer 7 microscope.

Image analysis was performed using custom-developed cell profiler pipelines. The synapse pipeline was inspired by Nieland et al.^73^, the dendritogenesis pipeline by Tian et al.^74^. Cell Profiler pipelines can be found in the GitHub repository.

In brief, for the synapse co-cluster analysis, somata were identified based on the Hoechst and MAP2 signal, followed by a segmentation of the dendrites as well as PSD95 and SYN1 puncta. Subsequently, PSD95 was masked by the slightly extended dendritic area (to include synaptic protrusions), and SYN1 was masked by the remaining PSD95 puncta. Manual comparisons of detected puncta and true co-clusters in the original images were used to optimize parameters. Random colocalizations of rotated images were included to assure specificity of the analysis. Finally, the detected number of co-clusters is normalized by the dendritic area in each image. For the time course analysis, two coverslips were imaged per time point and differentiation, yielding a total of 236 images. For the pLNA experiment, 64 images were acquired per condition and time point in total, taken from two CVS each.

For the dendritogenesis analysis, somata in 20*x* tile pictures were identified by Hoechst and Map2. Subsequently, a propagation algorithm was used to extend dendritic signals from the soma along MAP2. Individual neurons were then skeletonized and individually measured. For the time course analysis, two coverslips were imaged per time point and differentiation, yielding a total of 120 images. We acquired 32 tile images per condition and time point in total for the pLNA experiment.

Quantification of mitochondria imaged with the SIM microscope was conducted by selecting a single image frame of the videos and segmenting as well as thresholding mitochondria based on the PKMO signal. For the length quantification, segmented mitochondria were skeletonized.

For the mtKeima experiment, we manually counted acidic mitochondria (small mitochondria without signal in the neutral channel) in the neuronal processes. In order to normalize for the neuronal and mitochondrial density, we additionally segmented mitochondria with Ilastik. Acidic mitochondria were then normalized by the number of neutral mitochondria identified in each image. The somata in each image were manually excluded. For the ratio, the mean acidic intensity of each mitochondria was divided by the sum of neutral + acidic intensity (ac / ac + neu). Replicates with a low mtKeima signal intensity were excluded from the analysis.

TMRE quantifications were performed on segmentations of mitochondria and the somata (generated with Ilastik) on the maximum intensity projections of recorded z-stacks. Ilastik probabilities were then processed with CellProfiler to get quantifiable objects. Subsequently, we measured the mean intensity of the TMRE channel within those objects, and separated mitochondria in segmented somata and mitochondria in processes (outside of the segmented somata). Finally, mitochondria in processes with mean intensities that differed more than five times the standard deviation from the mean TMRE intensities of mitochondria in processes in each differentiation were filtered out.

For the combined mitochondrial morphology and mtDNA content experiment, we segmented deconvoluted images (using Huygens, standard deconvolution) with Ilastik. Mitochondria were identified based on the PKMDR channel and mtDNA puncta based on the SYBR Gold channel. Exported probabilities from Ilastik were further processed with CellProfiler. Mitochondrial DNA (mtDNA) puncta were classified as “within” mitochondria in case they showed at least a 10% overlap with the mitochondrial segmentation.

### Calcium imaging

A Gcamp6f AAV-virus (pscAAV-DJ8/2-hCMV-chI-GCaMP6f-SV40p(A) produced by the VVF of the UZH Zurich (from Addgene #51083)) was added to the culture media 7 days before imaging with a physical titer of roughly 4.125 × 10^9 vg/ml. The imaging buffer was prepared according to Barreto-Chang & Dolmetsch^75^, including NaCl (129 mM), KCL (5 mM), CaCl2 (2 mM), MgCl2 (1 mM), Glucose (30 mM) and HEPES (25 mM). The imaging buffer was adjusted to a pH of 7.4 and supplemented with 5% BSA diluted in PBS right before imaging.

Neurons were washed carefully with PBS and then transferred to an imaging chamber containing the imaging buffer. Before the recordings, cells were incubated for ~ 10 min in the imaging buffer to assure equilibration. Videos were recorded at a Zeiss Axio Observer 7 widefield microscope (20x) at low light intensity for 5 min with an interval between frames of roughly 400 ms. Generally, two CVS were imaged, and 5 videos at different locations recorded from each CVS (exceptions were cell line 2 (day 15) during the time course experiment and differentiation M1 in the mitochondrial activation experiment, for which videos were recorded from only one CVS).

For the analysis, intensity values of cells were extracted with FIJI using a custom script. Transduced cells were manually selected based on a maximum intensity projection of the video. Trace tables were further analyzed using the custom-programmed SpikeIt R shiny app (see code availability) to extract peaks of the fluorescent signal normalized to the median of the baseline signal (Fluorescence – median (Fluorescence) / median (Fluorescence)) = df/F). The stringency for the peak detection was set to 3 in the SpikeIt app. In addition, only cells that spiked with a minimum relative amplitude of 7.5% were considered for the analysis.

### Electrophysiology

Whole cell patch clamp recordings were performed on an upright microscope (Olympus BX51WI) at room temperature. Data were collected with an Axon MultiClamp 700B amplifier and a Digidata 1550B digitizer. Analysis was performed with the pClamp 11 software (all from Molecular Devices). Recording pipettes were pulled from borosilicate capillary glass (Harvard Apparatus; GC150F-10) with a DMZ-Universal-Electrode-Puller (Zeitz) and had resistances between 3 and 5 MW.

Spontaneous EPSCs (sEPSCs) were recorded from igNeurons at Day 40–42. The extracellular solution (ACSF) was composed of (in mM) 140 NaCl, 2.5 KCl, 10 HEPES, 2 CaCl_2_, 2 MgCl_2_, 10 glucose (adjusted to pH 7.3 with NaOH). The intracellular solution contained (in mM) 125 K-Gluconate, 20 KCL, 0.5 EGTA, 10 HEPES, 4 Mg-ATP, 0.3 GTP and 10 Na_2_-Phosphocreatine (adjusted to pH 7.3 with KOH). Cells were held at -60 mV. The sampling frequency was 10 kHz and the filter frequency 2 kHz. Series resistance was monitored, and recordings were discarded if the series resistance changed significantly ( > 15%) or exceeded 25 MΩ.

Neurons cultured for the electrophysiology experiments were treated with the mitochondrial activator control conditions.

### Lactate measurements

Lactate measurements were performed from igNeuron media supernatants with the Lactate-Glo assay from Promega (J5021) according to the manufacturer’s protocol. Luminescence was recorded on a Promega GloMax Luminometer with an integration time of 0.3 s. Before measuring, samples were diluted 1:100 in PBS to assure that lactate concentrations would fall in the linear range of the assay. For differentiations M1-M3, media supernatants were collected from the Ibidi dishes that were used for the mitochondrial super-resolution live cell imaging. For differentiation M4, media supernatant was collected from 3*x* wells of a 24-well plate and pooled together (neurons for this differentiation were treated with the mitochondrial activator controls).

### RNA methods

The miRVana kit (Thermo - AM1561) was used according to the manufacturer’s protocol for RNA extraction. Whenever possible, RNA was extracted from two wells of a 6-well plate and pooled together. Extractions for different time points or conditions were done together (cells were initially lysed, and the lysate then frozen until the final extraction was conducted). DNase treatment (Turbo DNase, Thermo - AM2238) and column cleanup (RNeasy MinElute Cleanup Kit, Qiagen – 74204) were performed before samples were sent for ribosomal depletion and polyA sequencing.

For the miR-1229-3p overexpression experiment in SH-Sy5y cells, we transfected miR-1229-3p precursors (Thermo, Ambion - PM13382) and the corresponding control (Thermo, Ambion - AM17110) using Lipofectamine 2000 (Thermo – 11668027). Specifically, cells were split and seeded into 6wells at a density of ca 200k cells per well. We then transfected them on the next day in the following conditions: control condition (40pmol of Neg. Ctrl.), miR-1229 10 nM condition (20pmol of Neg. Ctrl. & 20pmol of miR-1229-3p precursor) and the miR-1229 20 nM condition (40pmol miR-1229-3p precursor). This experimental design allowed the comparison of two different miRNA concentrations while using just a single control condition. For RNA extraction, we subsequently lysed the cells ca. 48 h after transfection with the miRVana lysis buffer and extracted the RNA of all replicates together as described above.

miRNA qPCRs were performed with TaqMan primers essentially as described in the manufacturer’s protocol (Thermo 4366596 & Thermo 4427975), except that 40–50 ng were used for the initial reverse transcription and then diluted to 1:4 / 1:5 before the qPCR. Samples were measured on a BioRad CFX384 Real-Time system with an elongation time of 45 s. Expression values were normalized to U6.

Small RNA sequencing and ribosomal depletion sequencing (for the time course experiment) were performed by the functional genomics center in Zurich. We got at least 75 million reads per sample (100 bp read length) for the ribosomal depletion sequencing and 3–20 million reads per sample for the small RNA sequencing (only one sample had three million reads, most samples had around or above 10 million reads). Illumina TruSeq RNA stranded and Ribosomal Depletion Ribozero gold kits were used for the ribosomal depletion library construction, Illumina TruSeq Small RNA kit was used for the small RNA library preparation. For the polyA sequencing, RNA was sent to Novogene in London who performed library construction (using the NEB Next Ultra RNA Library Prep Kit for Illumina) and paired end sequencing. Libraries for the pLNA sequencing had to be amplified with the Takara SMARTer amplification kit due to low input.

### miRNA expression in post-mortem samples of Danish psychiatric patients

The samples come from the prefrontal cortex and hippocampus of individuals that were all Scandinavian Caucasians with no history of alcohol and drug misuse. They were collected in accordance with Danish law and with the consent of the Central Denmark Area Health Research Ethics Committees (license number: M-2017-17-17). Two experienced psychiatrists (AB Bertelsen and R Rosenberg) reassessed and verified the diagnoses by carefully reviewing all medical records and comparing the diagnoses with the modern criteria of DSM-IV (Diagnostic and Statistical Manual of Mental Disorders, 4th Edition) and ICD-10 criteria (The International Statistical Classification of Diseases and Related Health Problems, 10th Edition). In the present study we included patients diagnosed with schizophrenia (SCZ), bipolar disorder (BD). RNA extraction was performed as described previously^76^.

All samples were diluted to a final miRNA concentration of 8 ng/µl with DEPC water before cDNA synthesis, which was carried using the miRCURY LNA RT kit (Cat no. 339340, Qiagen) according to the manufacturer´s instructions (except that we used 3 µl of each sample and therefore added 3.5 µl of water to reach a final volume of 10 µl). The cDNA samples were stored undiluted at − 80 °C until real-time qPCR analysis.

Real-time qPCR was carried out on individual samples in 96-well PCR-plates using the Agilent AriaMx system (AH Diagnostic) and the miRCURY LNA SYBR Green PCR kit (Cat no 339347, Qiagen). The samples were diluted 1:40 before being used as a template. All samples were run in singlets. A standard curve and no template controls, performed in duplicate, was generated on every plate. Each SYBR Green reaction (10 µl total volume) contained 2*x* miRCURY SYBR® Green Master Mix (5 µl), 1 µl resuspended primer mix, 1 µl DEPC water, and 3 µl of diluted cDNA. The PCR conditions were as follows: 95 °C 2 min, 95 °C 10 sec and 56 °C 1 min (40 cycles), 95 °C 30 sec, 65 °C 30 sec, 95 °C 30 sec.

The real-time qPCR efficiency varied from 91–112% in the hippocampus and from 103–108% in the prefrontal cortex, respectively. Samples with Cq values above 30 were excluded from the analysis.

### Label-free proteomics

#### Protein extraction and preparation

igNeurons grown on glass coverslips in one well of a 6well plate were carefully washed once with ice-cold PBS before, before being lysed with 200 μl RIPA buffer (150 mM NaCl, 1% Triton X-100, 0.5% Sodiumdeoxycholate, 1 mM EDTA, 1 mM EGTA, 0.05% SDS, 50 mM Tris pH 8.0) containing EDTA-free protease inhibitor (Roche, distributed by Merck 11873580001). Cells were scratched off the surface and transferred to an Eppendorf tube, which was subsequently shaken for 20 min (50 rpm) in the cold room at 4 °C. Before further processing, samples were snap-frozen with liquid nitrogen and stored at − 80 °C. Proteins were collected from three independent differentiations at seven time points. All samples collected were then spun down together for 30 min at 4 °C. The supernatant was transferred to a new tube, and protein concentrations were measured using the Pierce BCA assay (Thermo - 23225). 11.5 μg protein were then precipitated with 4% TCA (Trichloracetic acid) according to Ngo, Ezoulin, Youm, & Youan^77^. In brief, samples were mixed and then incubated for 35 min at 4 °C. Afterward, they were centrifugated for 5 min at 4 °C with 15’000 g and the obtained pellets washed with 500 μl ice-cold acetone. These washing steps were repeated once before pellets were dried and then further processed for the protein digest and clean-up.

#### Protein digest and clean-up

Protein extracts were further processed with a filter-assisted sample preparation protocol^78^. 30 µl of SDS denaturation buffer (4% SDS (w/v), 100 mM Tris/HCL pH 8.2, 0.1 M DTT) was added to each protein pellet. For denaturation, samples were incubated at 95 °C for 5 min. Samples were then diluted with 200 µl UA buffer (8 M urea, 100 mM Tris/HCl pH 8.2) and subsequently loaded to regenerated cellulose centrifugal filter units (Microcon 30, Merck Millipore, Billercia MA, USA). Samples were spun at 14000 × *g* at 35 °C for 20 min. Filter units were washed once with 200 μL of UA buffer, followed by centrifugation at 14000 × *g* at 35 °C for 15 min. Cysteines were alkylated with 100 µl freshly prepared IAA solution (0.05 M iodoacetamide in UA buffer) for 1 min at room temperature in a thermomixer at 600 rpm followed by centrifugation at 14000 × *g* at 35 °C for 10 min. Filter units were washed 3 times with 100 µl of UA buffer and then twice with a 0.5 M NaCl solution in water (each washing was followed by centrifugation at 35 °C and 14000 × *g* for 10 min). Proteins were digested overnight at room temperature with a 1:50 ratio of sequencing grade modified trypsin (0.4 µg, V511A, Promega, Fitchburg WI) in 130 µl TEAB (0.05 M Triethylammoniumbicarbonate in water). After this overnight protein digestion, peptide solutions were spun down at 14000 g at 35 °C for 15 min.

#### Peptides Clean-up

Peptides were cleaned up using the Phoenix kit (Preomics) according to the manufacturers protocol. Samples were lyophilized in a SpeedVac and then re-solubilized in 19 µl 3% ACN / 0.1% FA (formic acid) prior to LC-MS/MS measurement. 1 µl of synthetic iRT peptides (Biognosys AG, Switzerland) were added to each sample for retention time calibration.

#### LC-MS/MS measurements

Samples were measured on a QExactive Mass Spectrometer (Thermo Fisher Scientific, Waltham MA, USA). Peptides were separated with an Eksigent NanoLC (AB Sciex, Washington, USA). We used a single-pump trapping 75-µm scale configuration (Waters). 1 µl of each sample were injected. Trapping was performed on a nanoEaseTM symmetry C18 column (pore size 100 Å, particle size 5 µm, inner diameter 180 µm, length 20 mm). For separation, a nanoEaseTM HSS C18 T3 column was used (pore size 100 Å, particle size 1.8 µm, inner diameter 75 µm, length 250 mm, heated to 50 °C). Peptides were separated using a 120 min long linear solvent gradient of 5-35% ACN / 0.1% FA (using a flowrate of 300 nl / min). Electronspray ionization with 2.6 kV was used, and a DIA method with a MS1 in each cycle followed by 35 fixed 20 Da precursor isolation windows within a precursor range of 400–1100 m/z was applied. For MS1, we used a maximum injection time of 200 ms and an AGC target of 3e6 with a resolution of 60 k in the range of 350–1500 m/z. MS2 spectra were acquired using a maximum injection time of 55 ms and an AGC target of 1e6 with a 30 k resolution. A collision energy of 28 was used for fragmentation.

#### Protein search and quantification

We used SpectronautTM (Biognosys, version 19) with directDIA for peak picking as well as sequence assignment and a reference proteome for *H. Sapiens* from Uniprot (2024-05). We included a maximum of 2 missed cleavages, using a Tryptic in-silico digest with a KR/P cutting profile. Sequences in a range of 7-52 AA were considered. We included carbamidomethyl as a fixed modification for cysteine, oxidation as a variable modification for methionine and protein N-terminal acetylation as a variable modification. Decoys were generated using a scrambled label-free decoy method. A kernel density estimator was used with a 1% FDR for q-value filtering. A maximum of 5 variable modifications were considered. Single hits were determined on the stripped sequence level. Major grouping was done by protein group ID, and minor grouping by stripped sequence. Only proteotypic peptide sequences were considered, and single hit proteins excluded. For the minor and major group quantification, the top 3 entries were considered, using the mean precursor/peptide quantity. A localized normalization strategy and interference correction were used. Machine learning was performed on a per run basis, and iRT profiling was enabled.

### Bioinformatic analyses

Scripts used in the bioinformatic analyses are provided in a GitHub repository (see data availability), hence here follows just a brief outline of the individual methods.

### Ribosomal depletion sequencing of the time course dataset

Read mapping was performed with STAR^79^ on GRCh38.p10 (Ensembl Annotation 91) with the following parameters: --outFilterMatchNmin 30 --outFilterMismatchNmax 10 --outFilterMismatchNoverLmax 0.05 --alignSJDBoverhangMin 1 --alignSJoverhangMin 8 --alignIntronMax 100000 --alignMatesGapMax 100000 --outFilterMultimapNmax 50 --chimSegmentMin 15 --chimJunctionOverhangMin 15 --chimScoreMin 15 --chimScoreSeparation 10 --chimOutType Junctions SeparateSAMold --outSAMstrandField intronMotif --alignEndsProtrude 3 ConcordantPair -- getJunctionstrue

FeatureCounts^80^ was used to get read counts. Differential expression statistical analysis was performed using edgeR^81^ with a linear model over all time points, including an added effect for the first experiment since that seemed to be a slight outlier from the PCA plot. Only genes with at least 10 reads in at least 2 samples were considered.

### Small RNA sequencing analysis of the time course dataset

Small RNA detection was performed using Oasis^82^ on the human genome annotation hg38. Differential expression analysis was conducted with edgeR, employing a linear model over all time points. Only small RNAs with at least 20 counts in at least one sample were considered for the analysis.

### Clustering of the ribosomal depletion time course dataset

Clustering and cluster enrichment analysis of the significantly changing genes (FDR < 0.01 & logFC > 2) were performed using custom scripts. In brief, a matrix of median log2 foldchanges per timepoint was created, capped at the 0.95 percentile of absolute log2FC in each direction, and absolute values below 0.5 were set to zero. A signed increment of 0.25 was then added to prevent genes changing in different directions being clustered together. Clustering was performed using Partitioning Around Medoids (pam) at multiple values of k, and a value was then manually selected based on average silhouette width, separation, and maximum dissimilarity. Gene clusters were further characterized by identifying the Gene Ontology terms whose members were the most heterogeneously distributed across clusters, as measured by the binomial deviation.

### Clustering of the small RNA time course dataset

To cluster significantly changing small RNAs (FDR < 0.05) of the short RNA data, the logcpm quantification was first corrected using the RUVs method^83^ with two latent variables, and a per-timepoint smoothed mean was computed by fitting a loess model on each miRNA against the day as a numeric value. Binarized log2 foldchanges (minimum 0.1) relative to the first timepoint were computed from these values and correlated across miRNAs as well as combined to the correlation of per miRNA z-scores to establish a distance matrix between miRNAs. miRNAs were then clustered using the walktrap method with 15 steps from the igraph package on a weighted, undirected graph of the 15 nearest neighbors.

### Statistical comparison of the small RNA sequencing data to morphology

Means of the synapse density analysis (per 10 μm area) were shifted one time point earlier, since the mRNA changes precede the morphological changes by roughly one time point, and one would expect that miRNAs act on mRNAs. The actual mean values per time point were then used as linear model input ( ~ values) for a subsequent differential expression analysis of the small RNA sequencing dataset.

### Proteomics analysis of the time course dataset

Protein expression data obtained with spectronaut was imported in R. Values were first normalized by variance stabilizing transformation using the vsn package (version 3.54.0), and missing values were imputed using the MinProb function (*q*-value < 0.01) of the DEP package (version 1.8.0). Differential expression analysis was performed using limma (eBayes(lmFit) and topTable) over the whole time course, as well as comparing neighboring time points to each other.

### RNA Protein correlations

Significantly changing genes of each dataset were analyzed for RNA-protein correlations (FDR < 0.01). The gene name was used to detect common instances in both datasets.

### circRNA analysis (time course dataset)

circRNAs were quantified by running CIRCexplorer 2.3.8 (without any special parameters) on the chimeric reads from the alignments of the ribosomal depletion sequencing.

### Small RNA sequencing analysis of the mouse dataset from Whipple et al.^84^

Small RNA detection was performed using Oasis on the mouse genome annotation mm10. A custom adapter sequence from the methods part of Whipple et al.^84^ was indicated. Default advanced options were chosen. Differential expression analysis between day 0 and day 10 was performed using edgeR, including floxed and miR379-410 ko samples with an interaction term ( ~ day*genotype). Only genes with at least 20 reads in one sample were considered.

### Ribosomal Depletion sequencing analysis of the mouse dataset from Whipple et al.^84^

Mapping and read count analysis were performed using salmon. Reads were mapped onto GRCm38.p6 (Gencode release M20 – 2019). Differential expression analysis between day 0 and day 10 was performed using edgeR, including floxed and miR379-410 ko samples with an interaction term ( ~ day*genotype). Genes were filtered using edgeR’s “filterByExpr” function.

### Staging of the ribosomal depletion sequencing dataset

To obtain information regarding the maturation stages of our dataset, we correlated expression values to datasets generated by Lin et al.^21^, Mayer et al.^23^, and Trevino et al.^22^. We used gene counts and cluster annotations provided by the original authors for these analyses. Counts of the Lin et al., and Trevino et al., datasets were summed across cells with the R package scuttle. We then correlated individually expression values of commonly expressed genes between each of the three datasets and our time course experiment, following a mean correction. For the staging analysis, we remapped our time course sequencing dataset onto GRCh38.p13 (Ensembl annotation 112) using salmon^85^ and aggregated to the gene level by mergingthe gene symbol and ensemble identifier.

To compute correlations, we filtered for cellular time points with at least 1 M reads and correlated TPM values of our time course experiment with the count values summed across cells, normalized to the total counts in each cellular time point for the Trevino et al. dataset. For the Lin et al. dataset, we correlated TPM values to counts per million. In the Mayer et al. dataset, we included cellular timepoints with at least 5 M reads, and correlated counts per million of our dataset to normalized counts per million, since Mayer et al. used the SMARTer Ultra Low RNA kit, which does not specifically amplify the 3’end for single-cell sequencing.

### Target conservation analysis

3’UTRs were extracted from human genome annotation hg38 and lifted over to the chimpanzee genome PanTro6 using rtracklayer. Only transcripts expressed in iNeurons were considered. Small gaps with a maximal break of 10nt were merged. Subsequently, human and chimp sequences were scanned with scanMiR^86^ for binding sites of e.g., mature hsa-miR-1229-3p.

### polyA sequencing analysis of the pLNA and SH-Sy5y dataset

Reads for both datasets were mapped onto GRCh38.p13 (Ensembl annotation 107) and counted using salmon^85^. Aggregation to the gene level was performed based on gene symbols.

In the pLNA-sequencing dataset, one sample was a clear outlier in the PCA plot as well as moderately degraded according to the SMARTer amplification QC and thus excluded. Reads were filtered by expression (edgeR: filterByExpr). Differential expression analysis was performed using a combined control of the “Empty” and “pLNA-Ctrl” condition with edgeR. We additionally employed three SVA (Surrogate Variable Analysis) correction parameters to account for variability in the dataset.

For the SH-Sy5y dataset, we performed differential expression analysis both, in a combined way using the miR-1229-3p mimic amount (Ctrl., 10 nM and 20 nM; ~ amount) as well as individually testing both overexpression conditions separately against the control (Ctrl.; ~ condition). We again filtered reads by expression (filterByExpr) and employed SVA correction.

### enrichMiR analyses

enrichPlots and cumulative distribution (CD) plots were generated using the enrichMiR package^39^. Siteoverlap and areamir tests were used on a target collection based on canonical miRNA binding sites (7mers & 8mers) in 3’UTRs identified with scanMiR. CD-plots were equally generated with the scanMiR annotation using the option to split by best site type. For further information, please refer to the enrichMiR publication.

### miRNA conservation analysis

miRNA precursors were downloaded from miRbase v22. Reciprocal blast was performed with default settings to identify candidate orthologs in other species. We required the length of hit sequence to be > 60% and < 130% of the query sequence in line with Hu et al.^18^. Genome versions can be found in the respective script. Precursors for which the hit sequence is ≥ 130% of the query sequence were filtered out. Seed mismatches were identified with custom scripts.

Precursors with at least one identified mismatch among primates (excluding marmoset) were subjected to a structural conservation analysis with RNAz, following an idea of McCreight et al.^87^. RNAz gives a structural conservation index as output, which was plotted against the percentage of sequence identity. We ran RNAz on pairwise alignments for human vs all other species and then selected the maximum SCI among those comparisons. To evaluate overall conservation, we ran RNAz on the combined alignment of all primate species.

For the secondary structures provided in Fig. 2g, we pasted the human miR-1229 precursor sequence and corresponding chimp sequence (as displayed in Fig. 2h) into the RNAfold web server and predicted the secondary structures using the standard parameters. Displayed is the minimum free energy (MFE) structure.

### SNP analysis

For the single-nucleotide polymorphism (SNP) analysis, STAR^79^ was used to align sequencing reads of the pLNA dataset to GRCh38.p13 (Ensembl 107) with the following parameters: --alignIntronMax 1000000 --alignMatesGapMax 1000000 --alignSJDBoverhangMin 1 --alignSJoverhangMin 8 --outFilterMatchNmin 30 --outFilterMismatchNmax 10 --outFilterMismatchNoverLmax 0.05 --outFilterMultimapNmax 50 --chimSegmentMin 15 --chimJunctionOverhangMin 15 --chimScoreMin 15 --chimScoreSeparation 10 --chimOutType Junctions SeparateSAMold --alignEndsProtrude 3 ConcordantPair.

Samtools mpileup was then used to perform SNP analysis on transcripts that are encoded by the mitochondrial genome, essentially following a pipeline provided by the EMBL institute (https://www.ebi.ac.uk/sites/ebi.ac.uk/files/content.ebi.ac.uk/materials/2014/140217_AgriOmics/dan_bolser_snp_calling.pdf). The results were imported in R using the vcfR package and then analyzed for the mutation probability (element “PL”). In addition, heterozygous and homozygous mutations were counted if they occurred in more than 1/1000 reads.

### GO-Term analysis

Gene-Ontology enrichment analysis was performed using topGO and org.Hs.eg.db (version 3.16) according to the topGO vignette on the symbol gene identifiers of the pLNA and SH-Sy5y datasets (minimum nodeSize = 5). Enrichment statistics of differentially expressed genes against the sequencing background were calculated with the elim algorithm employing Fisher’s exact test. According to the topGO vignette, multiple testing correction was not performed.

### GSEA analysis

Gene set enrichment analysis (GSEA) was performed using the fgsea R package on the GO-Term pathway collection downloaded with msigdbr. We ranked the genes of the differential expression analysis by taking the sign of the logFC multiplied with the negative logarithmic *p-*value (-log10(*p*-value)). Gene sets between a size of 15 and 500 were considered for the analysis.

### Statistical analyses

Statistical analyses were performed with R using linear models (lm) or robust linear models (rlm function of the MASS package). Imaging data was generally aggregated per condition and differentiation by the mean. These values were then evaluated with robust linear models including the differentiation as a fixed effect (except for Fig. 5b, where we show the statistical assessment based on a binomial model over all images). Electrophysiology and calcium imaging recordings were aggregated per cell. We then tested whether cells in one coverslip showed a high intraclass correlation coefficient (ICC). The ICC for all readouts in these two experiments was below 0.4, indicating a poor correlation of the cells per coverslip^88,89^. Hence, for the electrophysiology and calcium imaging experiments, we performed statistical analysis at the cellular level, accounting for the differentiation with a fixed effect.

Post hoc multiple comparisons were conducted with the emmeans package. See Supplementary Data S1 for further statistical information on the main figures.

### Plots

Plots were generated in R using various packages, including ggplot2, ggsignif, ggsci, scales, ComplexHeatmap, viridis, SEtools and sechm. ImageJ was used to adjust contrasts in microscope images as well as to calculate scale bars. Log-fold changes (LogFC) in RNA-sequencing data plots are generally Log2FC. Figure panels 1a, 3d, 5g, 6h & S5 were generated using BioRender.

*All experiments were performed and analyzed blinded, except for the calcium-imaging and mitochondrial live cell imaging experiments that were recorded non-blinded*.

### Reporting summary

Further information on research design is available in the Nature Portfolio Reporting Summary linked to this article.

## Supplementary information

Supplementary Information

Description of Additional Supplementary Files

Supplementary Data 1-8

Reporting Summary

Transparent Peer Review file

## Source data

Source Data 1

Source Data 2

Source Data 3

Source Data 4

Source Data 5

Source Data 6

## Data Availability

RNA sequencing data have been deposited to the GEO database and are available under the accession GSE244444. The mass spectrometry proteomics data have been deposited to the ProteomeXchange Consortium via the PRIDE^90^ partner repository with the dataset identifier PXD045809. The igNeurons time course data can be additionally accessed via: https://ethz-ins.org/igNeuronsTimeCourse Scripts used for data analysis and figure generation (including source and further raw data) can be found at:https://github.com/michasou/Soutschek_et_al.-2026 We generated a stable version of this repository at: 10.5281/zenodo.20109278 RNA sequencing data from other publicly available datasets can be accessed from: Ribosomal depletion and small RNA sequencing data of mouse neuronal differentiations with NGN2 (GSE140838) [https://www.ncbi.nlm.nih.gov/geo/query/acc.cgi?acc=GSE140838]; Single-Cell sequencing data of human NGN2-neurons cultured together with astrocytes (E-MTAB-10632) [https://www.ebi.ac.uk/biostudies/ArrayExpress/studies/E-MTAB-10632?query=E-MTAB-10632]; Single-cell sequencing data of the developing human cortex obtained by Trevino et al. (GSE162170) [https://www.ncbi.nlm.nih.gov/geo/query/acc.cgi?acc=GSE162170] and Single-cell sequencing data of the developing human cortex obtained by Mayer et al. (dbGaP: phs000989) [https://dbgap.ncbi.nlm.nih.gov/beta/study/phs000989.v6.p1/#study]. Source data for the main figures are provided in this paper.

## Code availability

The app to perform calcium imaging analysis can be accessed at: https://ethz-ins.org/SpikeIt.
