# Supplementary material for: A human-specific microRNA controls the timing of excitatory synaptogenesis
Source: Nat Commun. 2026 Jul 6;17:8365. doi: 10.1038/s41467-026-74753-y (PMC13473650; doi:10.1038/s41467-026-74753-y)
Supplement: Supplementary file 1 [file 41467_2026_74753_MOESM1_ESM.pdf]

Supplementary Materials for

A human-specific microRNA controls the timing of excitatory synaptogenesis.

Authors: Michael Soutschek[1,2], Alessandra Lo Bianco[1], Simon Galkin[1], Tatjana Wüst[1], Koen Wentinck[2], David Colameo[1], Tomas Germade[1], Fridolin Gross[1,3], Lukas von Ziegler[4], Johannes Bohacek[4], Betina Elfving[5], Pierre-Luc Germain[1,4,6], Jochen Winterer[1], Tatjana Kleele[2]* and Gerhard Schratt[1]*

*Corresponding authors: gerhard.schratt@hest.ethz.ch; tatjana.kleele@biol.ethz.ch

This PDF file includes:

Supplementary Figs. S1 to S32 (pp. 1-53)
Supplementary Methods (pp. 54-56)
References (p. 57)

Supplementary figure S1)

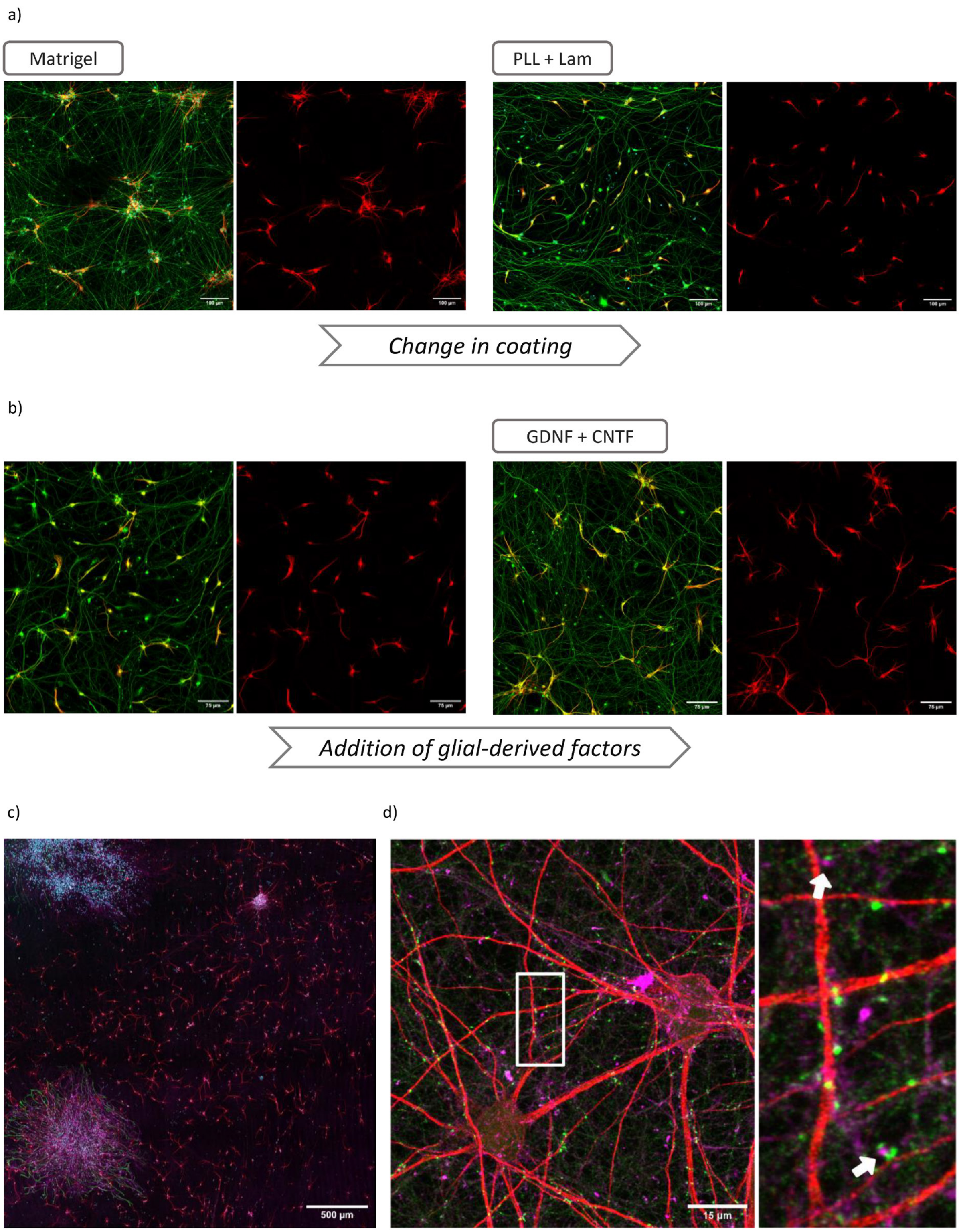

Supplementary figure S1): Optimizations to culture human neurons without glial cells
a) Human neurons cultured on different substrates without the addition of animal glial cells. TUJ1 is displayed in green, MAP2 in red and Hoechst in blue. Left panels: cultures with Matrigel coating (according to the original protocol by Zhang et al (*1*)). Right panels: cultures with PLL-Laminin coating. Neurons were imaged at day 15. When cultured on Matrigel,

neurons cluster together and form “street-like” connections between each other. b) Neurons cultured on PLL-Laminin without (left) or with (right) the addition of glial-derived factors (CNTF & GDNF). Neurons were imaged at day 23. Glial-derived factors increase the maturation stage of human neurons cultured without animal glial cells. c) Human neurons cultured on PLL-Laminin with glial-derived factors but without the addition of AraC. Neuronal precursor stem cell clusters which spontaneously differentiate into glial cells are frequently observed. MAP2 is displayed in red, SOX2 in magenta, GFAP in green and Hoechst in blue. Neurons were imaged at day 29. d) igNeurons show spine-like structures from day 33 on as shown with a co-staining of the F-actin probe phalloidin (magenta), SYN1 (green) and MAP2 (red).

Supplementary figure S2)

a)

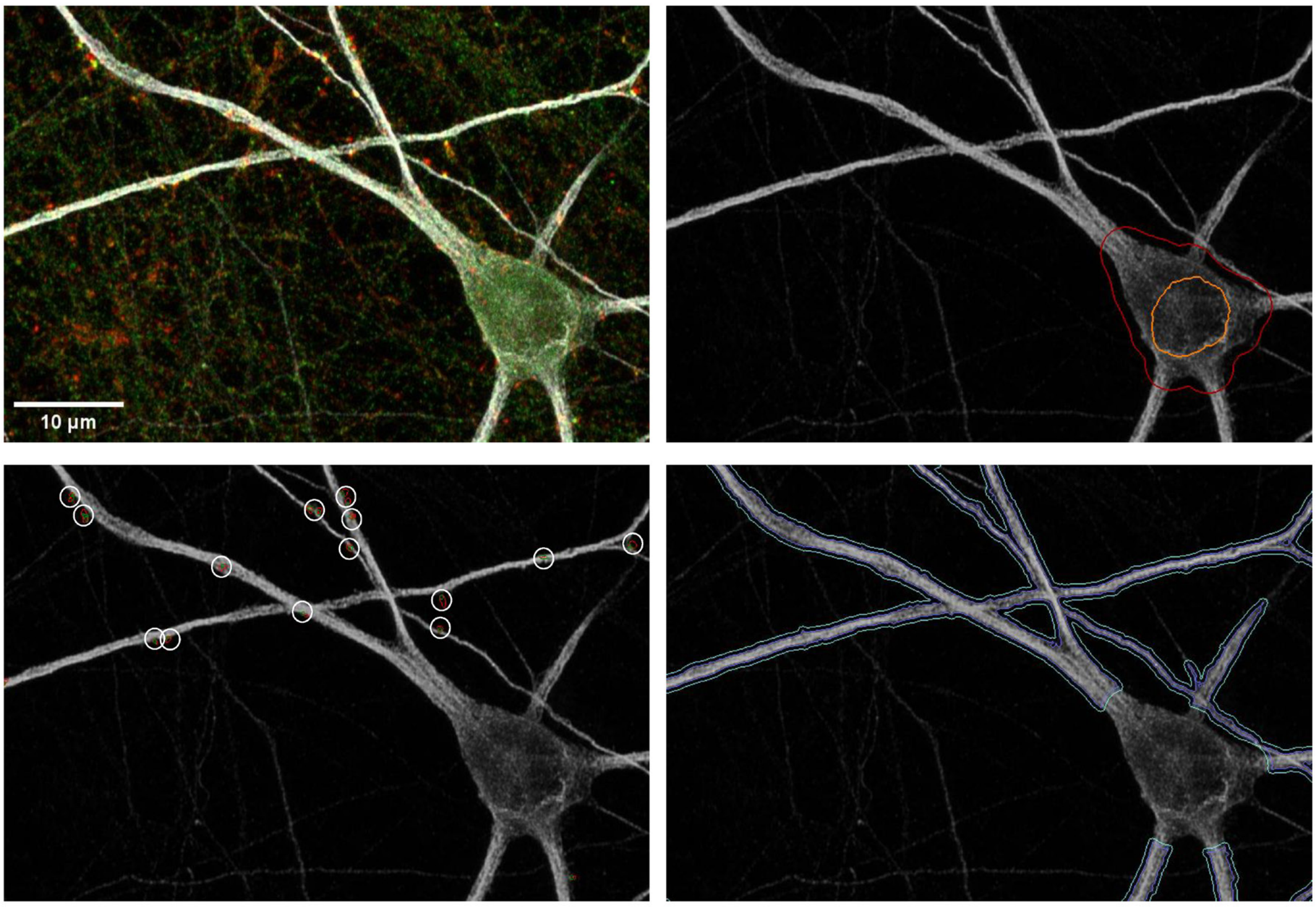

b)

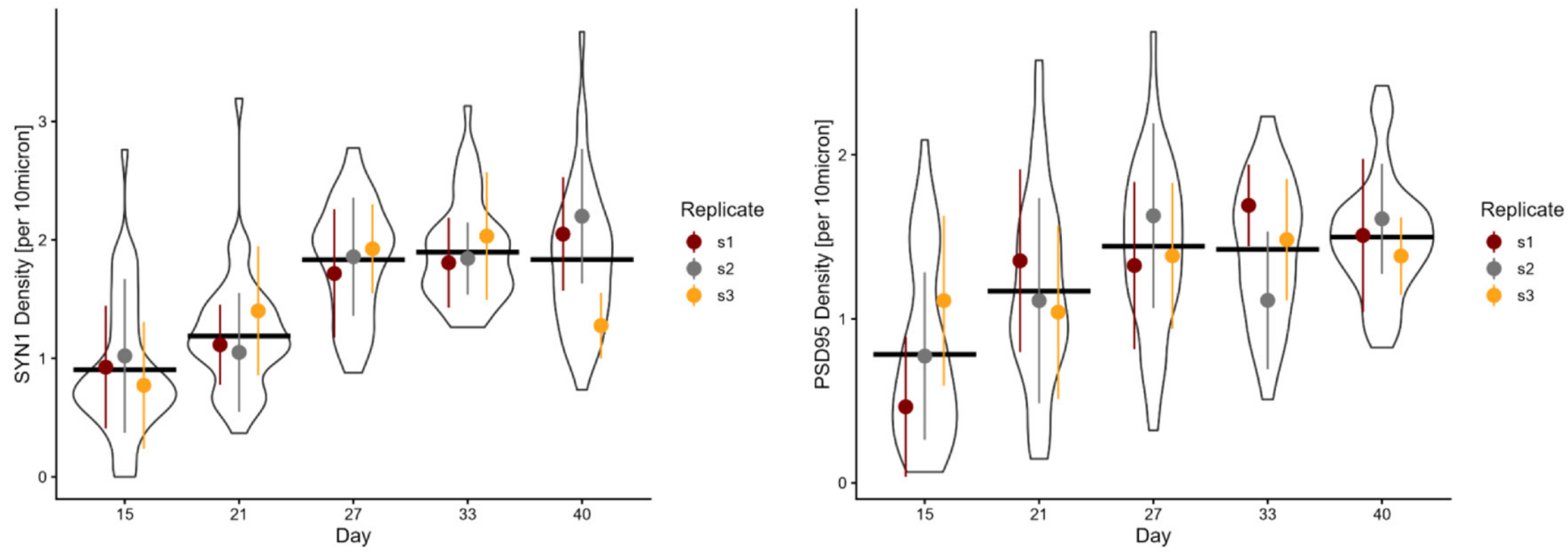

Supplementary figure S2): Synapse co-cluster analysis during human neuronal differentiation

a) Example images illustrating the functionality of the CellProfiler pipeline used for the synapse co-cluster analysis. The top left panel shows parts of a confocal image with MAP2 in gray, SYN1 in red and PSD95 in green. Segmentation of the soma (red) and nucleus (orange) is shown on the top right. The lower right panel indicates the dendrite segmentation (excluding the soma) and detected synaptic co-clusters (overlay of SYN1 and PSD95 at dendrites, marked by circles) are indicated on the lower left. b) Quantification of SYN1 (left) and PSD95 (right) density (separately) over the time course of neuronal differentiation (n=3). Violin plots of all datapoints are shown together with the mean and standard deviation of each differentiation.

Supplementary figure S3)

a)

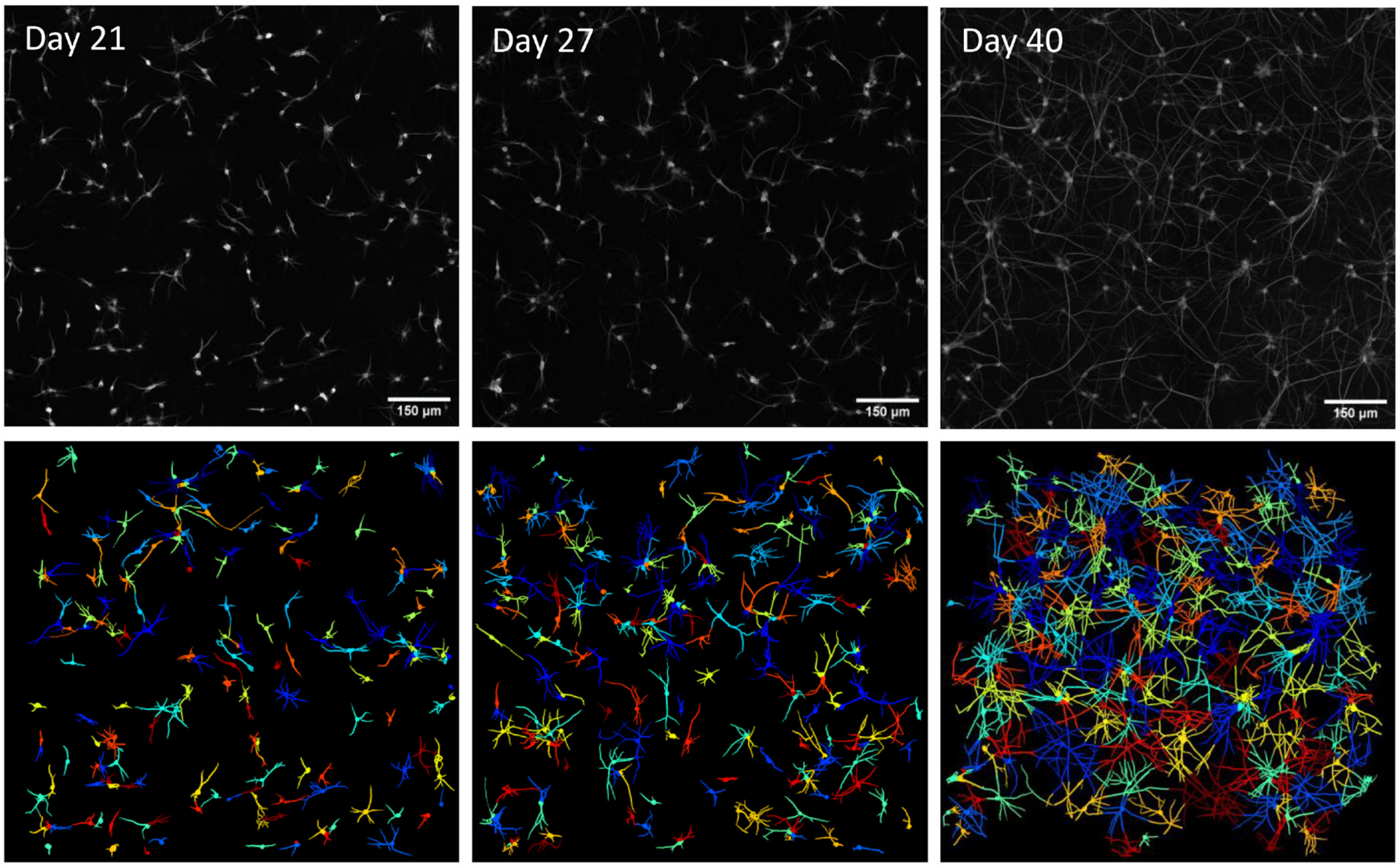

b)

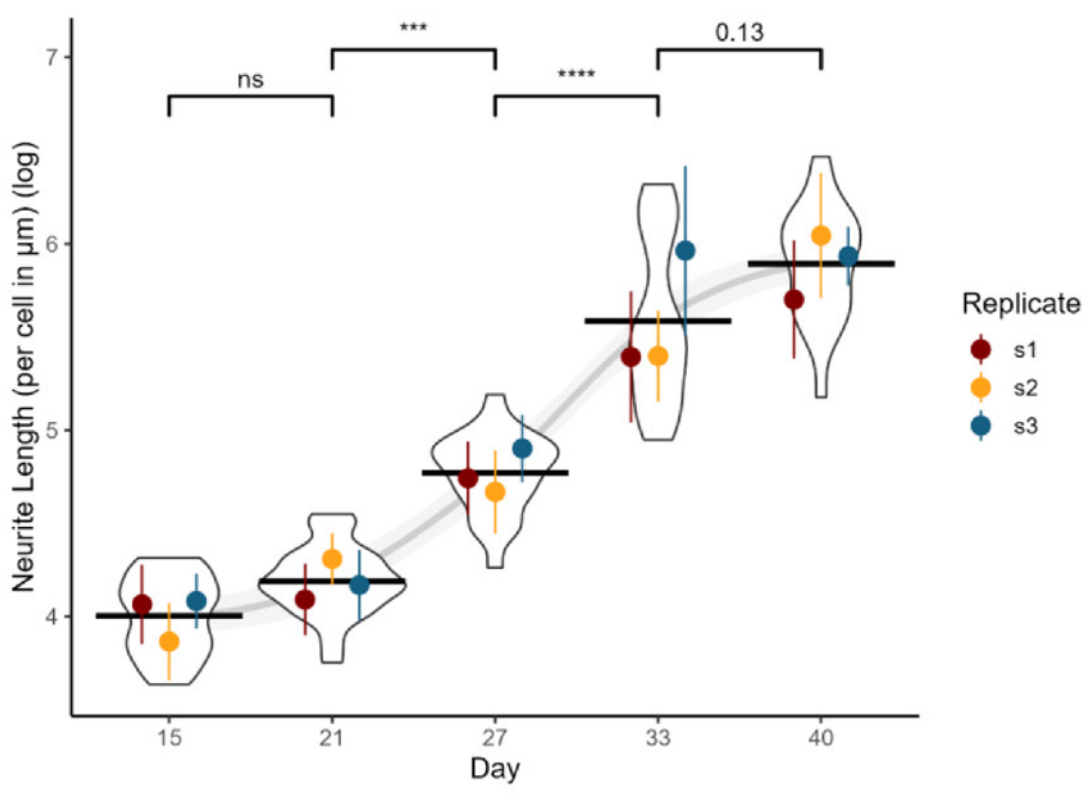

c)

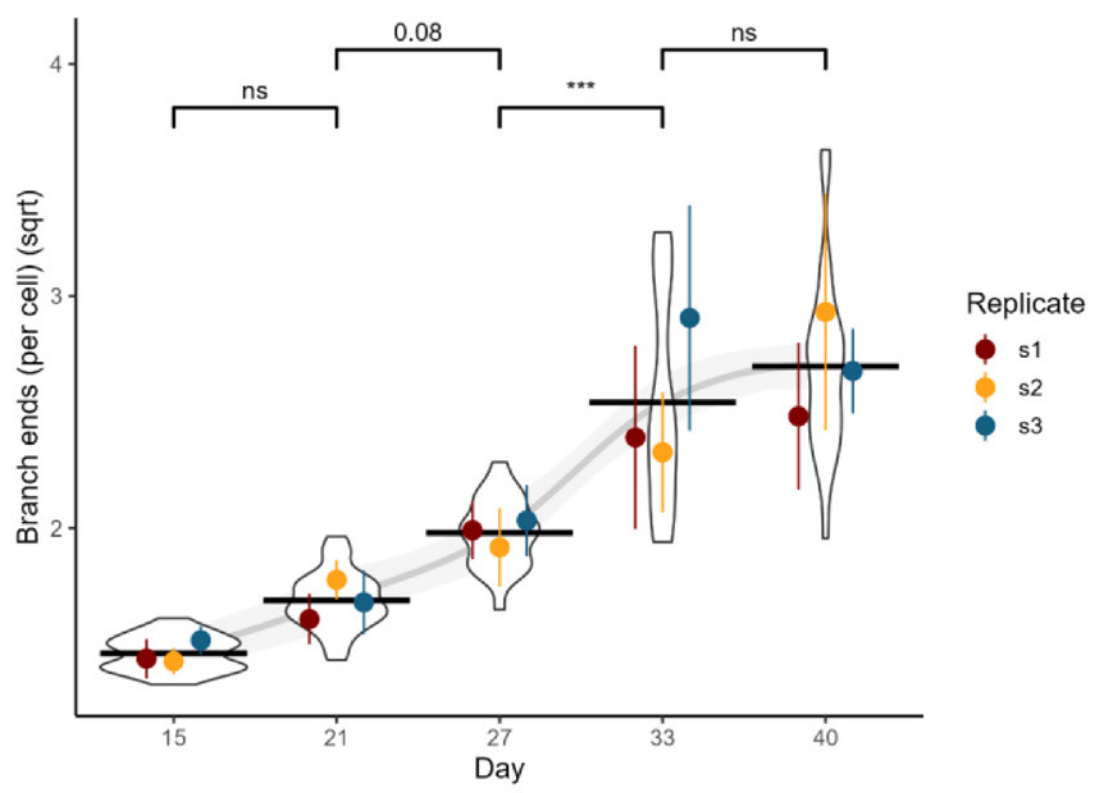

d)

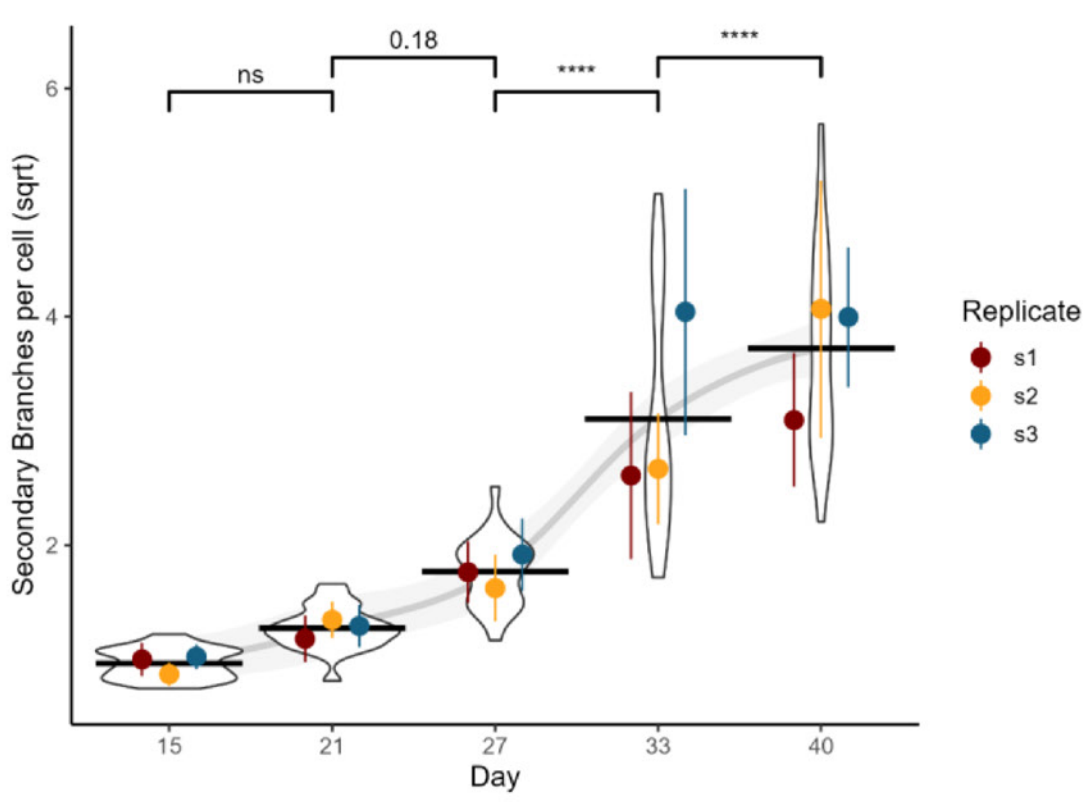

e)

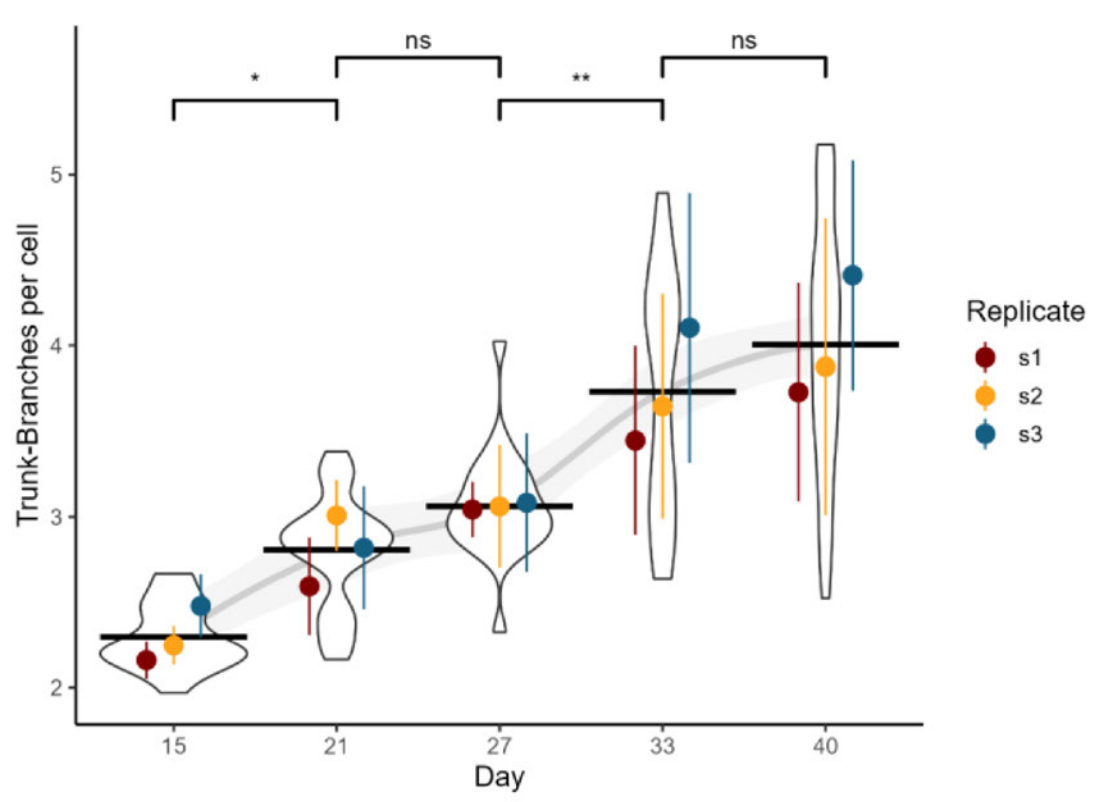

Supplementary figure S3): Dendrite analysis during human neuronal differentiation

a) Example images illustrating the functionality of the CellProfiler pipeline used to quantify dendritic complexity at different time points (20x tile images). The upper row shows the MAP2

signal (gray) of the original images while the lower row displays individual segmented neurons of the same images. b-e) Quantifications of the total neurite length per cell (b), number of branch ends per cell (c), number of secondary branches per cell (d) and the number of trunk branches per cell (originating from the soma) (e) over the time course of neuronal differentiation (n=3). Violin plots of all datapoints are shown together with the mean and standard deviation of each differentiation. Length values were log-transformed (b), the values for branch ends (c) and secondary branches (d) were transformed with the square root. A robust linear model was applied over the aggregated means of each day and differentiation to account for outliers in any of the time points (~ day + differentiation). Statistical comparisons between time points were acquired by applying post-hoc analysis with emmeans. Shown are only statistical comparisons between neighboring time points. (* = p-value < 0.05, ** = p-value < 0.01, *** = p-value < 0.001, **** = p-value < 0.0001)

Supplementary figure S4)

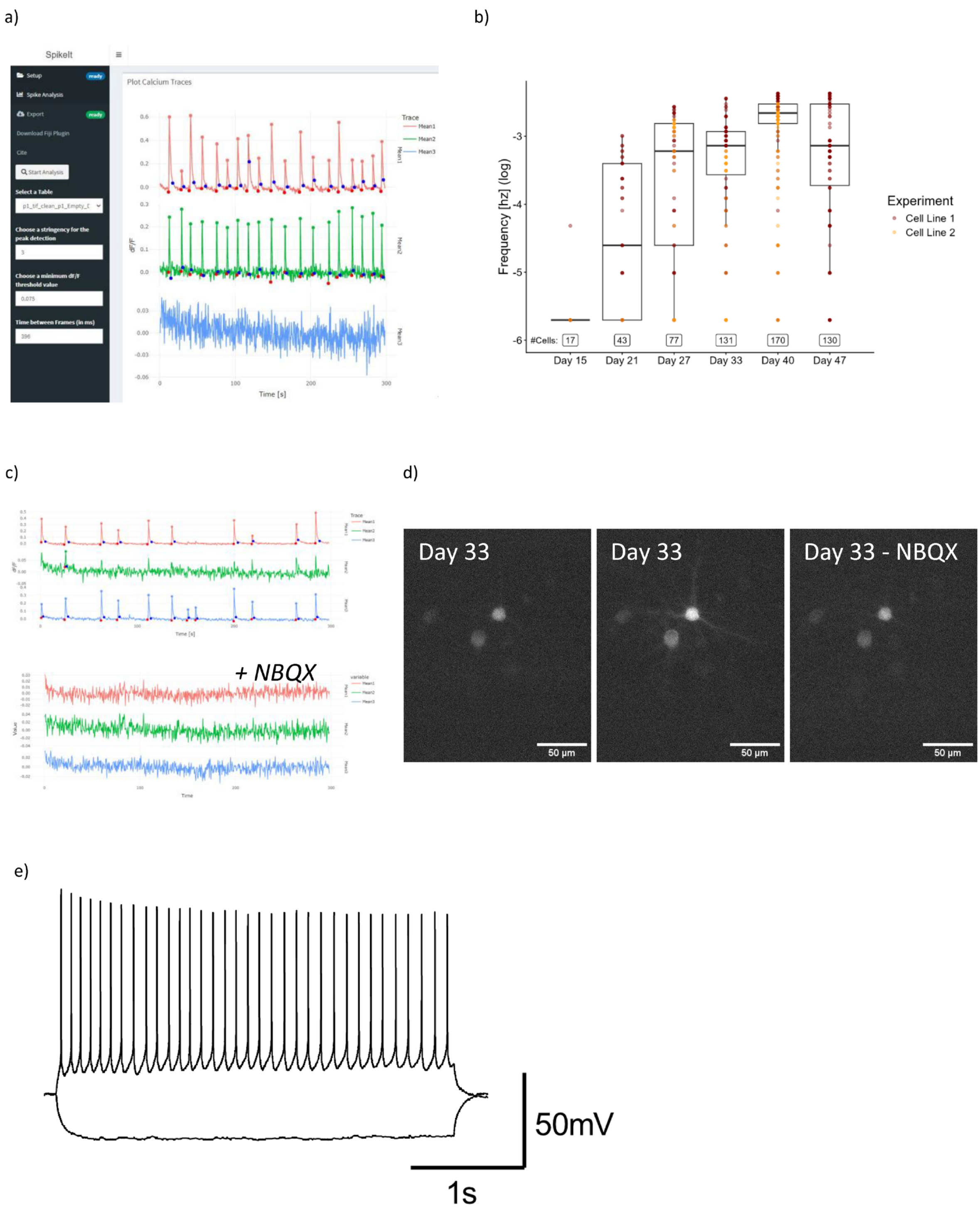

Supplementary figure S4): Measurements of spontaneous activity in igNeurons

a) Screenshot of the custom developed SpikeIt app that was used to analyze Ca-imaging traces of igNeurons. Traces of individual cells are highlighted with different colors. The example snippet shows calcium signals in three different cells (“Mean1”, “Mean2”, ”Mean3”). For more information we refer the reader to the web page of the SpikeIt app: https://ethz-ins.org/SpikeIt/ b) Quantification of the Ca-imaging frequency (log, aggregated per cell) over the time course of neuronal differentiation. Measured were two different cell lines. c) Example Ca-imaging

traces of three cells ("Mean1", "Mean2", "Mean3") detected in one recorded video at day 33 over 300s (including a control pLNA in the medium, cf. Fig. 3). Application of NBQX (20nM) to the imaging chamber resulted in an elimination of calcium spikes, confirming that the signal is dependent on AMPA-receptor activity (the same position was recorded after an incubation time of 5min). d) Snippets of example frames of the videos analyzed in c) illustrate the Ca-signal (gray) detected in igNeurons upon transduction with the calcium indicator GCaMP6f. e) Example trace of action potentials recorded with the patch-clamp technique at day 40 (including a control pLNA in the medium, cf. Fig. 3) upon current injections.

Supplementary figure S5)

a)

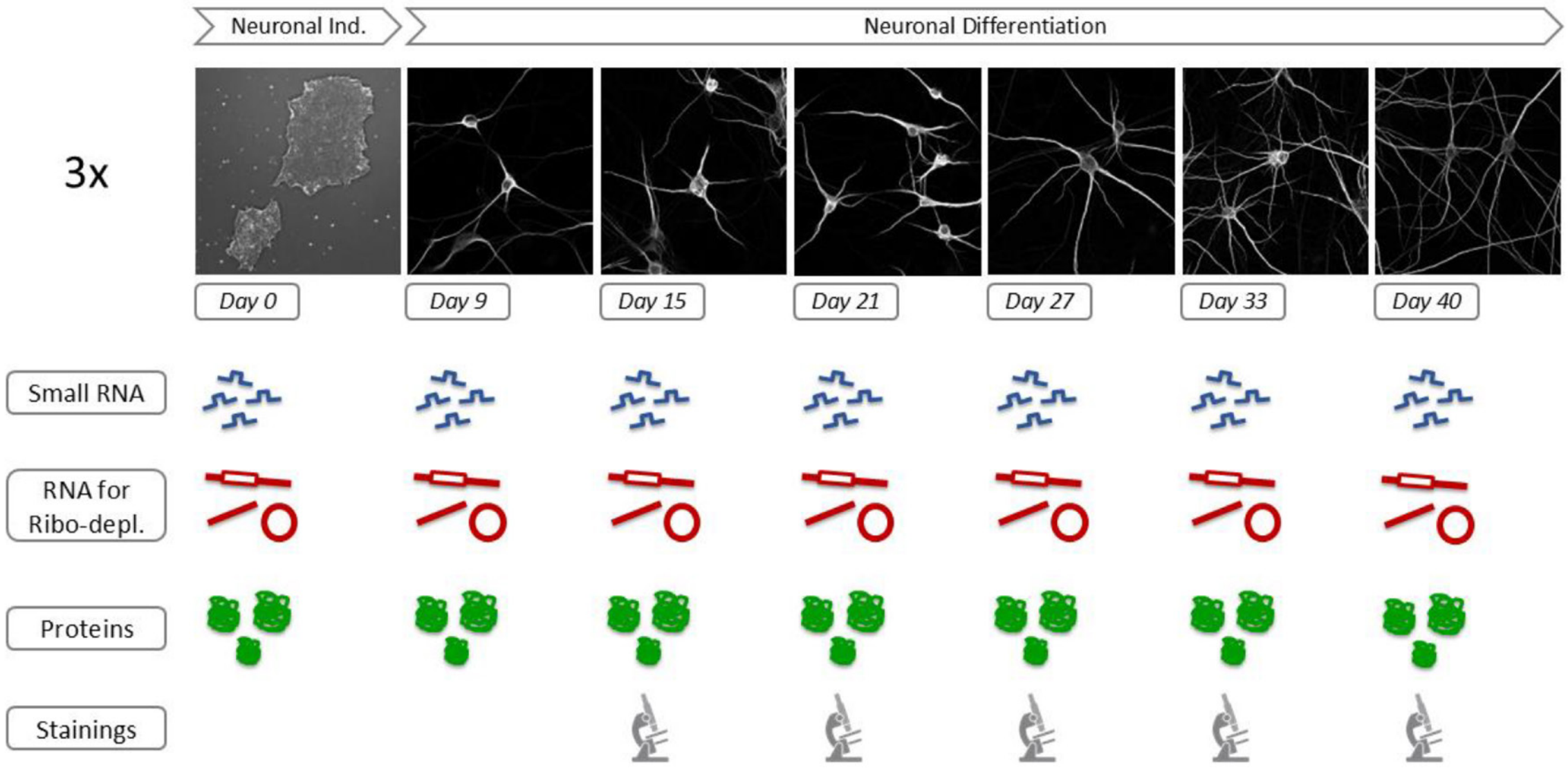

Supplementary figure S5): Time course experiment overview

a) Outline of the developmental time course experiment that was performed to characterize igNeurons along excitatory synapse development. Three independent igNeuron differentiations (replicates) were performed. Neurons of these differentiations were subjected to RNA and protein lysis at seven timepoints, complemented by stainings for MAP2, PSD95 and SYN1 at five time points. This figure was generated using an icon from BioRender. Wüst, T. (2026) https://BioRender.com/evaev7h

Supplementary figure S6)

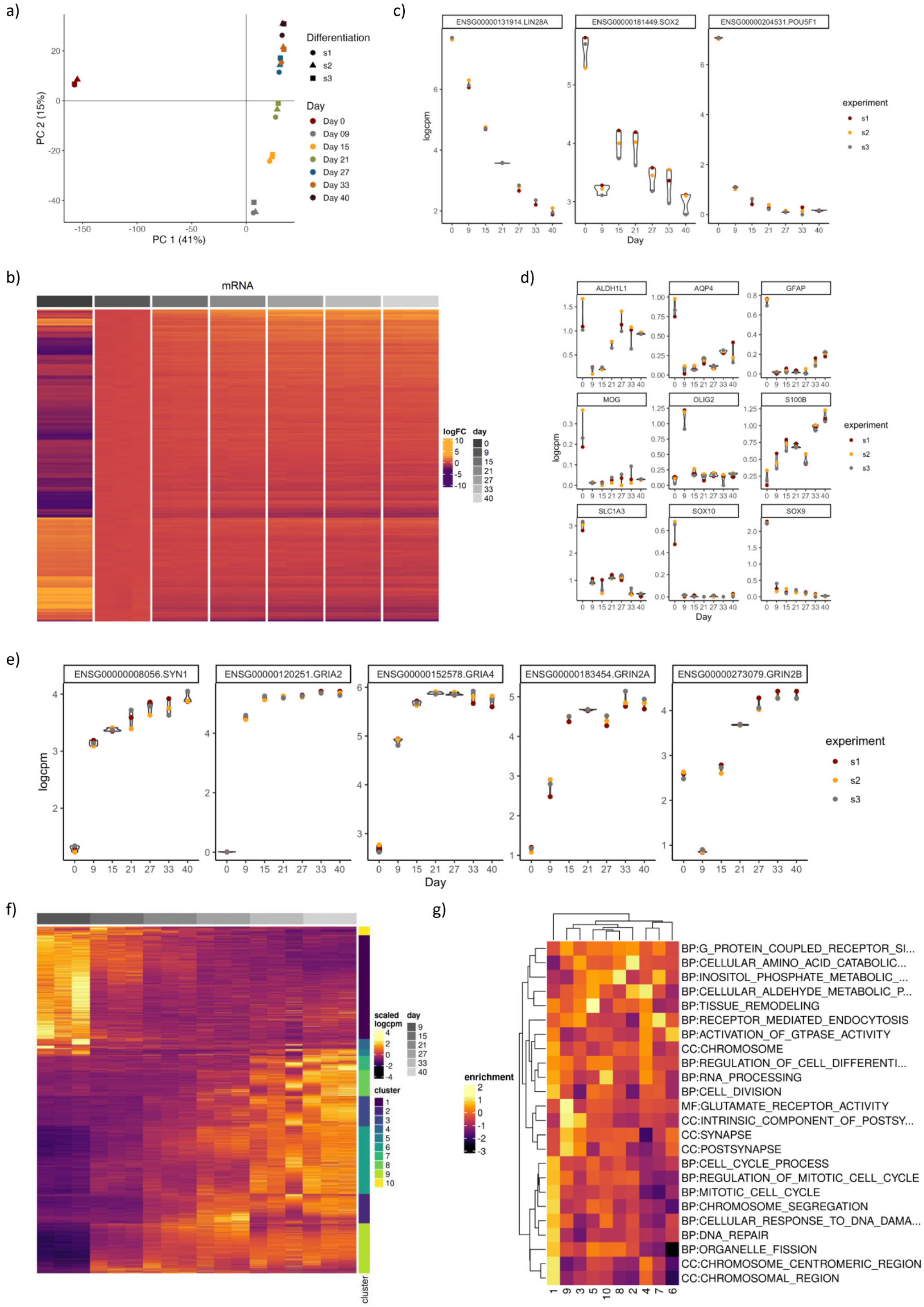

Supplementary figure S6): Supplementary information to the ribosomal depletion sequencing during human neuronal differentiation

a) PCA-plot of the ribosomal depletion (ribo-minus) sequencing with different time points separated by color and replicates indicated by shape. The ribosomal depletion sequencing was performed with three biological replicates (differentiations) per time point (n=3). b) Heatmap showing expression dynamics (log fold changes (logFC)) of mRNAs of the ribosomal depletion sequencing normalized to the first neuronal time point (day 9). Each column represents a time point and each row a gene. Shown are significantly changing genes (FDR < 0.01) with a logFC of at least 2 and log counts per million (logcpm) of at least 3. c-e) Individual example gene expression plots (logcpm) of the ribosomal depletion sequencing over the time course of differentiation. Replicates are indicated by color. Key pluripotency genes are displayed in (c), examples of glial-marker genes in (d) and examples of excitatory synaptic genes in (e). f) Heatmap displaying scaled logcpm values of significant genes (FDR < 0.01 & logFC > 2) at the neuronal time points (day 9 – day 40), clustered by partitioning around medoids (pam). g) Cluster GO-term deviation analysis (see methods) on these 10 clusters yielded a specific cluster (number 9) with an enrichment for synapse associated genes.

Supplementary Figure S7)

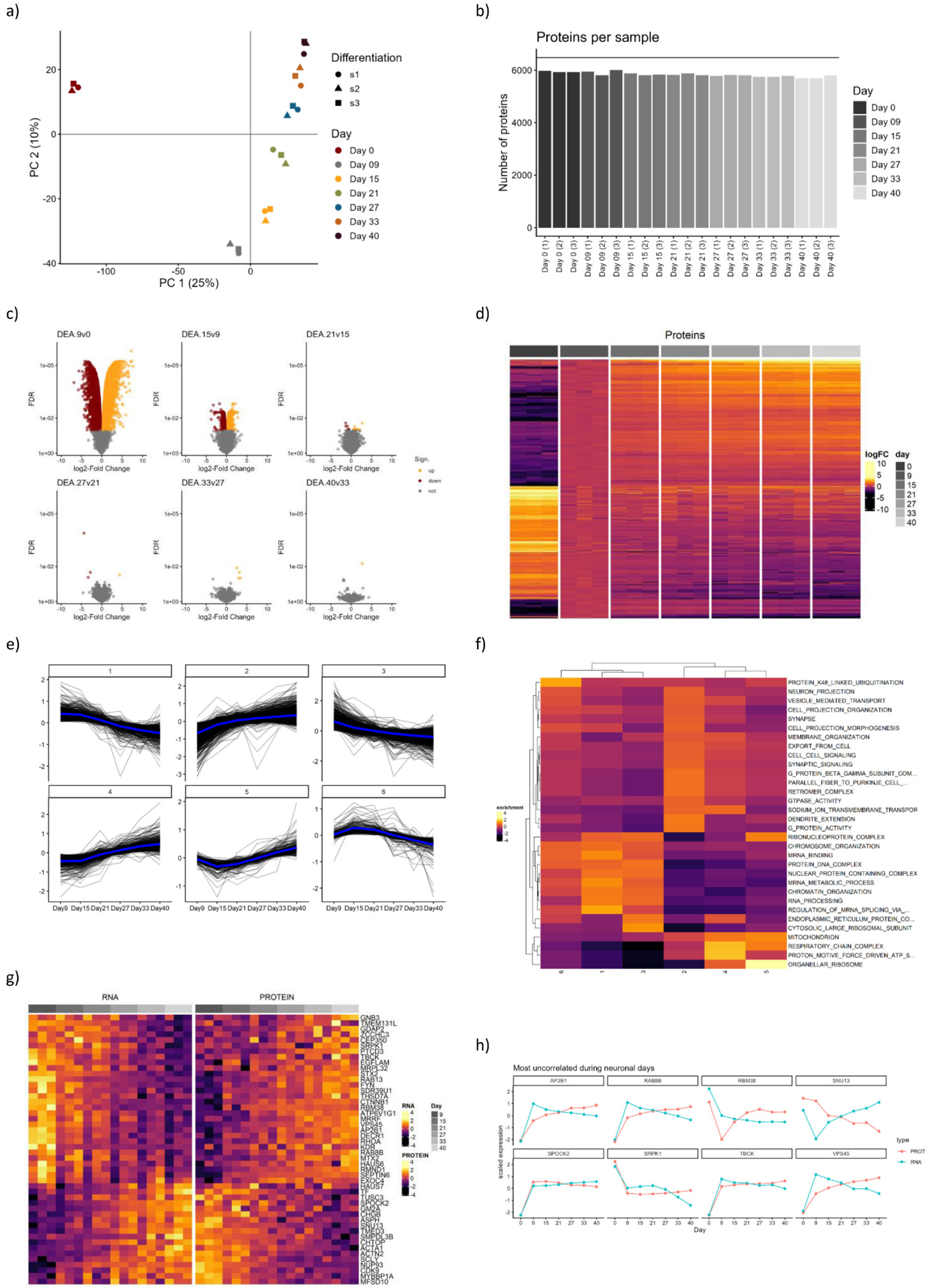

Supplementary figure S7): Supplementary information to the mass-spectrometry data during human neuronal differentiation

a) PCA-plot of the label-free mass-spectrometry (proteomics) experiment. Time points are separated by colors, replicates indicated in shapes. Proteomics was performed on three biological replicates (differentiations) per time point (n=3). b) Number of detected proteins per sample. Time points are indicated in greys; individual replicates can be identified with the last number after the "_" in the x-axis labelling. c) Volcano plots comparing neighboring time points indicate protein expression changes particularly during the first time points of the neuronal differentiation. Significantly downregulated proteins are displayed in red, significantly upregulated proteins in yellow (FDR < 0.05). d) Heatmap showing expression dynamics (logFC) normalized to the first neuronal time point (day 9) of the proteomics dataset. Each column represents a time point and each row a protein. Displayed are significantly changing proteins (FDR < 0.01) with a logFC > 2. e) Smoothed means of protein expression trajectories during the neuronal time points (day 9 – day 40) clustered by k-means. f) Cluster GO-term deviation analysis (see methods) on these 6 clusters revealed an enrichment for mitochondria associated proteins mostly in clusters 4 & 5, while synapse associated proteins are most enriched in cluster 2. g) Heatmap displaying scaled gene expression values of uncorrelated RNAs and Proteins (Pearson correlation < -0.9) of genes significantly changing during the neuronal time points (day 9 – day 40) (FDR < 0.01). h) Protein and corresponding RNA trajectories of example genes that demonstrate uncorrelated expression patterns between RNA and proteins at different neuronal time points.

Supplementary Figure S8)

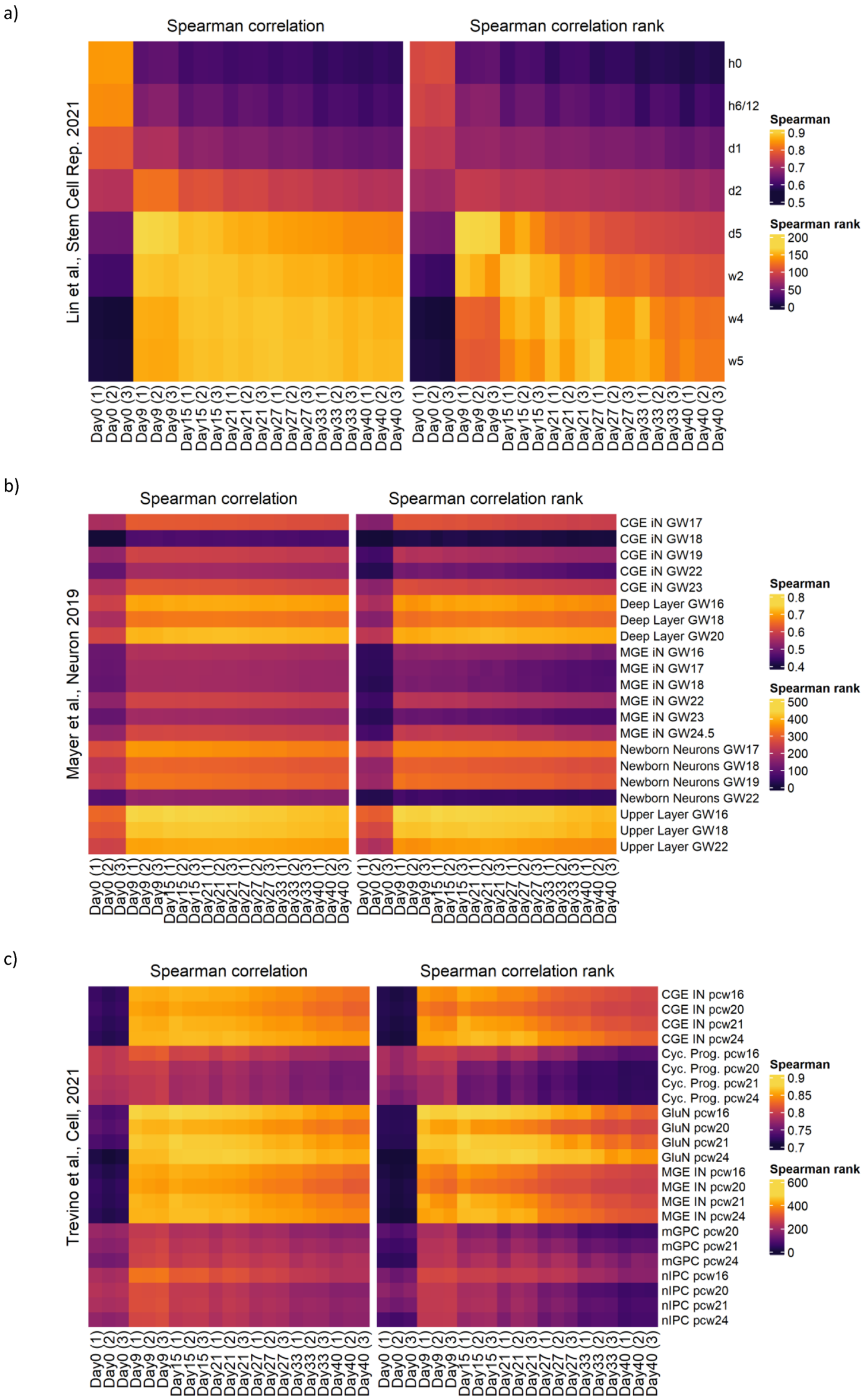

Supplementary figure S8): Staging analysis of our igNeuron RNA-expression dataset

a-c) Heatmaps indicating Spearman correlation values (left) and ranks of these Spearman correlations (right) between our RNA sequencing time course dataset and single-cell-

expression values (pseudobulk) of Ngn2-neurons cultured with astrocytes (data generated by Lin et al. (*2*)) (a) and samples obtained from the developing human cortex by Mayer et al. (*3*) (b) as well as by Trevino et al. (*4*) (c). Replicates of our time course dataset can be identified by the number in brackets following the description of the day.

Supplementary figure S9)

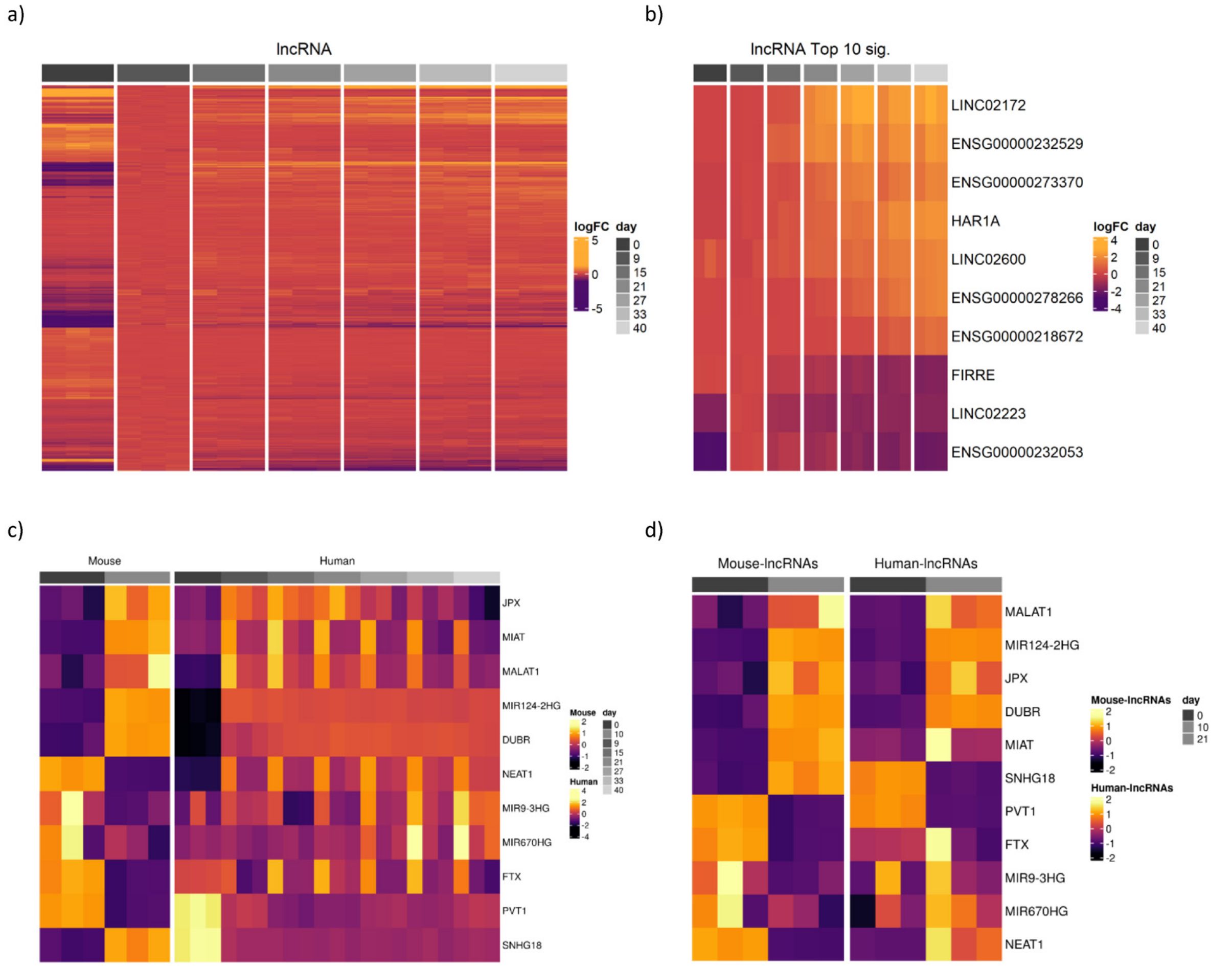

Supplementary figure S9): lncRNA expression during human neuronal differentiation

a) Heatmap indicating expression dynamics (logFC) normalized to the first neuronal time point of significantly changing lncRNAs (FDR < 0.01) over the time course of excitatory synapse formation as determined by ribosomal depletion sequencing. Each row represents a lncRNA, each column a time point. b) Expression changes (logFC) of the top ten significantly changing lncRNAs at the observed time points. c-d) Heatmaps of significantly changing lncRNAs during human neuronal differentiation (FDR < 0.05), which are also detected during mouse neuronal differentiation. Displayed are scaled logcpm-values over all time points in (c) and at two time points in (d) (mouse: day 0 and day 10, human: day 0 and day 21). Mouse data was acquired by Whipple et al. (*5*).

Supplementary figure S10)

a)

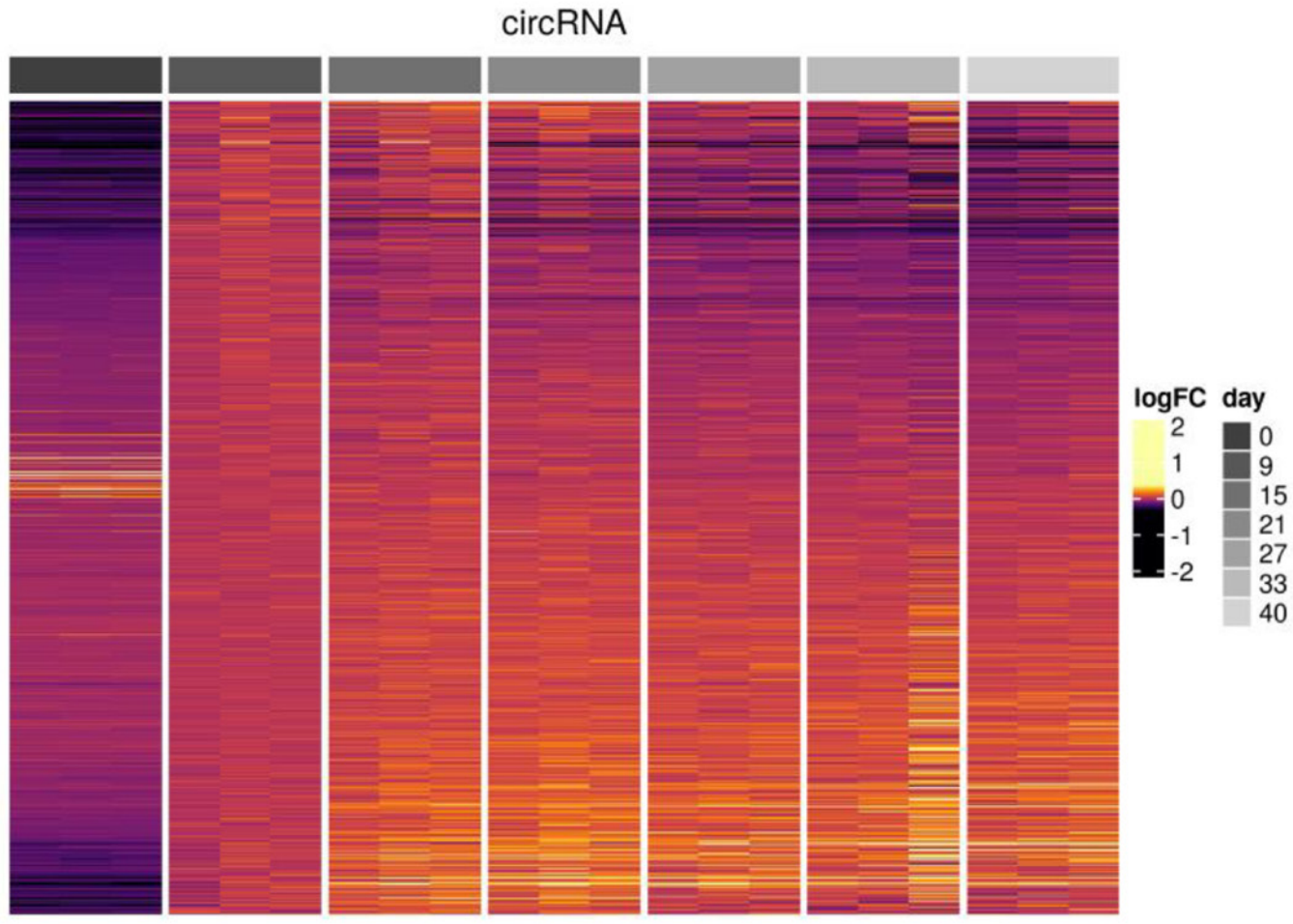

b)

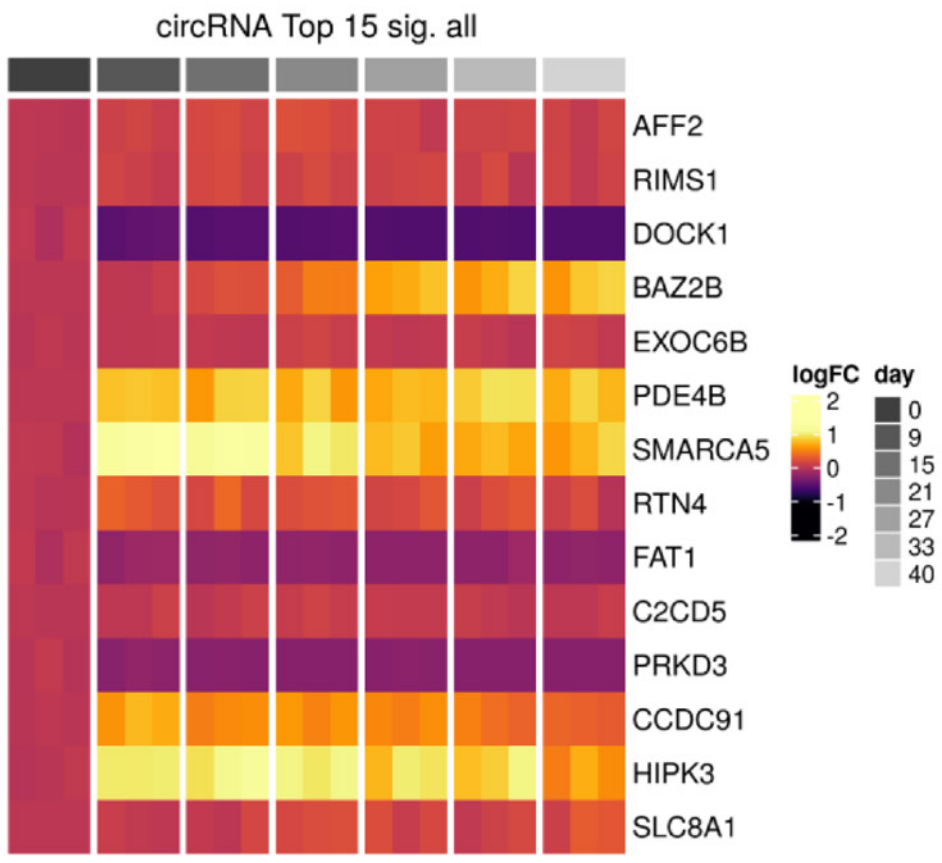

c)

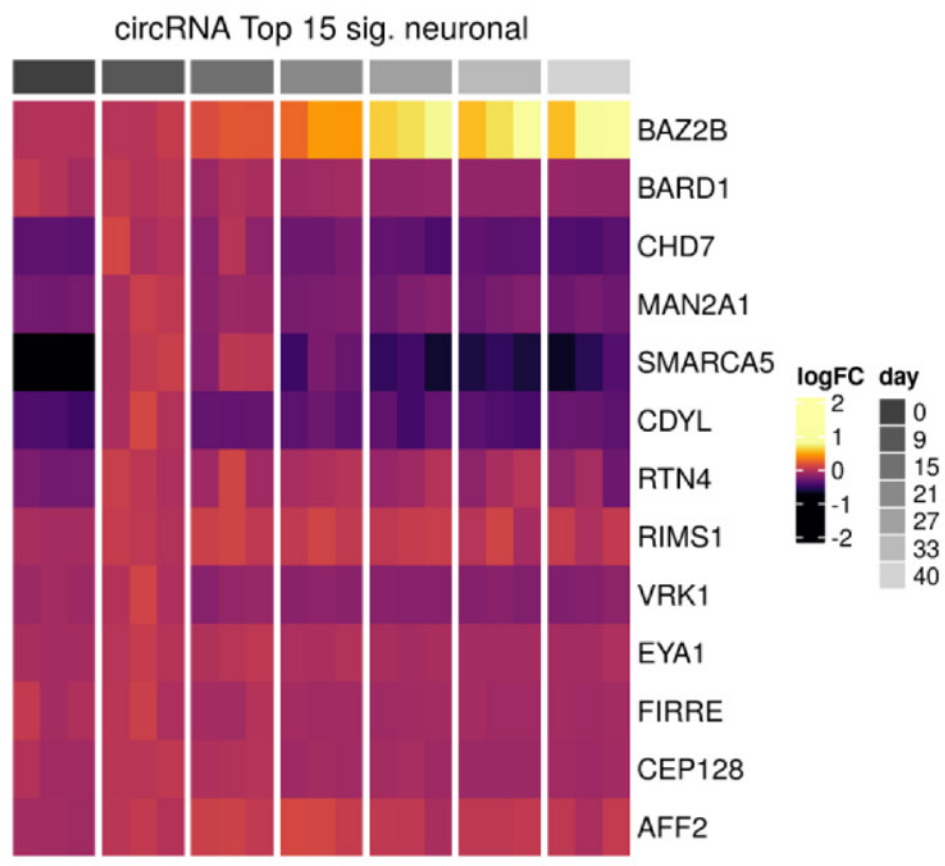

Supplementary figure S10): circRNA expression during human neuronal differentiation

a) Heatmap of significantly changing circRNAs (FDR < 0.01) over the time course of excitatory synapse development as determined by ribosomal depletion sequencing. Shown are the logFC normalized to day 9. b) Heatmap (logFC normalized to day 0) of the 15 most significantly changing circRNAs over the entire time course of neuronal differentiation. c) Heatmap (logFC normalized to day 9) displaying the 15 most significantly changing circRNAs during the neuronal time points.

Supplementary figure S11)

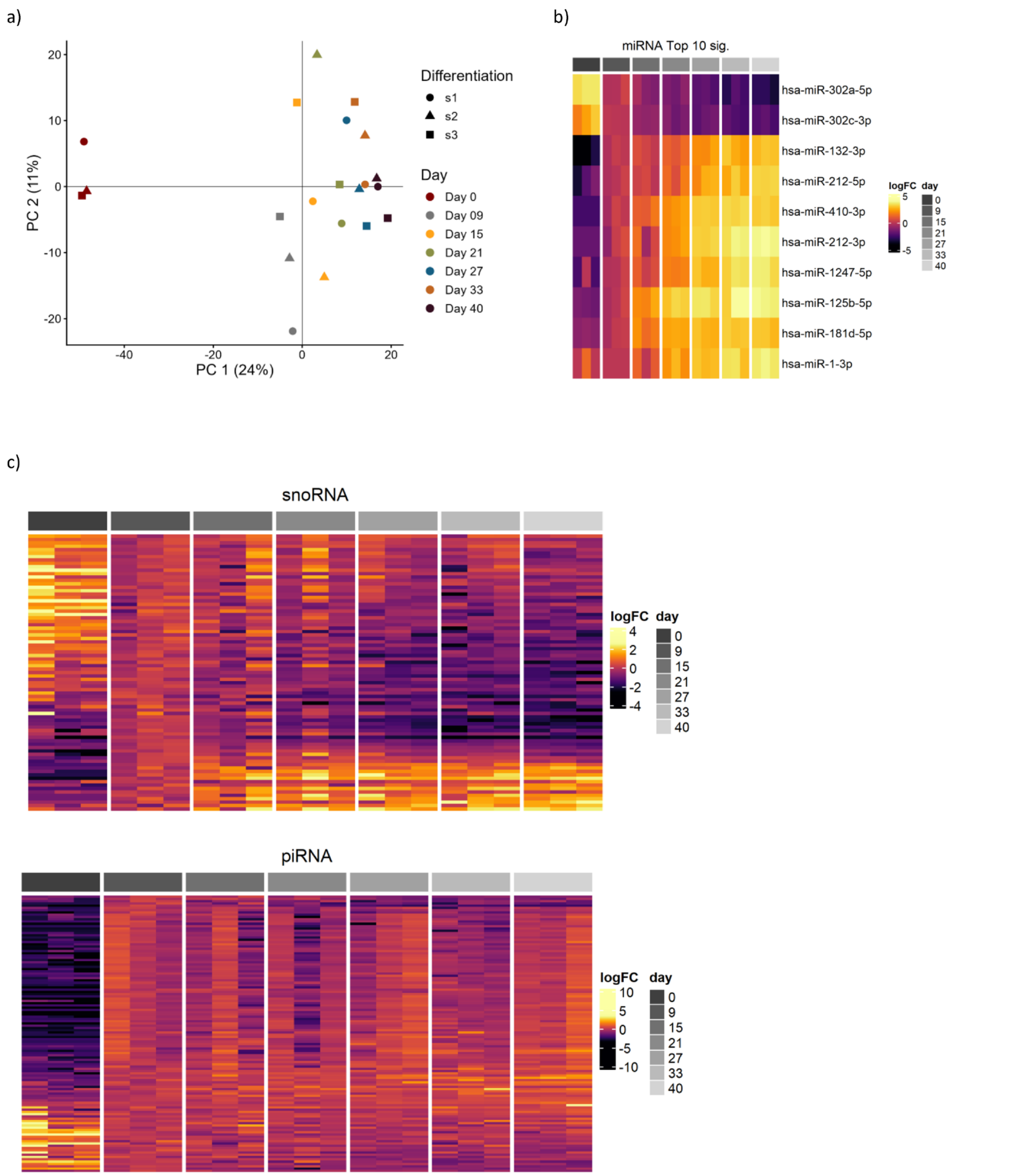

Supplementary figure S11): Supplementary information to the small RNA sequencing during human neuronal differentiation

a) PCA-plot of the small RNA sequencing. Time points are indicated by color; replicates are separated by shape. b) Heatmap displaying the logFC (normalized to day 9) of the ten most significantly changing miRNAs over the neuronal time points of human neuronal differentiation. c) Heatmaps displaying snoRNAs (upper panel) and piRNAs (lower panel) during the time course of neuronal differentiation. Shown are the logFC (normalized to day 9) for significantly changing genes (FDR < 0.05).

Supplementary figure S12)

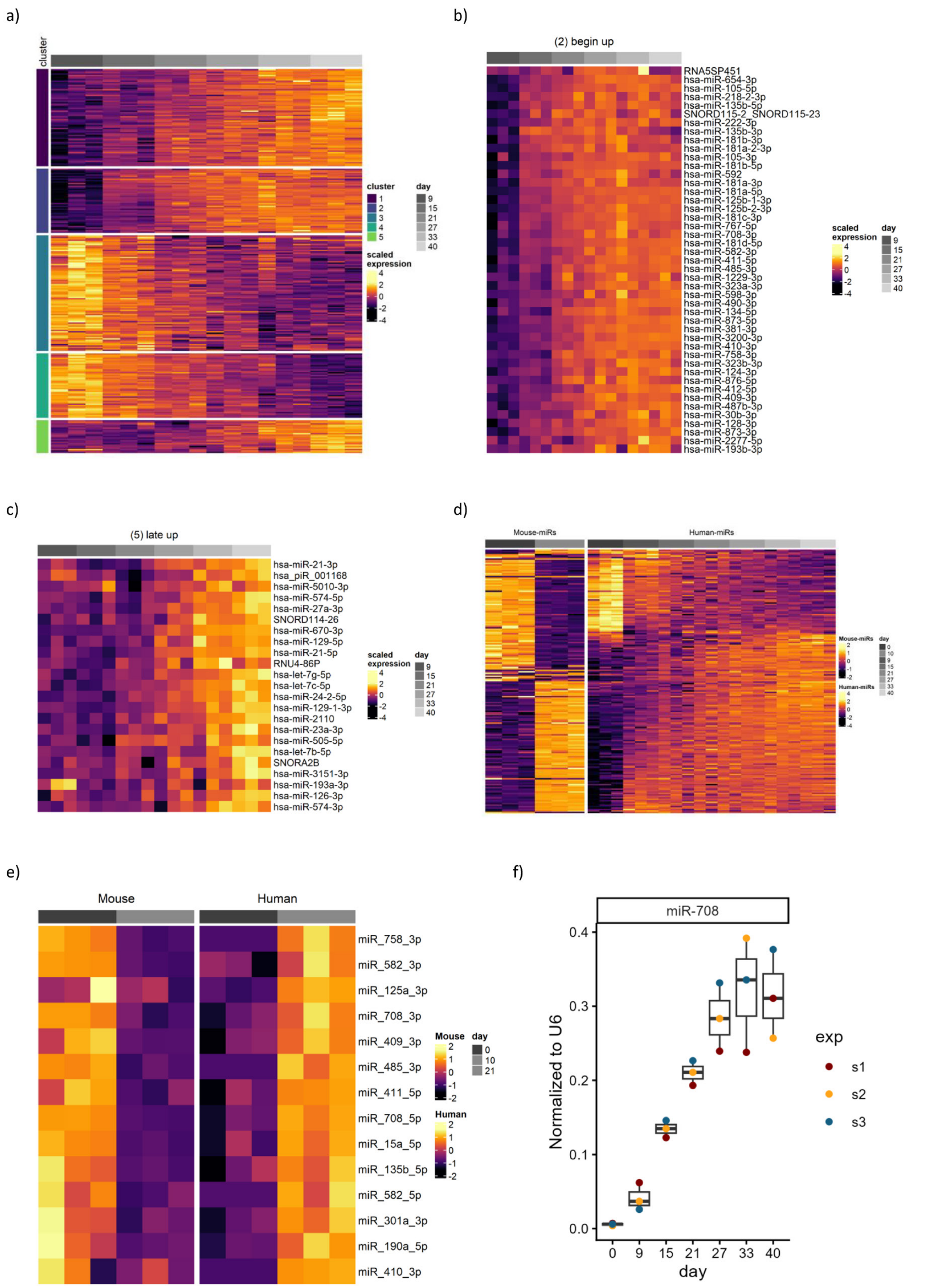

Supplementary figure S12): Clustering of the small RNA sequencing dataset and comparison to microRNAs expressed in differentiating mouse neurons

a) Heatmap displaying scaled RUVs (Remove unwanted variation from RNA sequencing data) corrected logcpm values (see methods), including a clustering of the small RNA sequencing dataset (significantly changing small RNAs with FDR < 0.05) during the neuronal time points. b-c) Heatmaps of small RNAs belonging to cluster 2 (b) and cluster 5 (c). Shown are the scaled RUVs-corrected logcpm values during the neuronal time points. d) Heatmap showing scaled logcpm-values of miRNAs commonly detected during mouse and human neuronal differentiation that change significantly over the entire time course of human neuronal differentiation. Mouse data was acquired by Whipple et al. (*5*). e) Heatmap showing the scaled logcpm values of conserved miRNAs between mouse and human that differ in their expression dynamics. Shown are days 0 and 10 (mouse) and days 0 and 21 (human) f) TaqMan miRNA qPCR for miR-708-5p on the time course samples used for the small RNA sequencing (Fig 2e, g).

Supplementary figure S13)

a)

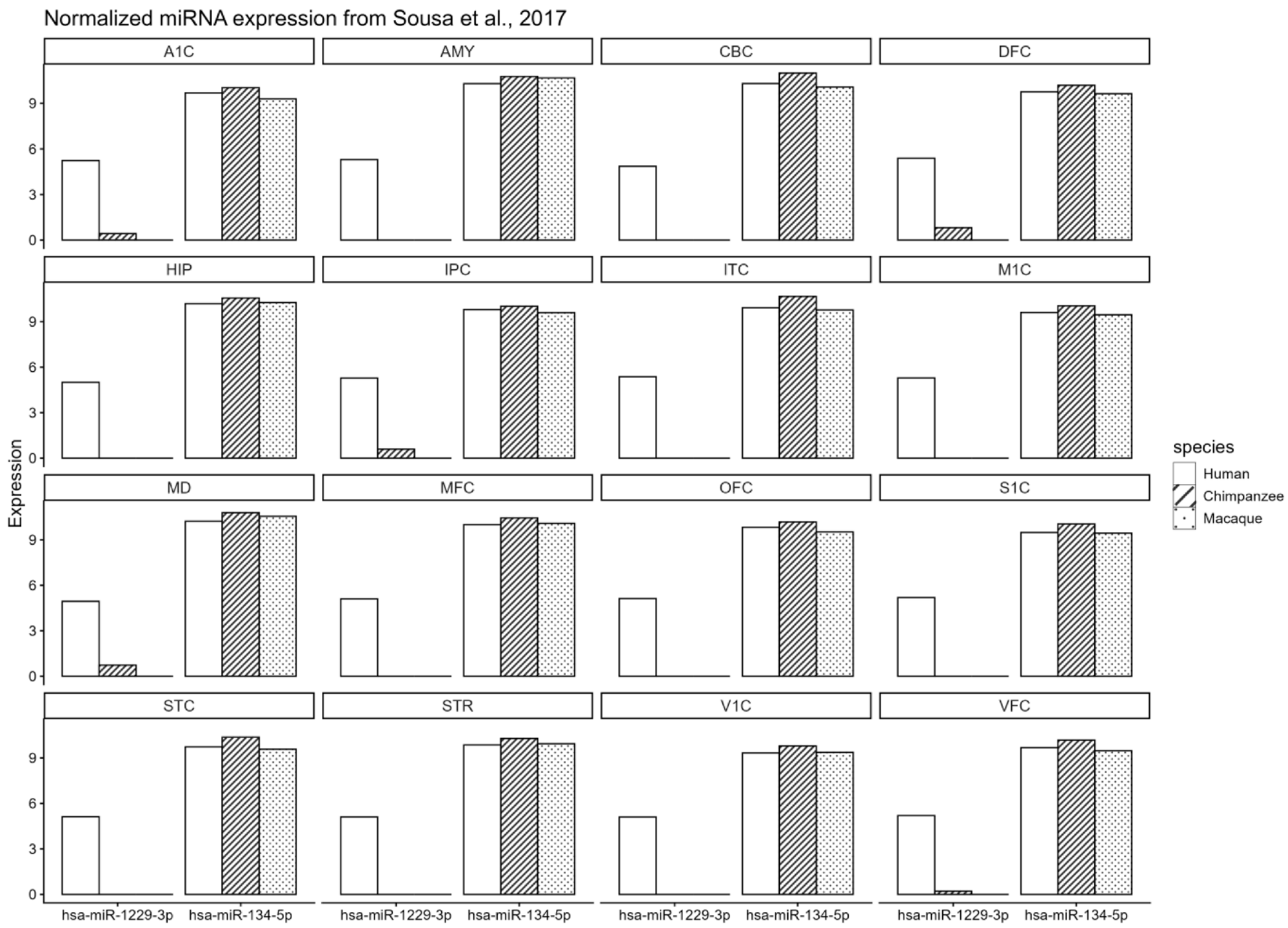

Supplementary figure S13): miR-1229-3p expression in human post-mortem brain samples
Normalized expression of miR-134-5p and miR-1229-3p in 16 brain areas of human, chimpanzee and macaque. The original data was obtained by Sousa et al. (*6*). For more information regarding methodology and abbreviations we refer to their publication and supplement.

Supplementary figure S14)

a)

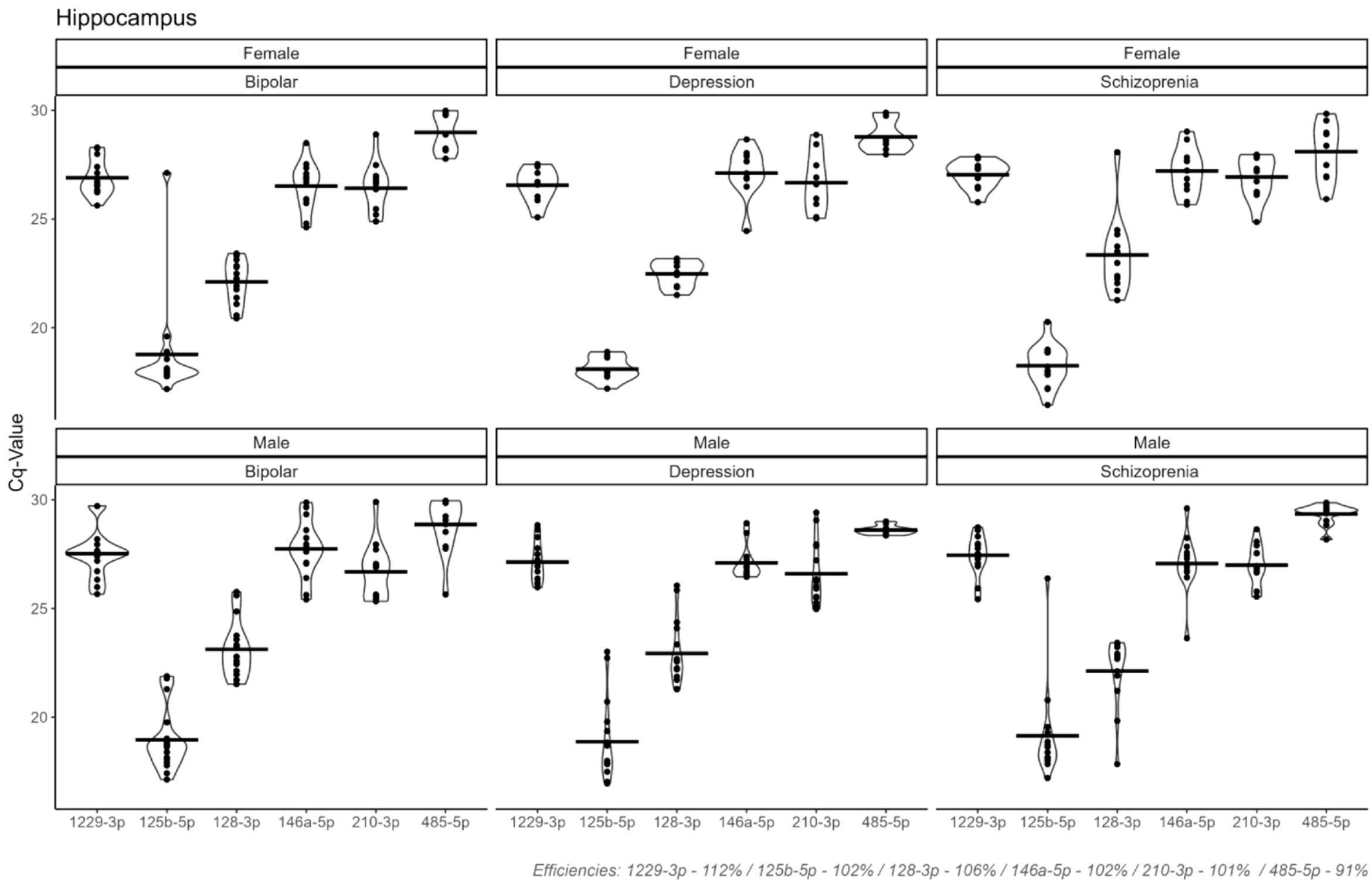

b)

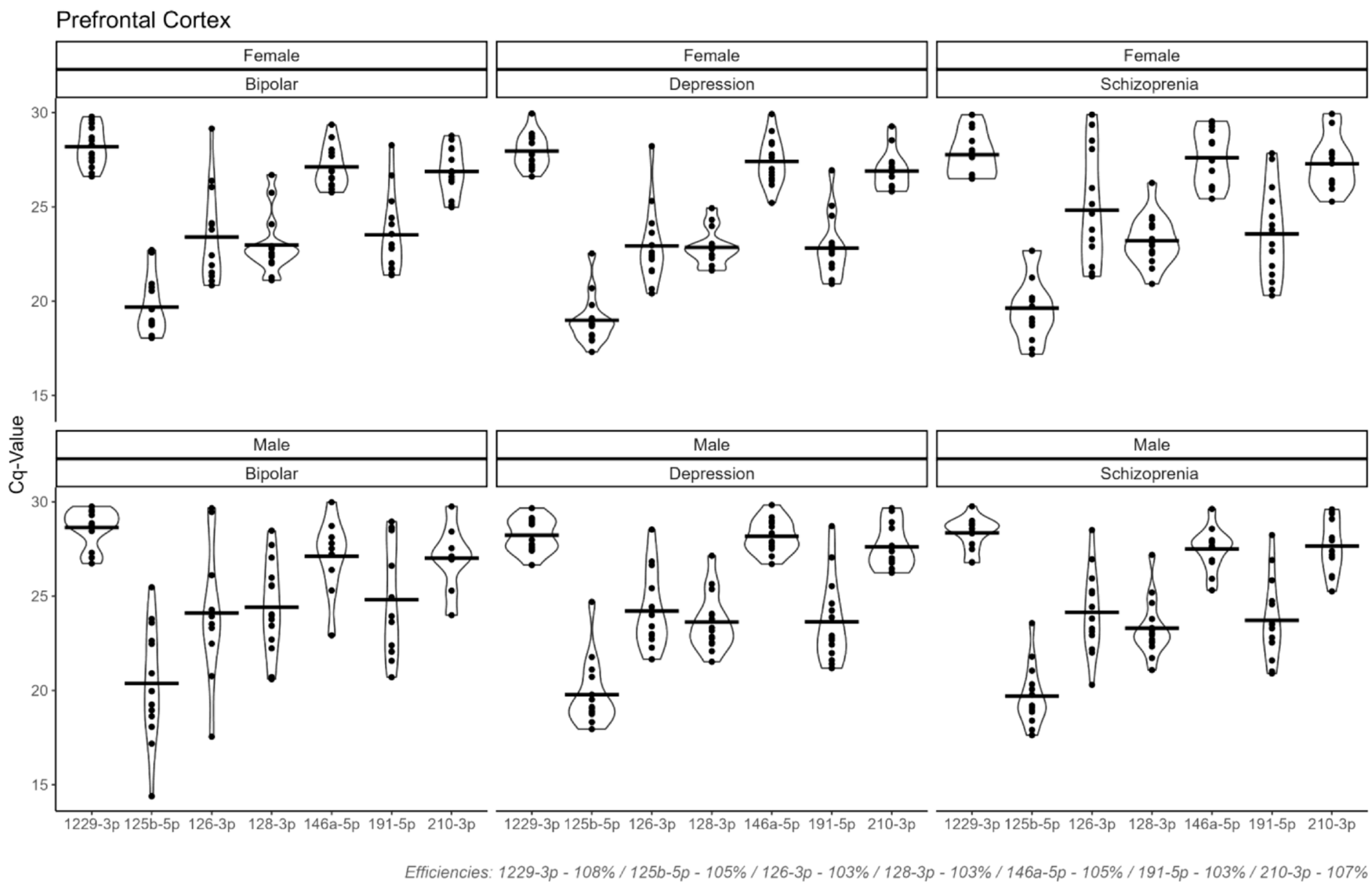

Supplementary figure S14): Expression of selected microRNAs in post-mortem brain samples of Danish psychiatry patients

Expression (Cq-values) of selected miRNAs in two regions of the human brain (as determined by qPCR on post-mortem samples of a Danish cohort of psychiatry patients). In the

hippocampus (a), miR-1229-3p expression was measured together with the expression of miR-125b-5p, miR-128-3p, miR-146a-5p, miR-210-3p, miR-485-5p in female and male patients diagnosed with bipolar disorder (n = 14 & n = 17), depression (n = 9 & n = 16) as well as schizophrenia (n = 14 & n = 14). In the prefrontal cortex (b), miR-1229-3p was measured together with miR-125b-5p, miR-126-3p, miR-128-3p, miR-146a-5p, miR-191-5p and miR-210-3p (bipolar disorder: n = 14 (f) & n = 14 (m); depression: n = 14 (f) & n = 14 (m); schizophrenia: n = 14 (f) & n = 14 (m)). Cq-values are inversely correlated with the expression, meaning a low Cq-value indicates a high expression of the respective miRNA. The amplification efficiencies of each miRNA primer pair are indicated on the bottom of the plots.

Supplementary figure S15)

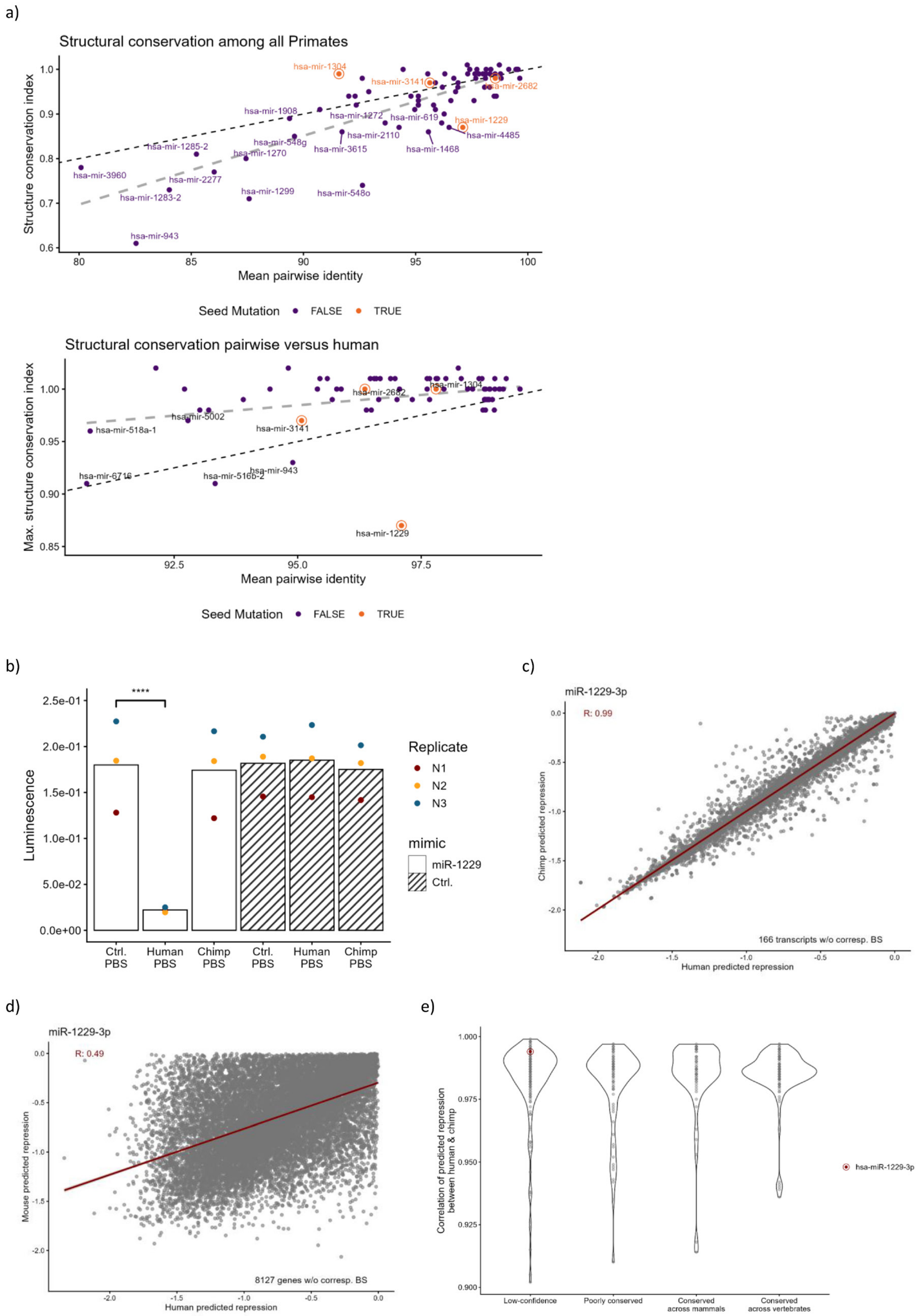

Supplementary figure S15): microRNA and miRNA-target conservation analysis

a) Structural conservation analysis of expressed human miRNA precursors with at least one mismatch identified in analyzed species across primates (excluding marmoset). Plotted is the

mean identity of the contrasted precursor sequences on the x-axis and the structural conservation index (SCI) generated by RNAz on the y-axis. The grey dashed line indicates the linear regression of all analyzed precursors. The black reference line (slope = 0.01 and intercept = 0) roughly marks a threshold below which miRNA precursors can be regarded as not structurally conserved. The upper panel indicates the structural conservation among all primates, the lower panel the structural conservation of the primate sequences pairwise against humans (in this panel, the maximum SCI of these comparisons is plotted). miR-1229 is particularly outstanding in the lower plot since it is specifically mutated in humans but well conserved among the other analyzed primates. b) Luciferase reporter assay in HEK293 cells transfected with a miR-1229-3p perfect-binding site (PBS) reporter together with synthetic miRNA mimics (miR-1229, control). (Statistics: linear model: ~ condition + replicate, post-hoc analysis with emmeans, n = 3, **** = p-value < 0.0001). c-d) Target conservation analysis of mir-1229-3p. c) Human 3'UTRs were lifted over to the chimpanzee genome and scanned for binding sites for miR-1229-3p. Plotted are the scanMiR repression scores for each human and chimp transcript for miR-1229-3p. The pearson correlation estimate is annotated in red. d) Human and Mouse 3'UTRs (Ensembl Annotation 107) were scanned with scanMiR for hsa-miR-1229-3p binding sites. The most repressed transcript per gene was kept and the results subsequently merged by gene name. Plotted are the hsa-miR-1229-3p scanMiR predicted repression scores for these human and corresponding mouse transcripts. The pearson correlation estimate is annotated in red. e) Distribution of the correlation of predicted repression across human and chimpanzee for each miRNA detected in the small RNA sequencing during the time course of neuronal differentiation. We considered those miRNAs, which are annotated correspondingly in scanMiR including conservation information. Plotted are the Pearson correlation values for all those analyzed miRNAs splitted by TargetScan (*7*) conservation indications.

Supplementary figure S16)

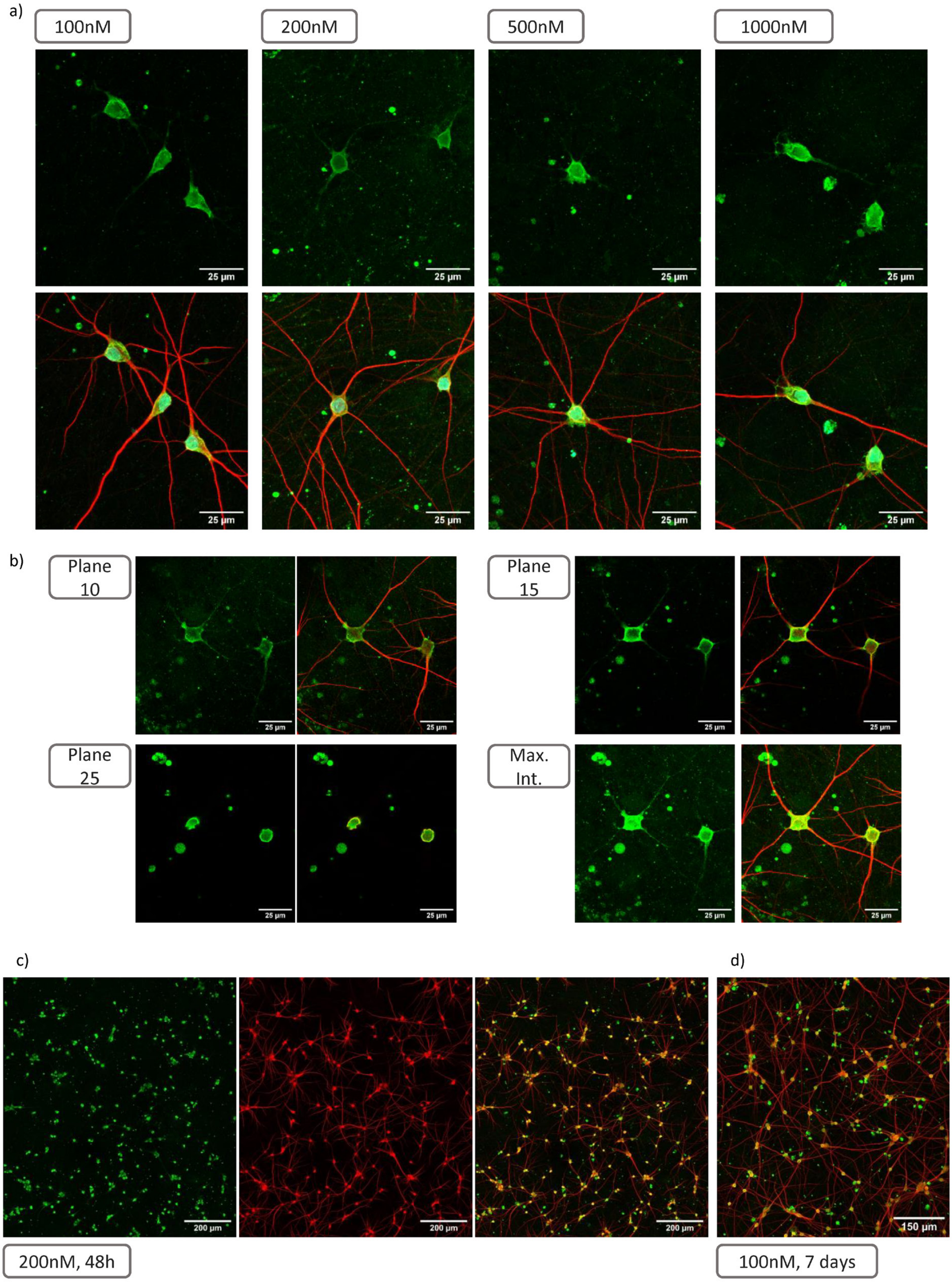

Supplementary figure S16): Optimization of pLNA application in human neurons

a) Representative images of igNeurons (day 29) that were treated with a fluorescently labeled control pLNA at different concentrations. The pLNAs were added to the medium without

transfection 48h before fixation. pLNAs are displayed in green (upper panels), the merge with MAP2 (in red) and Hoechst (blue) is shown in the lower panels. b) Different z-stack planes illustrate the presence of fluorescently labeled pLNA (green) inside cells (MAP2 shown in red). 200nM pLNA was given 48h before fixation. c) An example tile image (20x) demonstrates a near 100% uptake efficiency of the pLNAs at a concentration of 200nM. Fixation was performed 48h following pLNA application. The pLNA is displayed in green, MAP2 in red. d) Imaging of igNeurons at day 35 (seven days after pLNA treatment) illustrates the stability of the applied pLNAs. The pLNA is displayed in green, MAP2 in red.

Supplementary figure S17)

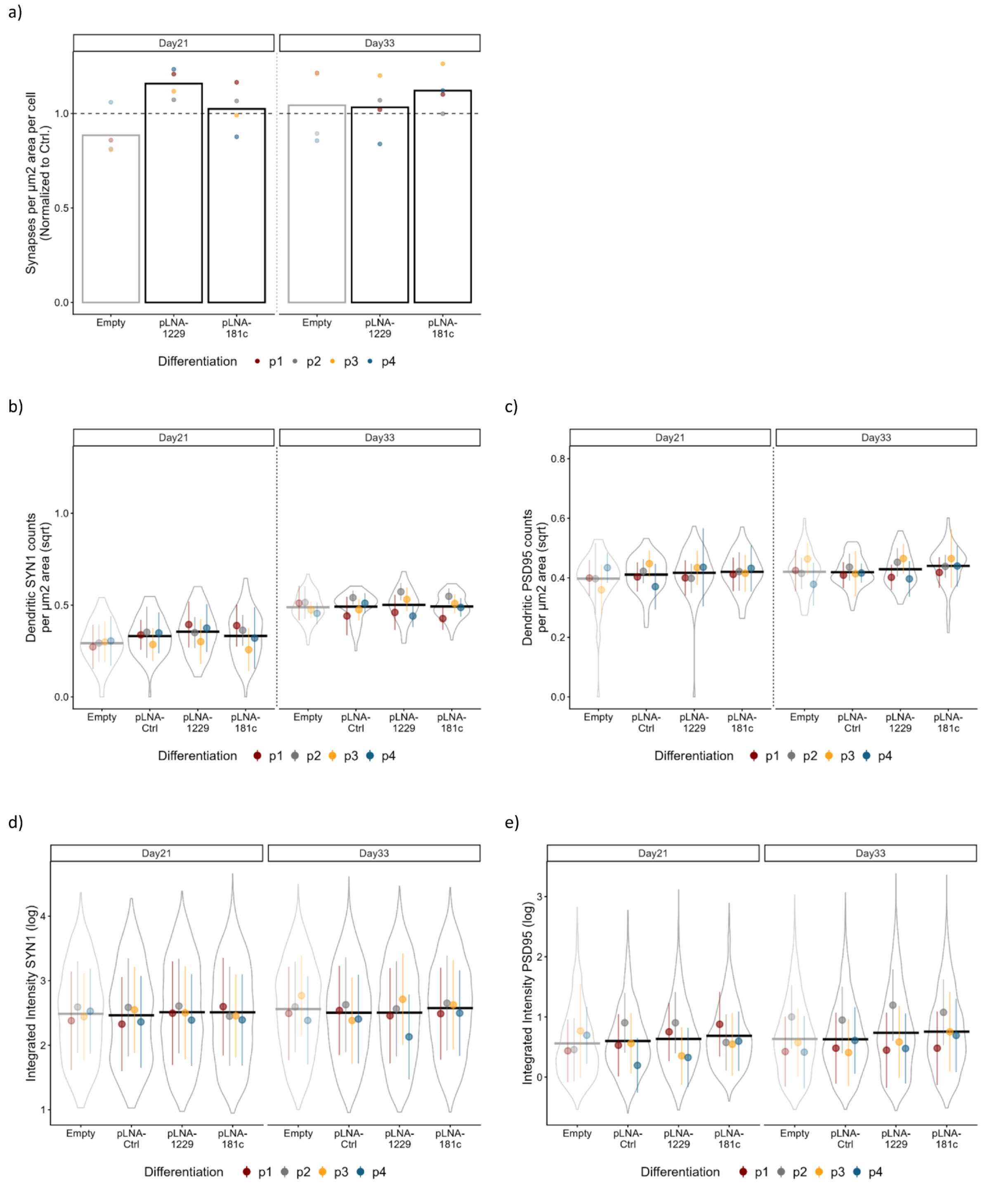

Supplementary figure S17): Quantification of synaptic markers in pLNA-treated human neurons

a) Normalized values (the mean of each differentiation was divided by the mean of the pLNA-Ctrl condition of the respective differentiation) of the synapse co-cluster analysis upon pLNA treatment (shown in Fig. 3e). b-c) Quantification of the SYN1 (b) and PSD95 (c) puncta density in dendrites, normalized to the dendritic area. Values were transformed with the square root to account for skewed data. A robust linear model was applied over the aggregated means of each

pLNA-condition and differentiation at each of the time points (~ condition + differentiation). Post-hoc analysis was conducted using emmeans (n = 4). d-e) Quantification of the integrated intensity values of SYN1 (d) and PSD95 (e) puncta detected in dendrites. Values were log-transformed to account for skewed data (n = 4).

Supplementary figure S18)

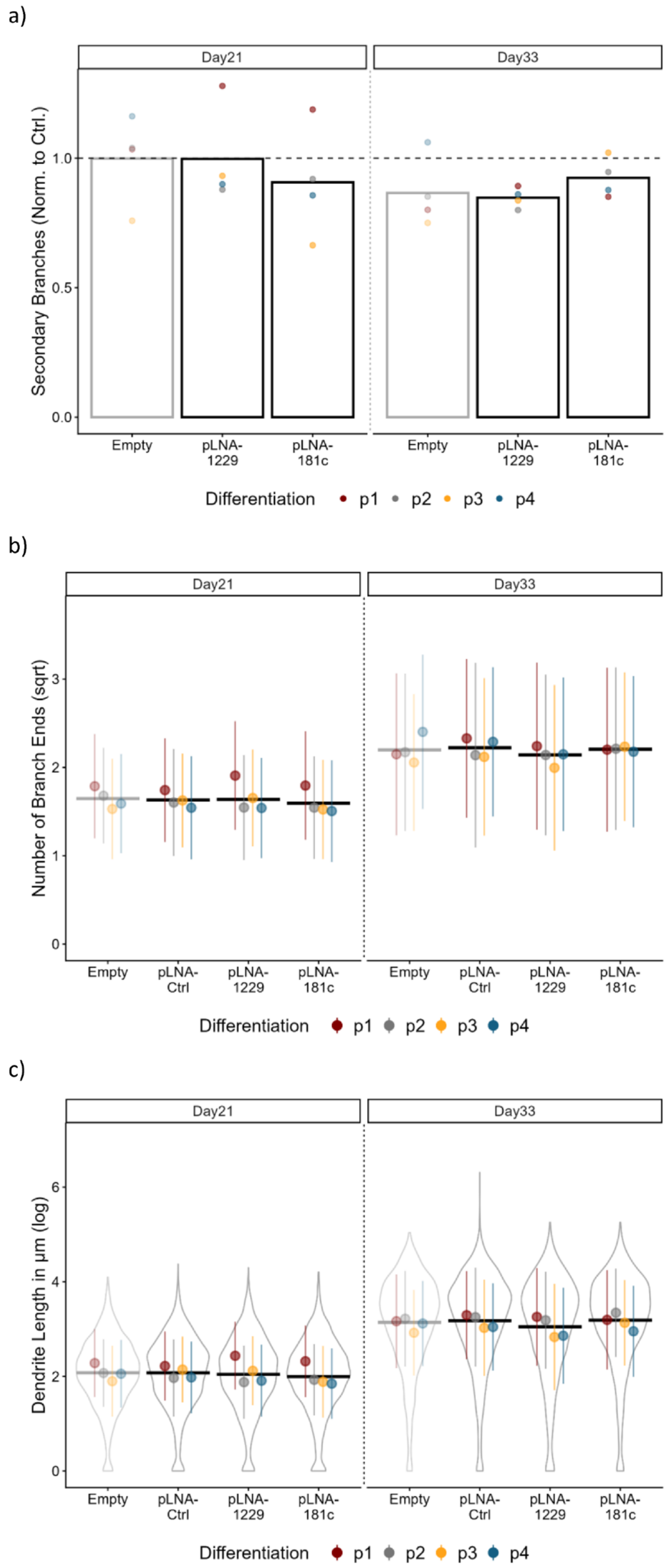

Supplementary figure S18): Quantification of dendritic complexity in pLNA-treated neurons
a) Normalized values (the mean of each differentiation was divided by the mean of the pLNA-Ctrl condition of the respective differentiation) of the dendritogenesis analysis (number of secondary branches) upon pLNA treatment (shown in Fig. 3f). b-c) Quantification of the number of branch ends (b) and dendritic length (c) per cell. Values were log-transformed to account for skewed data. A robust linear model was applied over the aggregated means of each

pLNA-condition and differentiation at each of the time points (~ condition + differentiation). Post-hoc analysis was done using emmeans (n = 4).

Supplementary figure S19)

a)

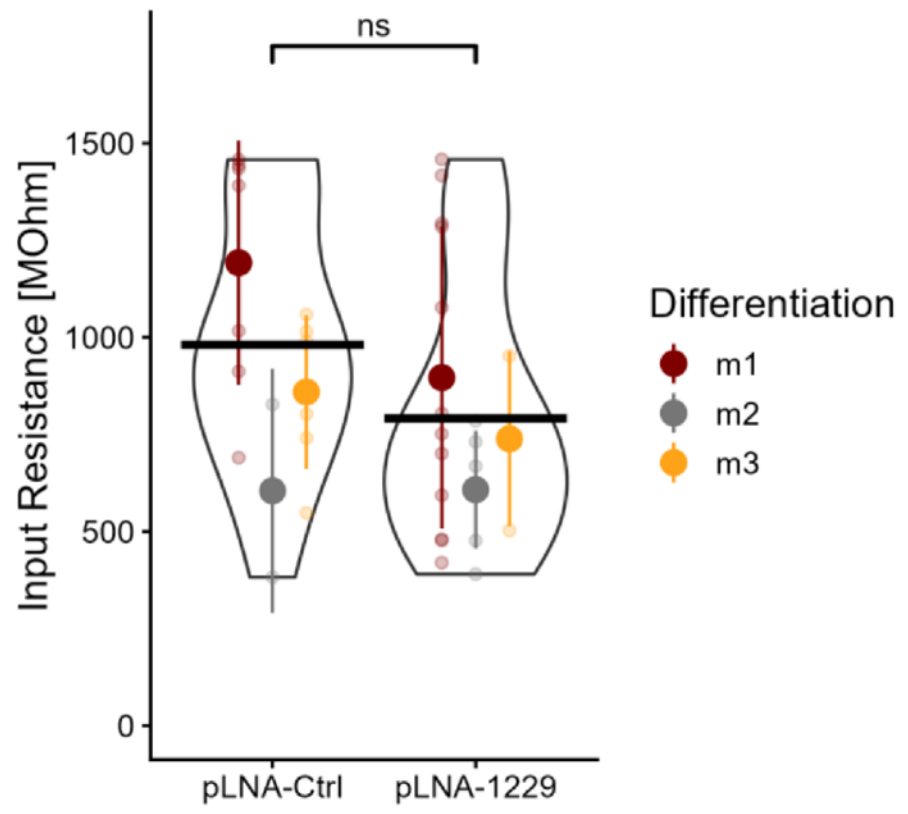

b)

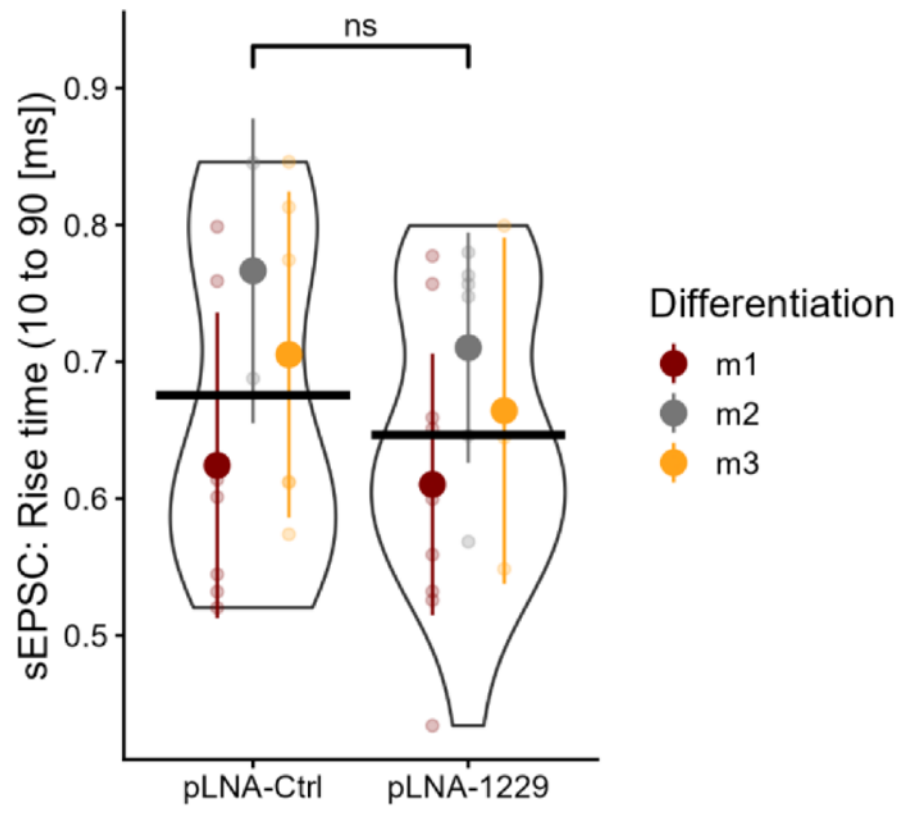

c)

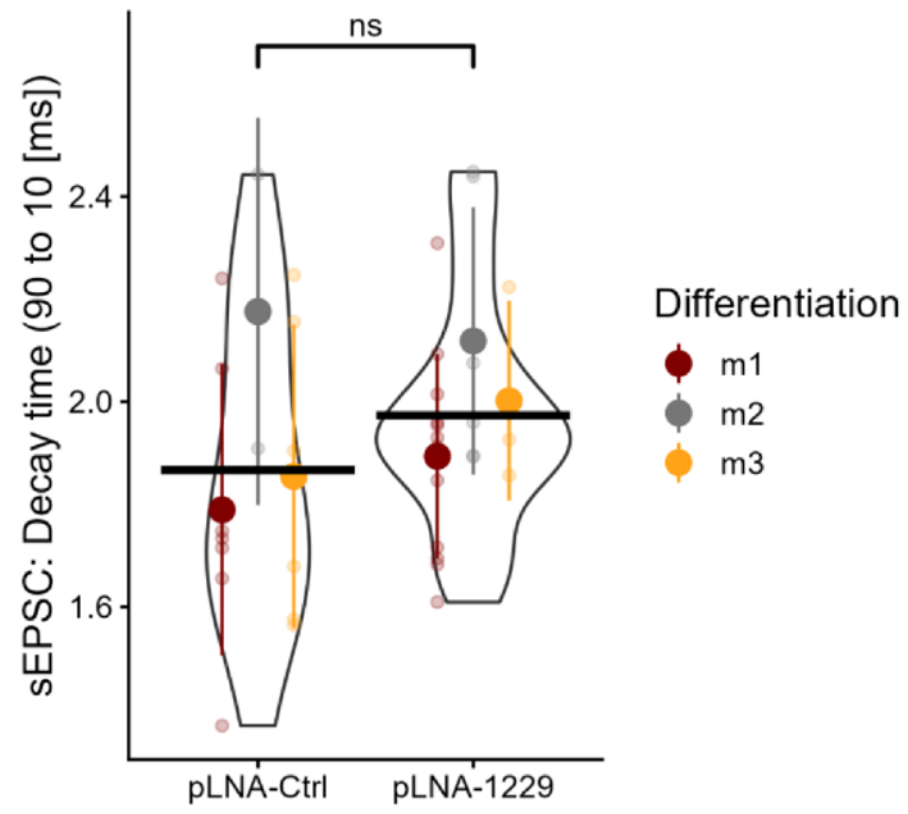

d)

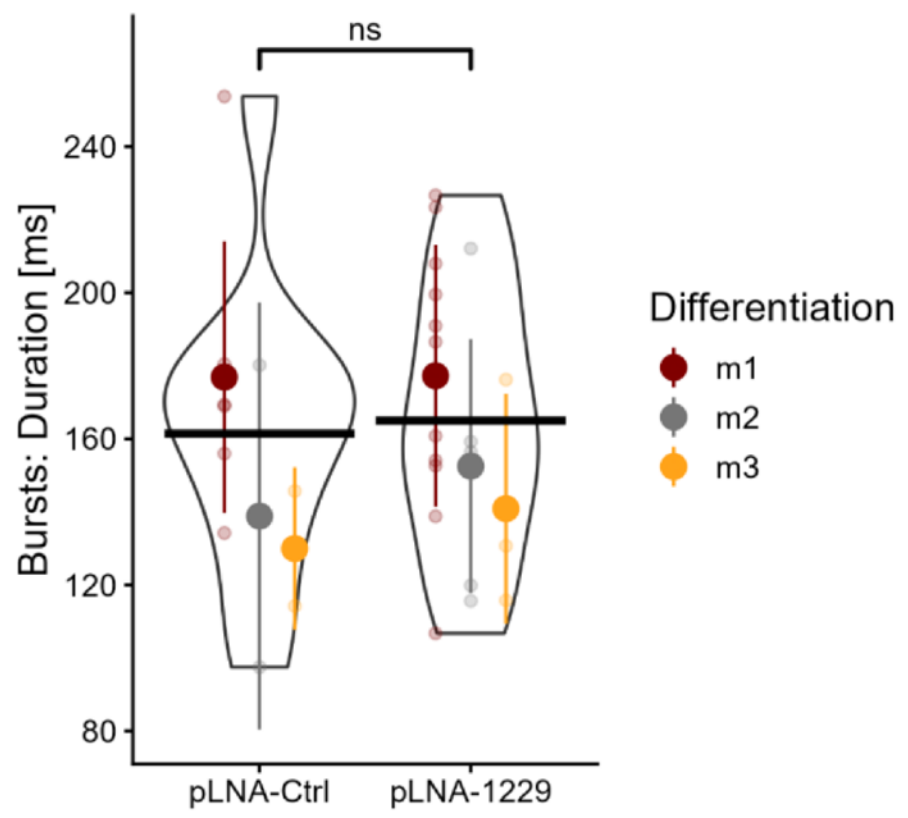

Supplementary Figure S19): Additional information on electrophysiological recordings in miR-1229-3p depleted neurons

a-d) Shown are the input resistance (a), the sEPSC rise time (b), the sEPSC decay time (c) and the duration of detected large-amplitude bursts (d) as determined by patch-clamp recording in igNeurons (day 40-42) upon pLNA treatment. Values were mean-aggregated per cell (there was only one value for the input resistance per cell) and a linear model plus emmeans was used on

these values for the statistical analyses (~ condition + differentiation, n = 15/21 for the Input Resistance, Rise time & Decay time and n = 11/21 for the Burst duration).

Supplementary figure S20)

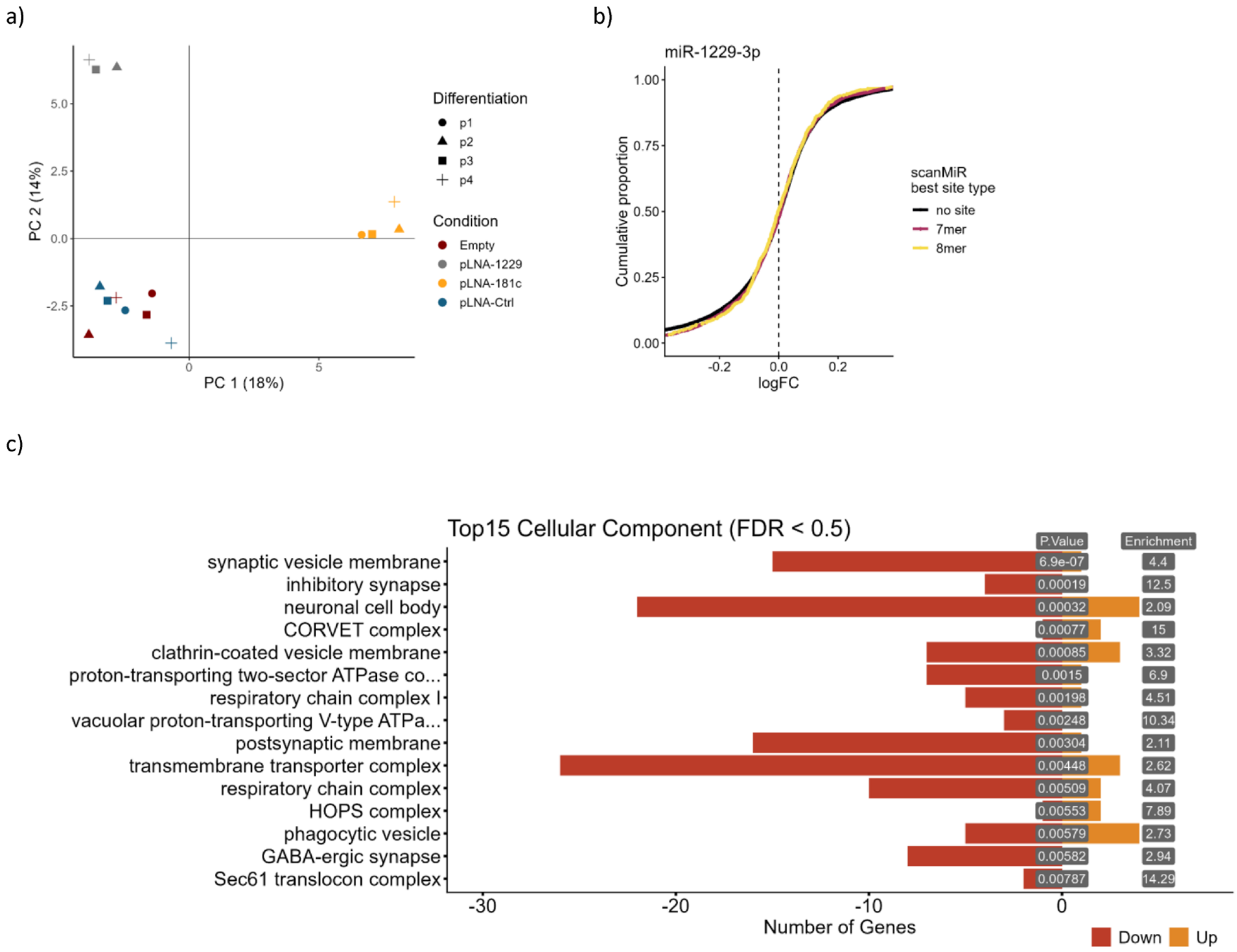

Supplementary figure S20): Supplementary information on the RNA sequencing in pLNA-treated human neurons

a) PCA-plot of the polyA-sequencing performed following pLNA-treatment. Conditions are separated by color, replicates are indicated by shape. The pLNA-Ctrl condition and the untreated (Empty) condition cluster well together, arguing against general non-specific effects caused by the pLNA treatment. b) Cumulative distribution plot (CD-Plot) of predicted miR-1229-3p targets generated with EnrichMiR (*8*). Shown is the relative up- or downregulation of genes as determined in the pLNA-sequencing depending on predicted miR-1229-3p binding sites in their UTR. There is no clear shift visible between the genes with predicted 7mer or 8mer sites and the genes without predicted binding site. c) GO-Term analysis (cellular component) of significantly changing genes (FDR < 0.5) in the pLNA-1229 condition. Displayed are the top 15 significant GO-Terms with less than 500 genes annotated. The number of significantly changing genes within each GO-term is represented by the red (downregulated) and yellow (upregulated) bars.

Supplementary figure S21)

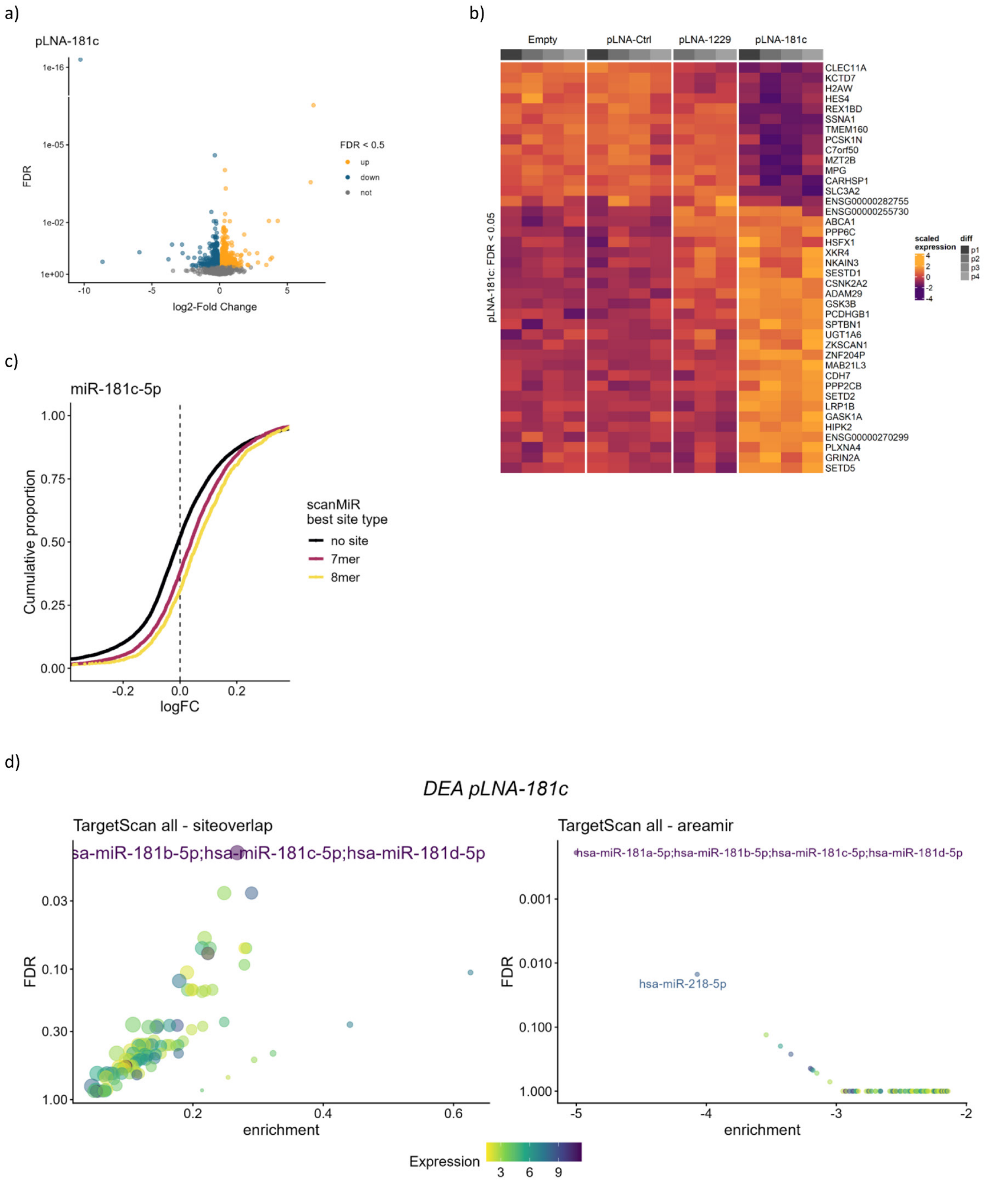

Supplementary figure S21): Differential expression and enrichMiR analyses on the pLNA RNA sequencing dataset for miR-181c-5p

a) Volcano plot indicating significantly changing genes upon miR-181c-5p depletion. Candidate up- and downregulated genes (FDR < 0.5) are displayed in yellow and blue, respectively. b) Heatmap of significantly changing genes (FDR < 0.05) in the pLNA-181c condition. Shown is the scaled expression across conditions. There were 40 genes changing with an FDR < 0.05 and 1323 genes changing with an FDR < 0.5. c) Cumulative distribution plot split by site-type indicates the expected upregulation of miR-181c-5p targets upon miR-181c-5p depletion. d) Target enrichment plots generated with enrichMiR demonstrate

significant enrichment of miR-181c-5p binding sites among upregulated genes in the pLNA-181c condition (compared to the pLNA-Ctrl). The TargetScan database (all 7mer and 8mer sites) was used in conjunction with the siteoverlap test (left panel, including genes with FDR < 0.5) and the areamir test (right panel). The log2-fold enrichment (siteoverlap test) or a normalized enrichment score (areamir test) is displayed on the x-axes and the FDR values of the statistical tests are shown on the y-axes. The miRNA expression at day 21 of the time course experiment is indicated by color. Please refer to the enrichMiR publication for details upon statistical tests and databases (*8*).

Supplementary figure S22)

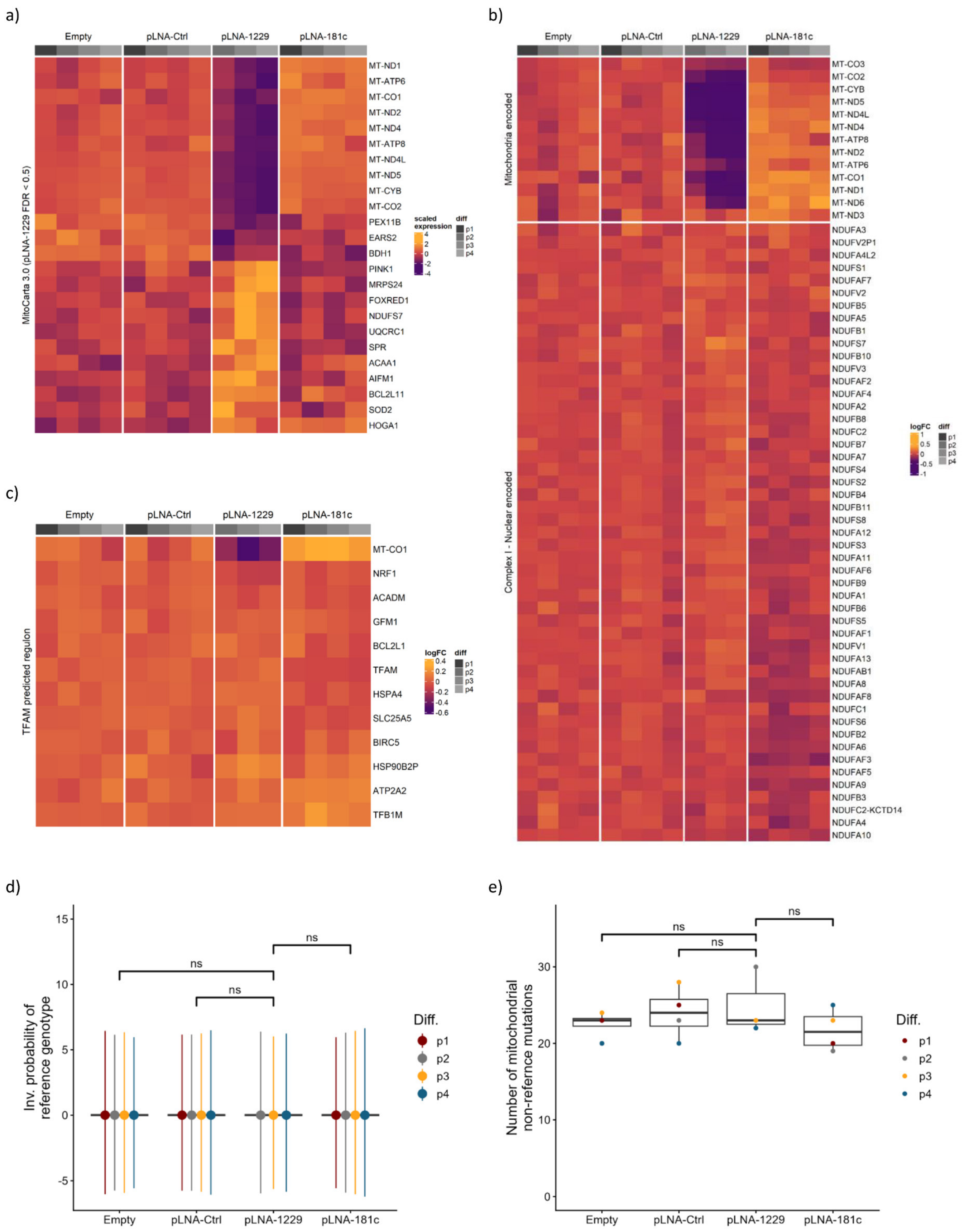

Supplementary figure S22): Supplementary analyses of the pLNA RNA sequencing dataset with regards to mitochondria

a) Heatmap of candidate changing genes upon pLNA-1229 treatment (FDR < 0.5), intersected with the MitoCarta 3.0 dataset (*9*). Shown is the scaled gene expression. b) Heatmap showing logFC of mitochondrial encoded genes (“MT-“; upper panel) and nuclear encoded mitochondrial complex I genes (“NDU"; lower panel) across pLNA conditions in comparison

to the pLNA-Ctrl as measured by RNA sequencing. c) Heatmap displaying gene expression changes of predicted TFAM target genes upon pLNA-treatment in comparison to the pLNA-Ctrl. TFAM predictions were obtained from Müller-Dott et al. (*10*). d-e) A single nucleotide polymorphism (SNP) analysis was performed on transcripts encoded by the mitochondrial DNA. (d) Inverse probability of each condition and analyzed replicate to be associated with the reference genotype (based on the number of detected SNPs in genes encoded by the mitochondrial genome). A low value indicates a high confidence in these plots. (e) Total number of mitochondrial SNPs in comparison to the reference genome across conditions. A linear model plus emmeans (x ~ Condition + Differentiation) yielded no significant difference in the number of mitochondrial SNPs across conditions (with allele frequency over 1/1000).

Supplementary figure S23)

a)

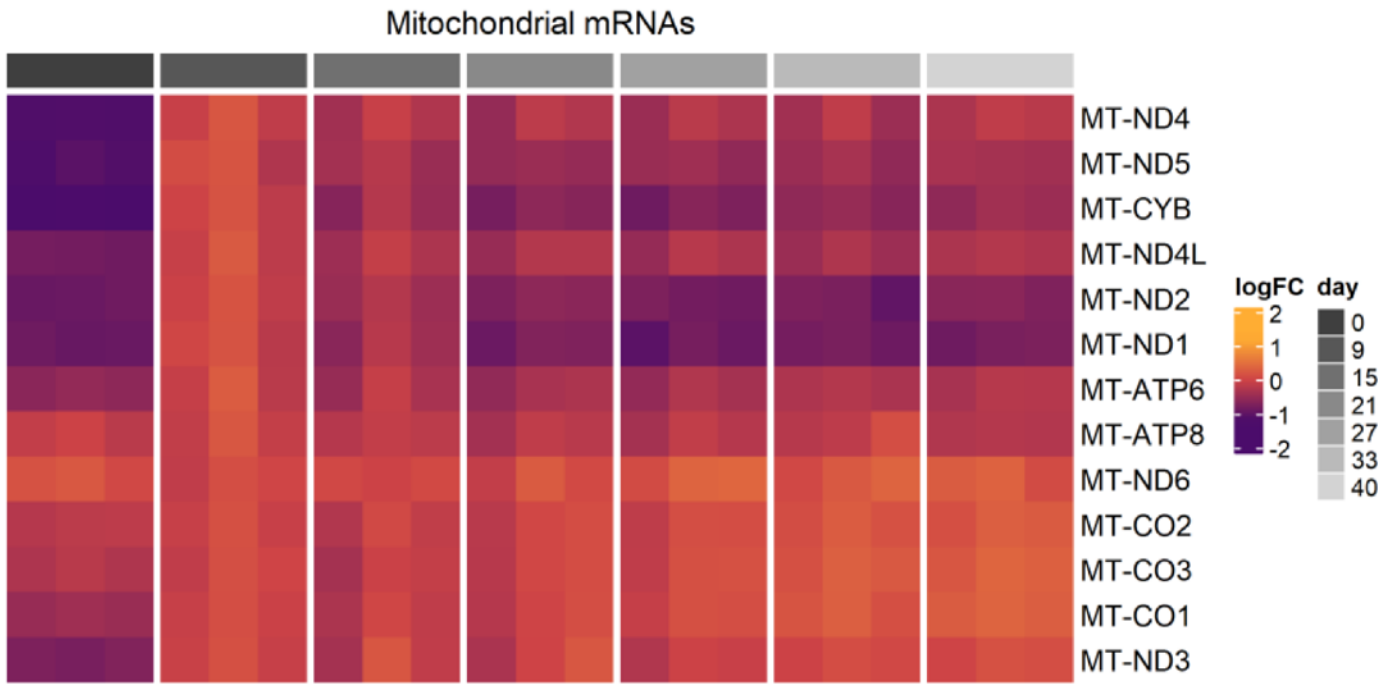

b)

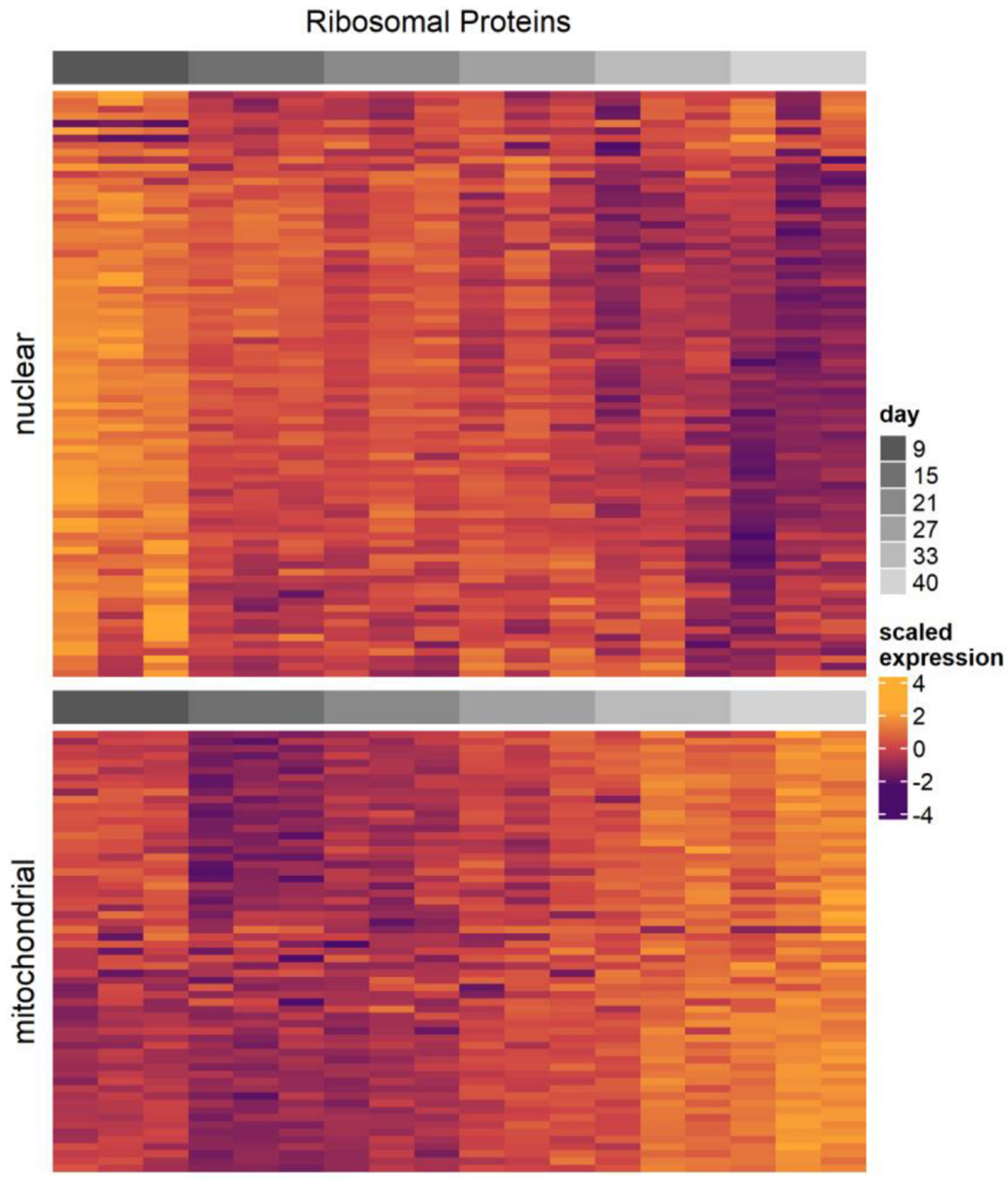

Supplementary figure S23): Expression dynamics of mitochondrial encoded genes and ribosomal proteins during human neuronal differentiation

a) Expression trajectory of genes encoded by the mitochondrial genome over the course of igNeuron differentiation. b) Heatmap of nuclear ribosomal proteins ("^RPL|^RPS"; upper panel) and mitochondrial ribosomal proteins ("^MRPL|^MRPS"; lower panel) across neuronal days over the time course of human neuronal differentiation. Shown is the scaled expression as measured by label-free proteomics (cf. Fig. 1f).

Supplementary figure S24)

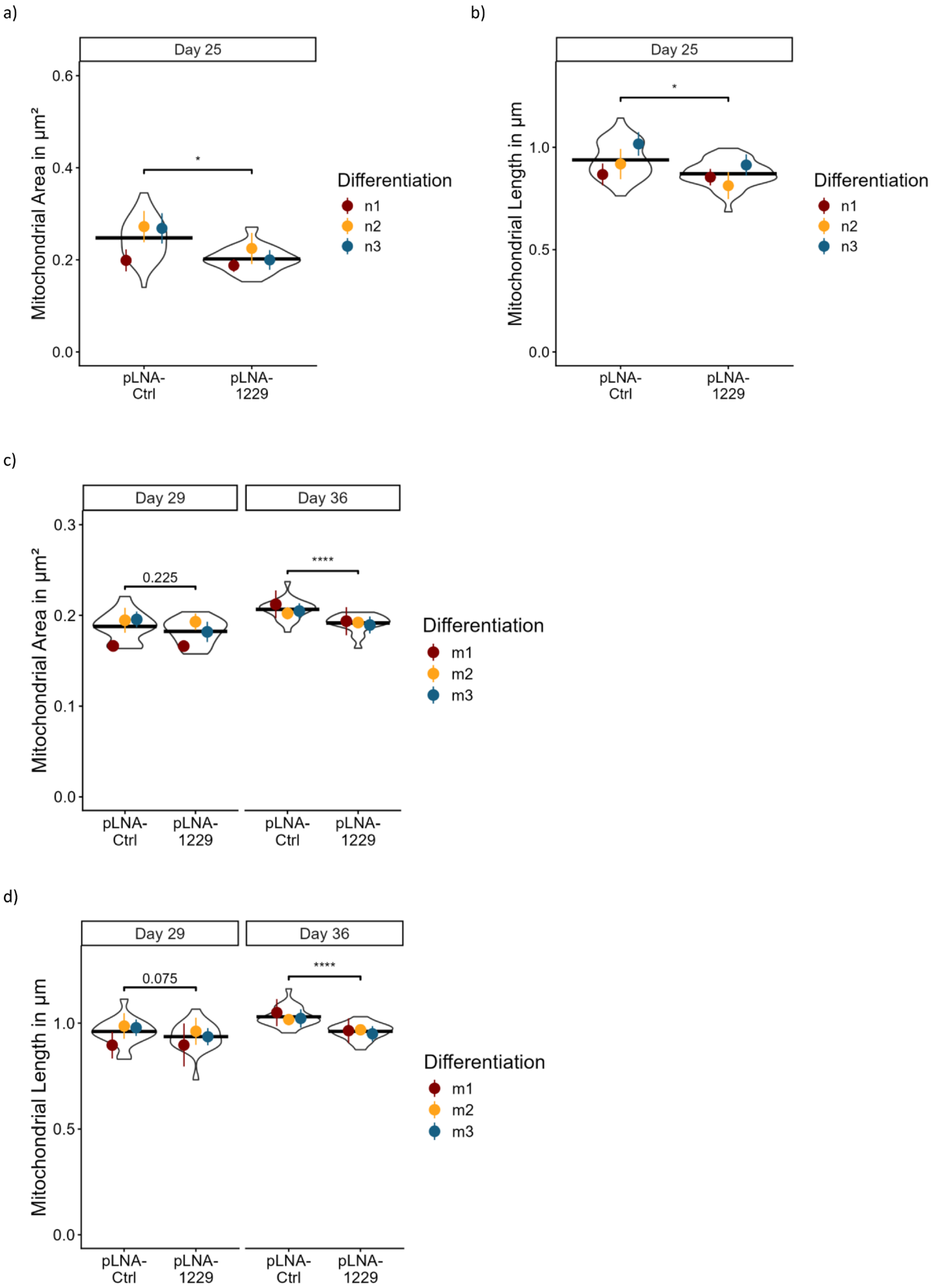

Supplementary figure S24): Morphological analysis of mitochondria at various time points of neuronal differentiation upon pLNA-treatment

a-b) Quantification of the mitochondrial area (a) and length (b) in neuronal processes of day 25 iND3 human neurons. Mitochondrial segmentation was performed on z-projected maximum intensities of images acquired with the iSIM microscope. Values were aggregated per image (mean) and subsequently per differentiation and condition. A robust linear model was applied on the resulting values for the statistical analyses (~ condition + differentiation). Post-hoc testing was performed with emmeans. Shown are violin plots of all image mean datapoints together with the mean and standard deviation of each differentiation (n = 3). c-d) Mitochondrial area (c) and length (d) quantification at two time points (day 29 and day 36) with individual images obtained from the SIM-videos of three independent differentiations (20-23 videos of each condition per time point, Fig. 5f shows the area quantification at day 36). Values were aggregated per image (mean) and subsequently per differentiation and condition. A robust linear model was applied on the resulting values for the statistical analyses (~ condition + differentiation), separated per day. Post-hoc testing was performed with emmeans. Shown are violin plots of all image mean datapoints together with the mean and standard deviation of each differentiation (n = 3).

Supplementary figure S25)

a)

b)

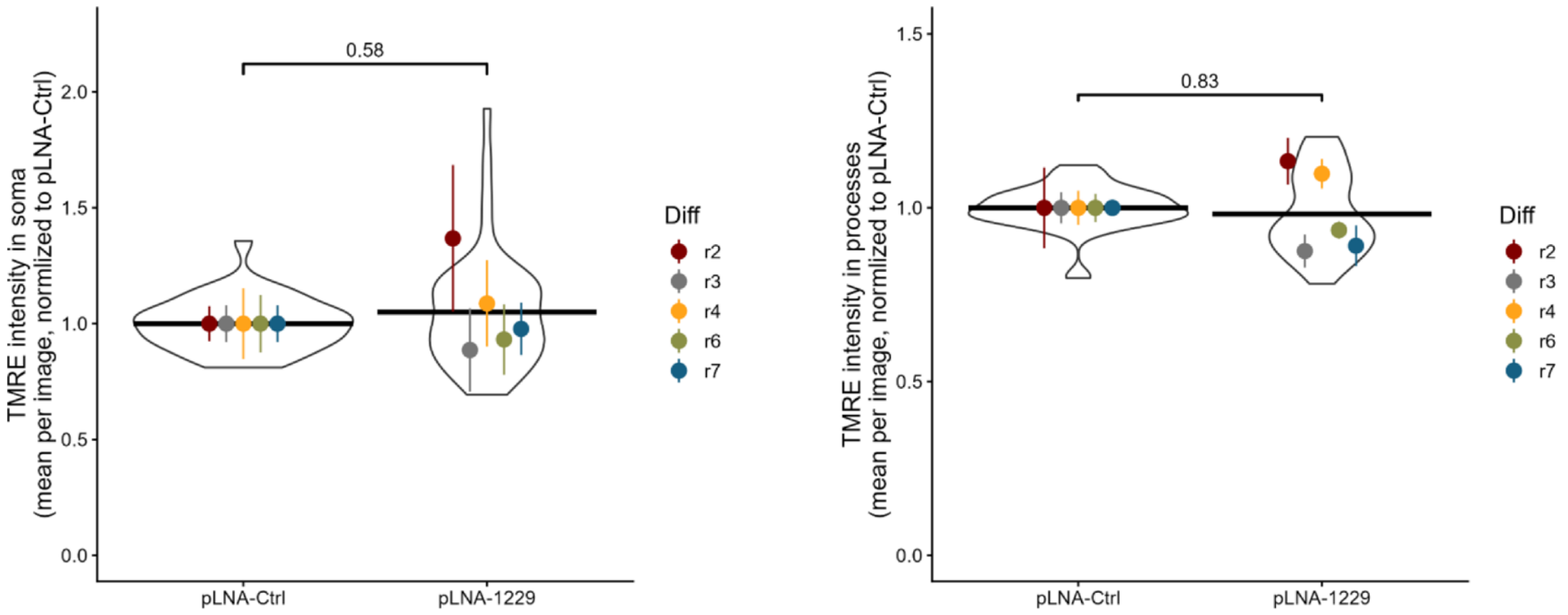

Supplementary figure S25): Quantification of the mitochondrial membrane potential upon miR-1229-3p depletion

a-b) Mitochondrial membrane potential in the soma (a) and processes (b) of igNeurons at day 23 treated with pLNA-1229 or the pLNA-Ctrl. Results were aggregated per image (mean) and then further per differentiation and condition (mean). For the statistical analyses, we applied robust linear models on the resulting five datapoints per condition (~ condition + differentiation) and used emmeans for post-hoc testing. Shown are violin plots of all image-means together with the overall mean and standard deviation normalized to the mean of the control condition of each differentiation (n = 5).

Supplementary figure S26)

a)

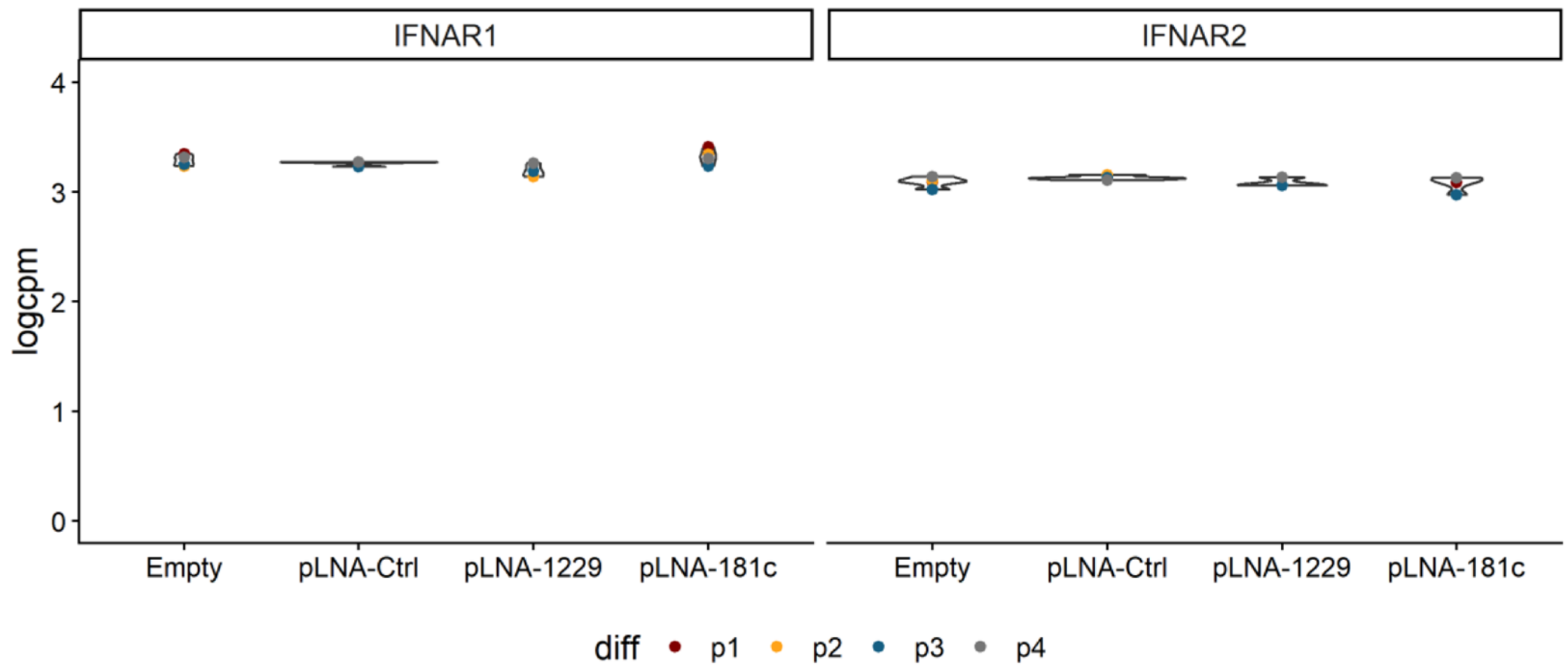

b)

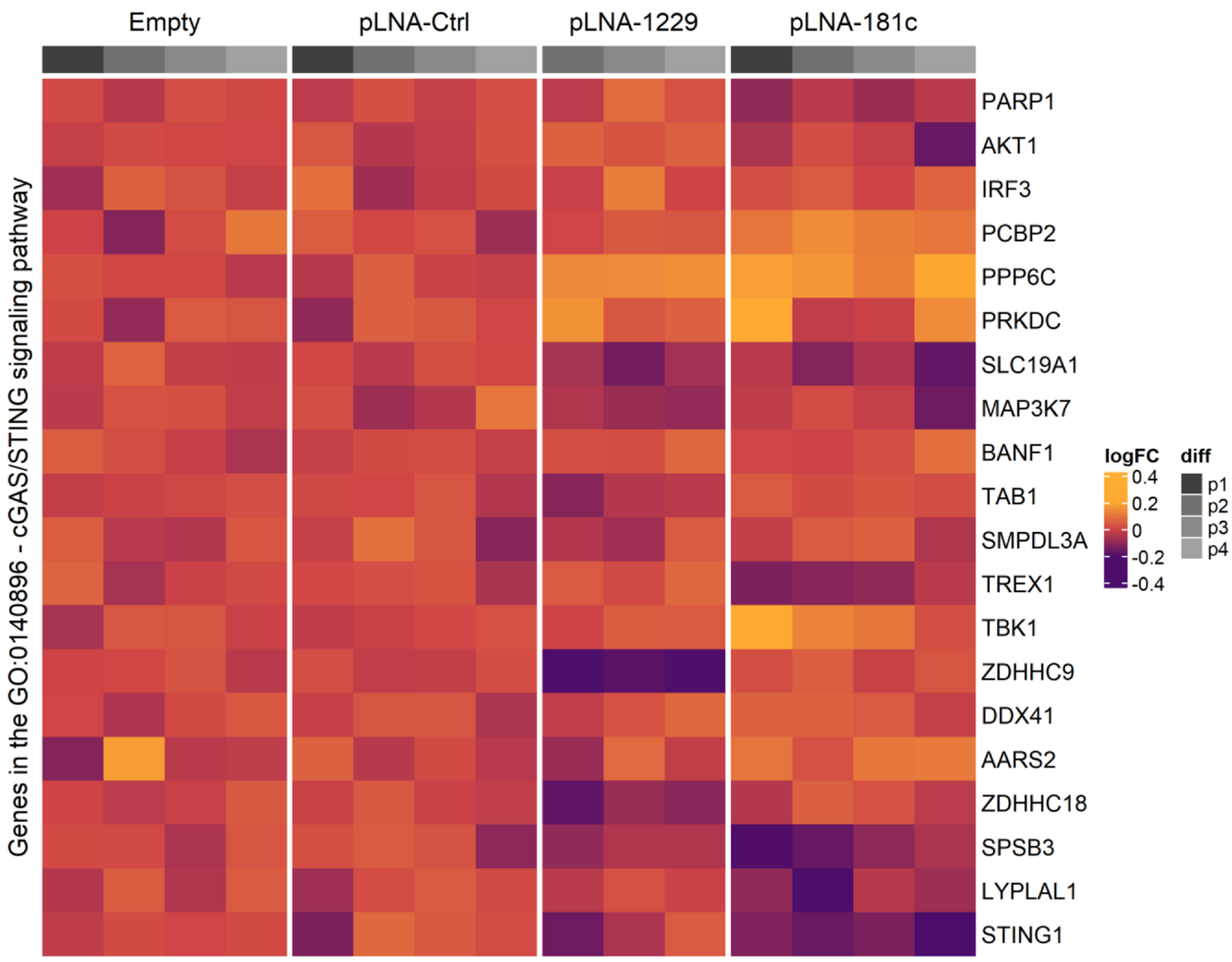

Supplementary figure S26): Expression changes of interferon receptor- and cGAS/STING signaling pathway genes upon pLNA treatment

a) Expression values (logcpm) of two interferon receptors across conditions in the pLNA RNA sequencing. There is no upregulation of either of the receptors visible in the pLNA-1229 condition. b) Heatmap indicating logFC of genes belonging to the GO-Term "GO:0140896 - cGAS/STING signaling pathway" across conditions in the pLNA RNA sequencing. There is no uniform upregulation of these genes in the pLNA-1229 condition.

Supplementary figure S27)

a)

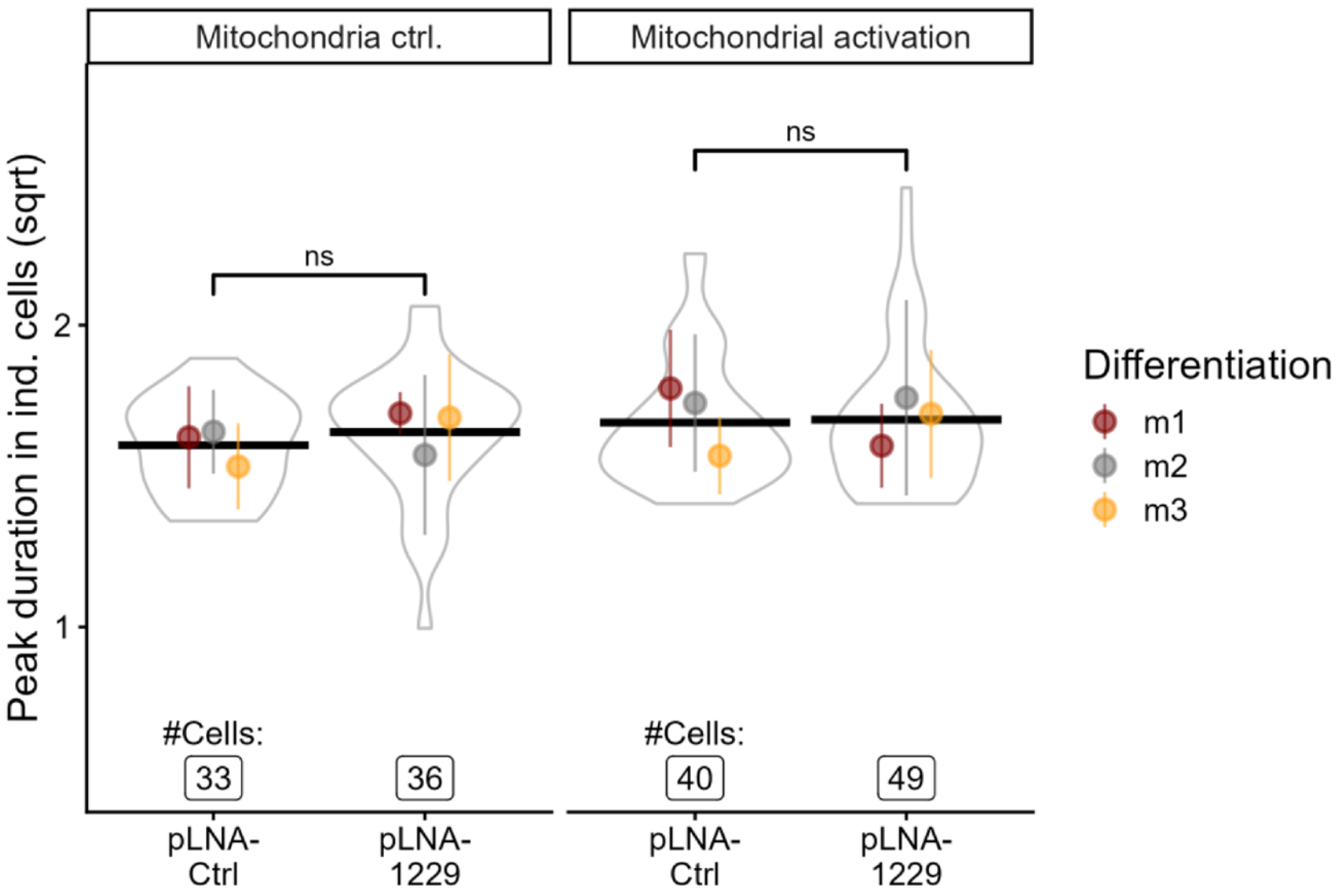

b)

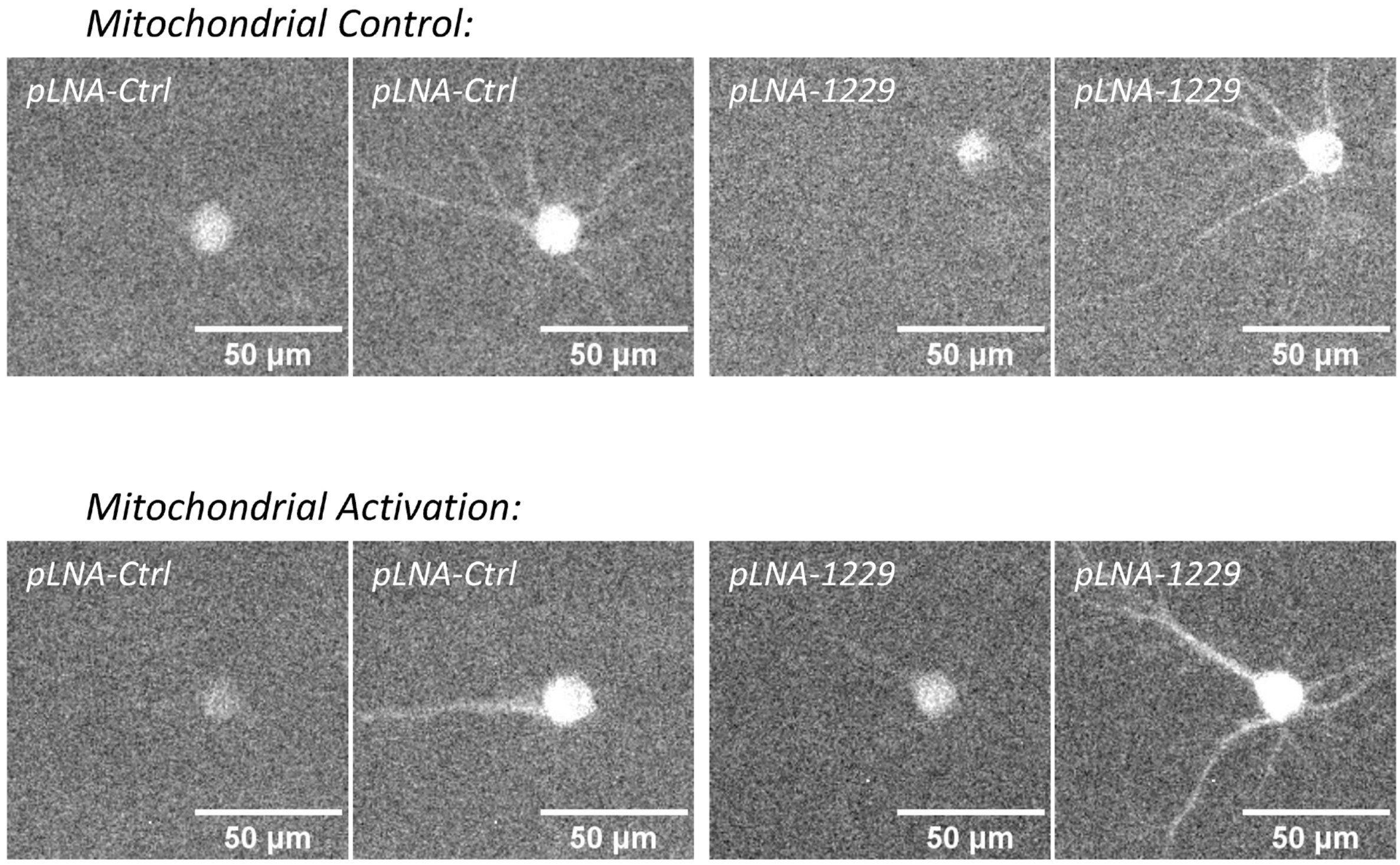

Supplementary figure S27): Supplementary information on the calcium imaging upon mitochondrial activation

a) Calcium signal peak duration measured in human neurons at day 37 in three independent differentiations. GSK-2837808A and AlbuMAX were added to the media from day 16 on to activate mitochondrial metabolism. Statistical analysis was performed on mean-aggregated values per cell with a linear model accounting for the interaction effect between the pLNA condition and the drug (x ~ pLNA*drug + differentiation). Post-hoc analysis was done using emmeans (pLNA|drug). b) Example image sections of single frames of the calcium imaging

videos used for the analyses presented in Fig. 5l-m and Suppl. Fig 27a). The Ca2+ signal detected in igNeurons upon transduction with the calcium indicator GCaMP6f is displayed in grey. The left panels in each condition indicate the signal intensity between spikes and the right panels in each condition depict the calcium signal during a spike. The conditions without mitochondrial activation (i.e. mitochondrial control) are displayed in the upper row, the conditions including GSK-2837808A and AlbuMAX addition are shown in the lower row.

Supplementary figure S28)

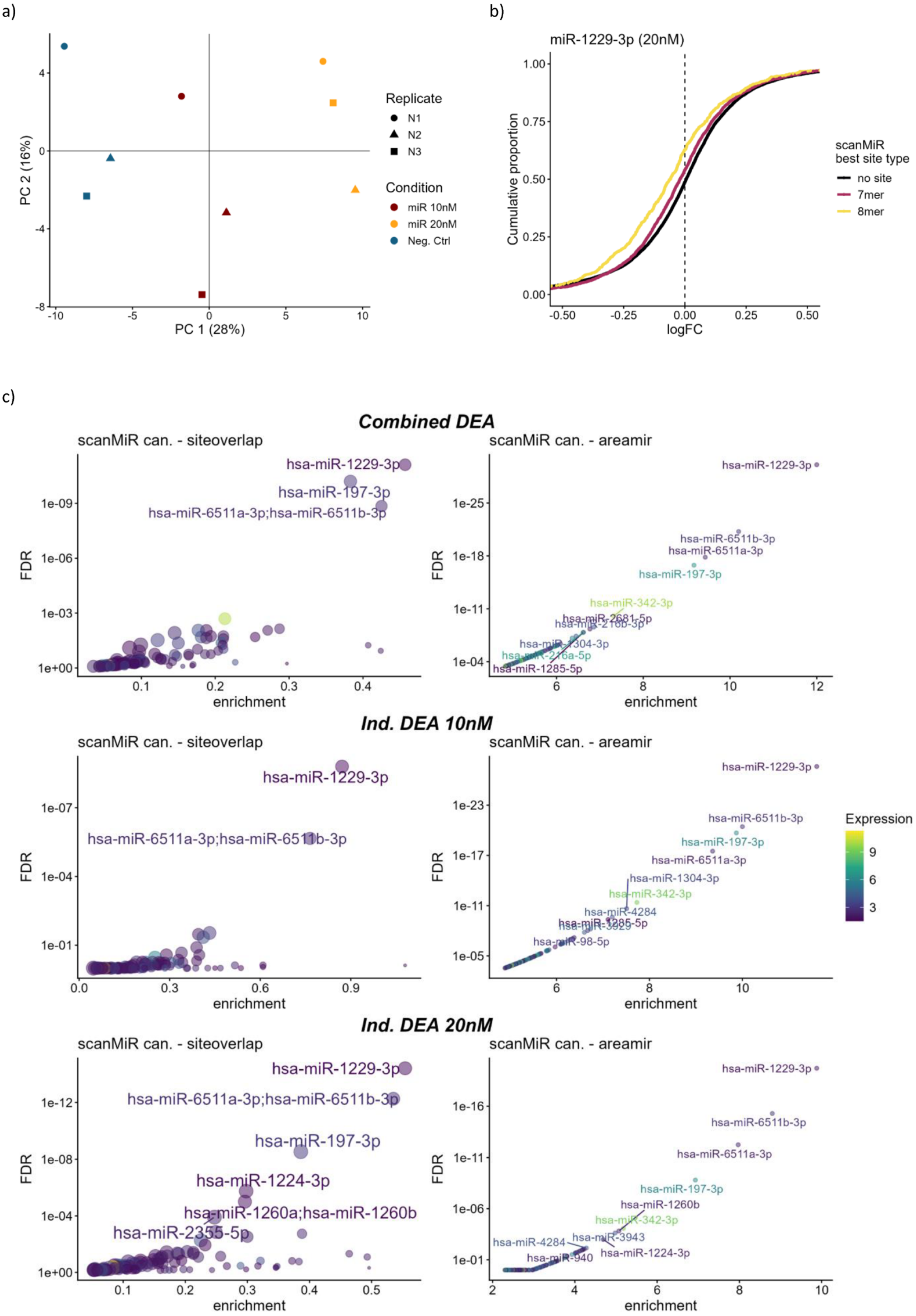

Supplementary figure S28): Principal component and enrichMiR analyses on the miR-1229-3p overexpression sequencing

a) PCA-plot of the RNA sequencing in SH-Sy5y cells upon overexpression with miR-1229-3p at two different concentrations. Conditions are separated by color and replicates indicated by shape. b) CD-plot split by site-type indicating the expected downregulation of miR-1229-3p targets upon miR-1229-3p overexpression at a concentration of 20nM. ScanMiR predictions were used to identify putative miR-1229-3p targets. c) Target enrichment plots generated with enrichMiR demonstrate significant enrichment of miR-1229-3p binding sites among downregulated genes across the three different differential expression analyses (the combined DEA accounts for the miRNA mimic amount, Ind. DEA 10nM & Ind. DEA 20nM treat the two concentrations separately; see the methods for further information). The ScanMiR database (canonical 7mer and 8mer sites in the 3'UTR) was used in conjunction with the siteoverlap test (left panels, including genes with FDR < 0.05) and areamir test (right panel). Plots display the log2-fold enrichment (siteoverlap test) or a normalized enrichment score (areamir test) on the x-axes and the FDR values of the statistical tests on the y-axes. The miRNA expression at day 21 of the time course experiments is indicated by the color. Please refer to the enrichMiR publication for details upon statistical tests and databases (*8*).

Supplementary figure S29)

a)

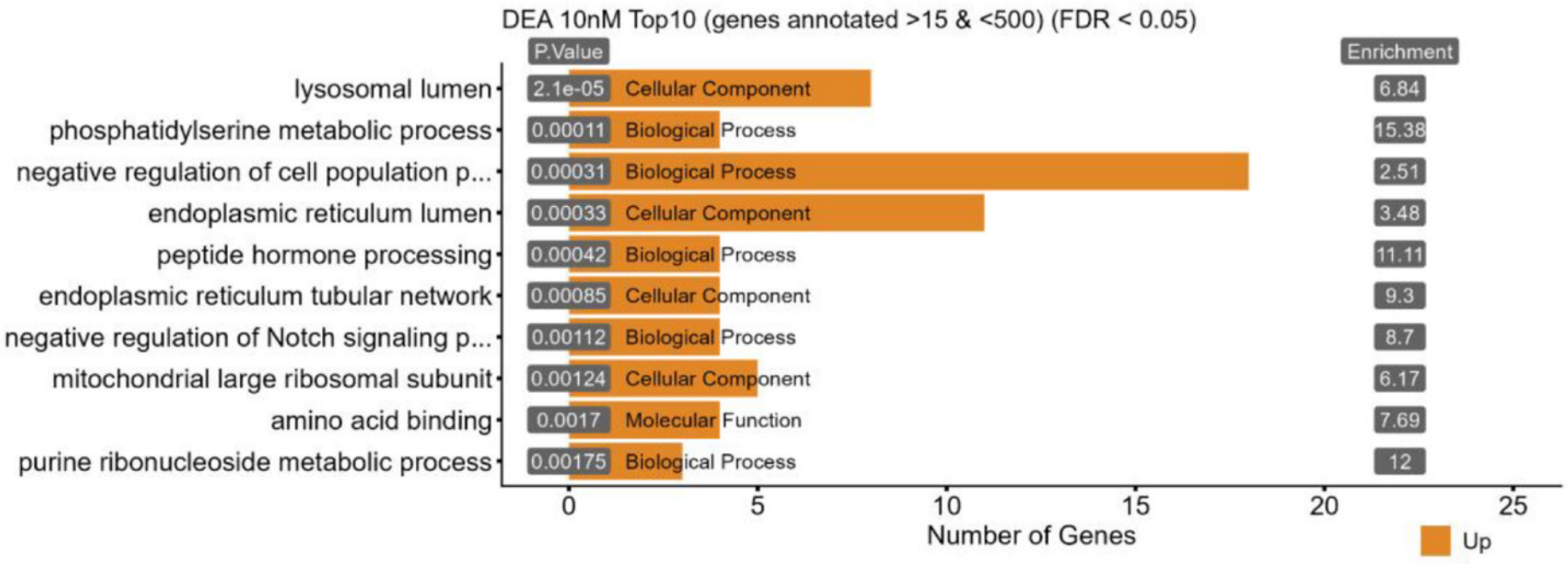

b)

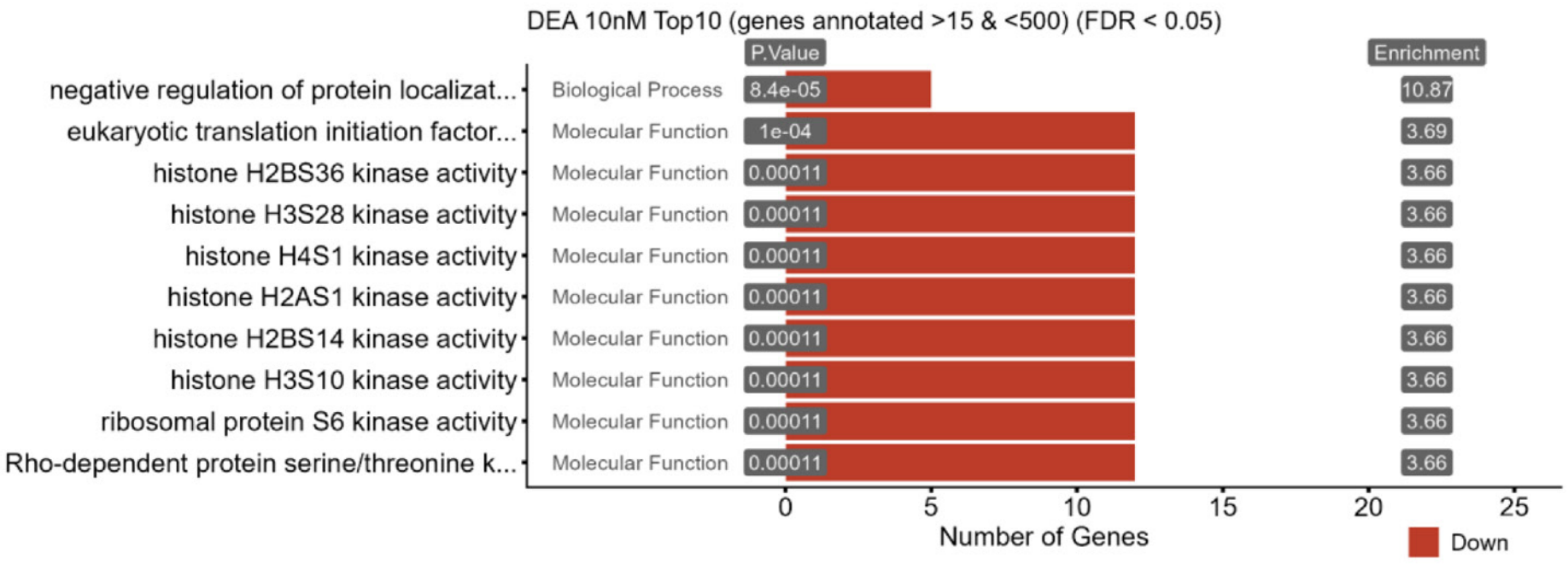

c)

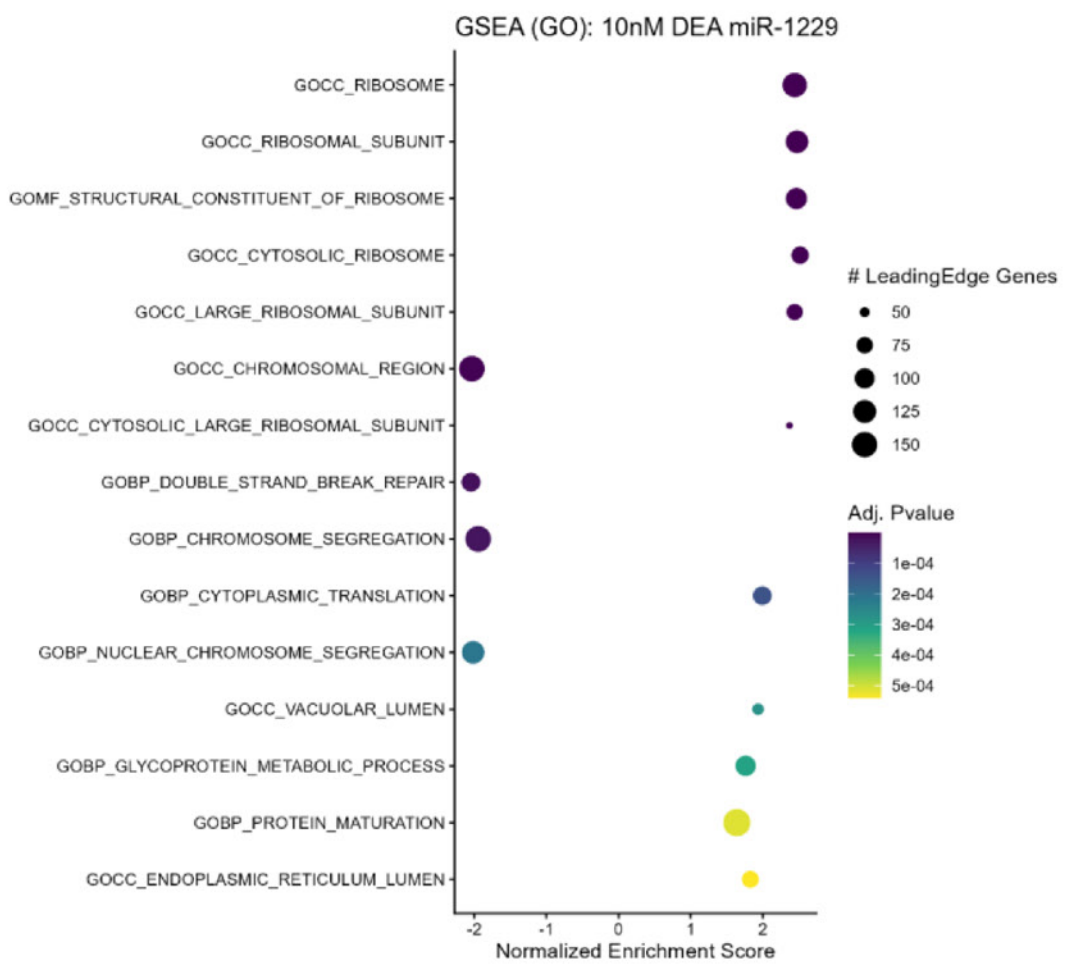

d)

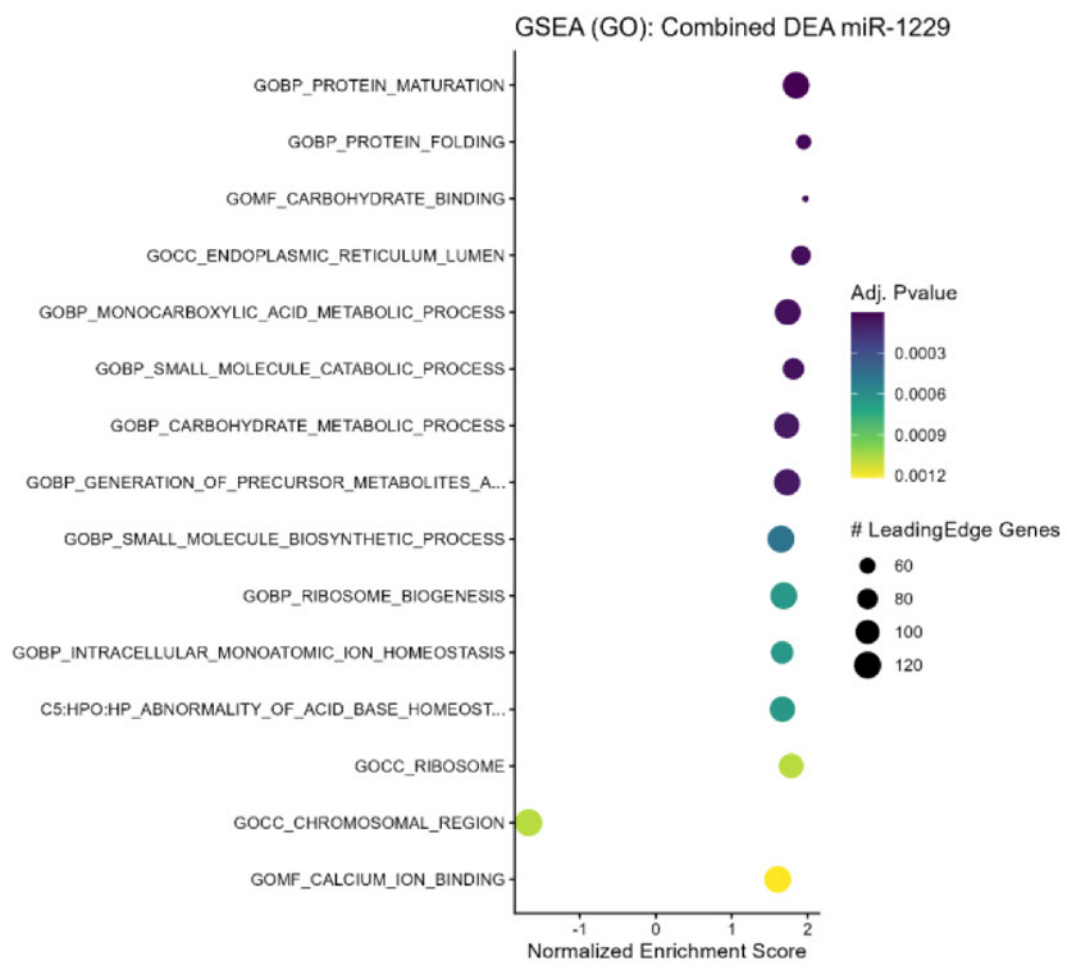

Supplementary figure S29): GO-Term and gene set enrichment analyses on the miR-1229-3p overexpression sequencing

a-b) Individual GO-Term analyses of up- (a) or down- (b) regulated genes as determined in the SH-Sy5y sequencing upon miR-1229-3p overexpression (10nM DEA, FDR < 0.05). Shown are the top 10 significant GO-Terms of all three ontologies and the number of associated genes that are significantly up- or downregulated. c-d) GSEA analyses of the 10nM DEA (c) and the combined DEA (d) using gene ontology pathways. Plotted is the normalized enrichment score

on the x-axis and the 15 most significant identified pathways on the y-axis, colored by adjusted p-value.

Supplementary figure S30)

a)

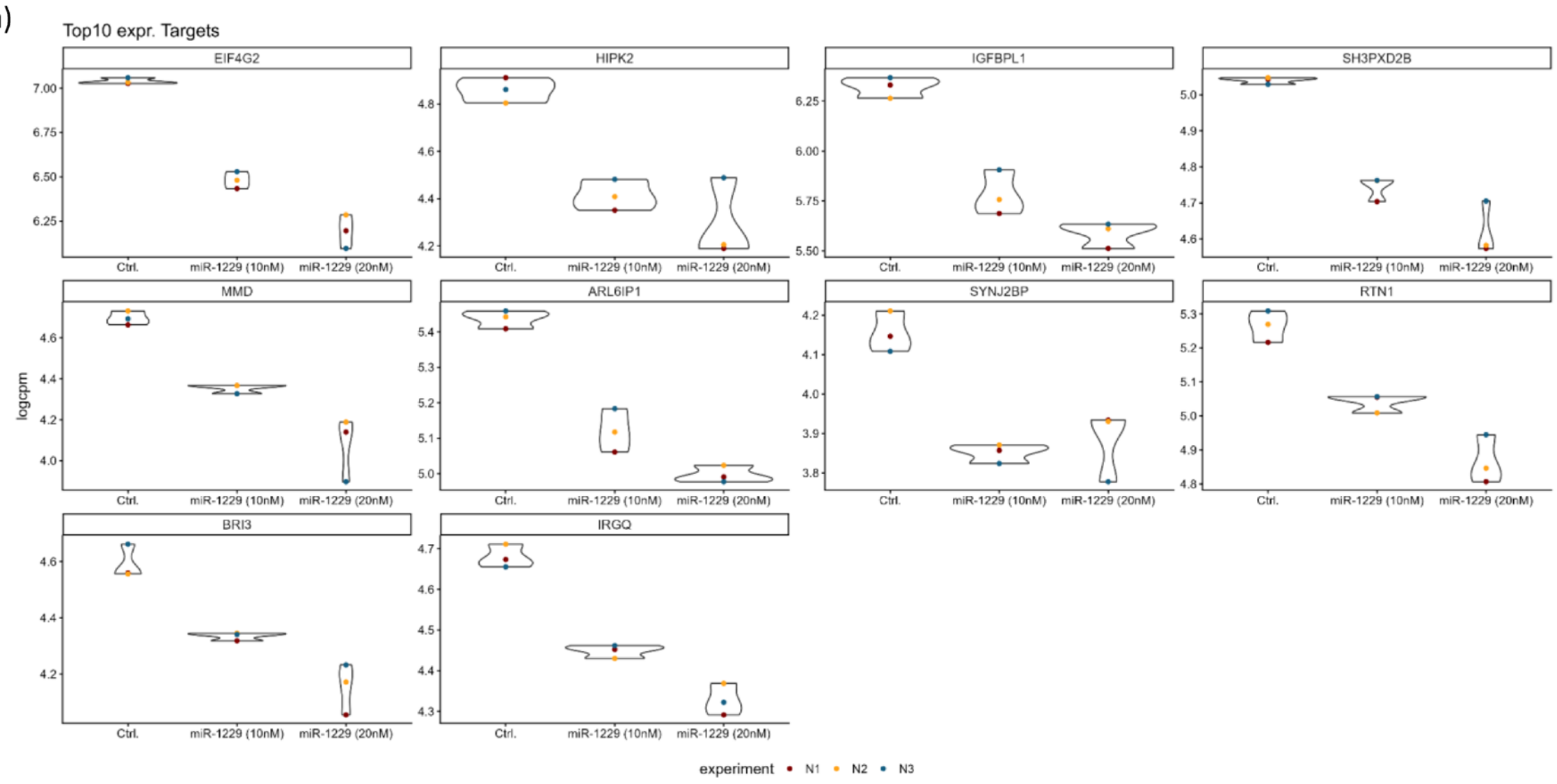

b)

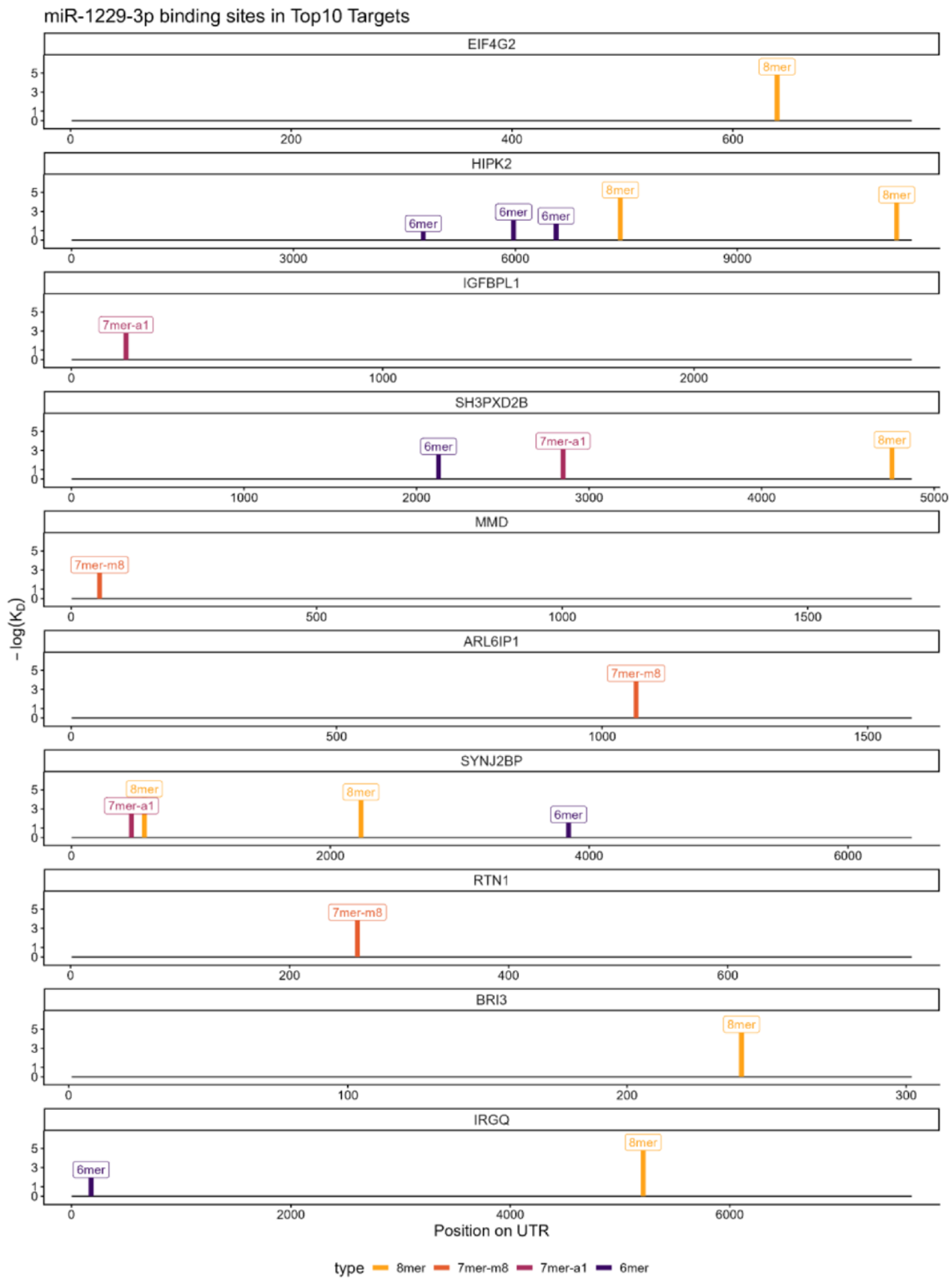

Supplementary figure S30): Identification of miR-1229-3p candidate target genes

a-b) Expression values (a) and scanMiR binding sites (b) of the top 10 most significantly downregulated genes in the miR-1229-3p overexpression sequencing dataset (10nM DEA) that

contain miR-1229-3p binding sites and are expressed in human neurons (pLNA sequencing dataset, miR-1229-3p DEA: logcpm > 2.5). Displayed are in a) the logcpm values of the Ctrl. condition, the 10nM overexpression- and the 20nM overexpression conditions. Panels in b) show the predicted miR-1229-3p binding sites in the 3'UTRs of these genes. Depicted are log(K_D) values (ScanMiR-predicted miRNA binding site affinity (*11*)) of canonical binding sites and their position on the 3'UTR.

Supplementary figure S31)

a)

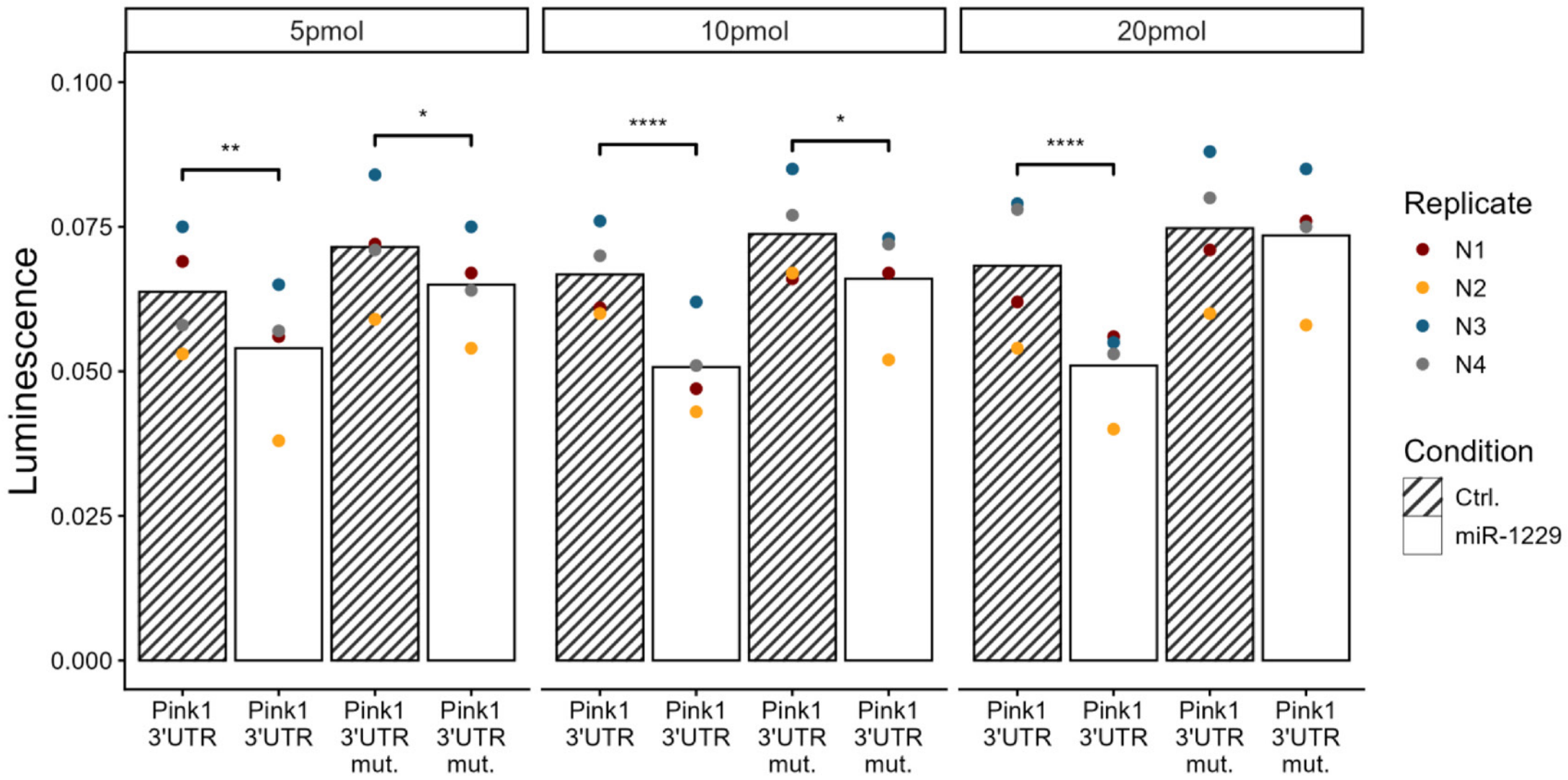

b)

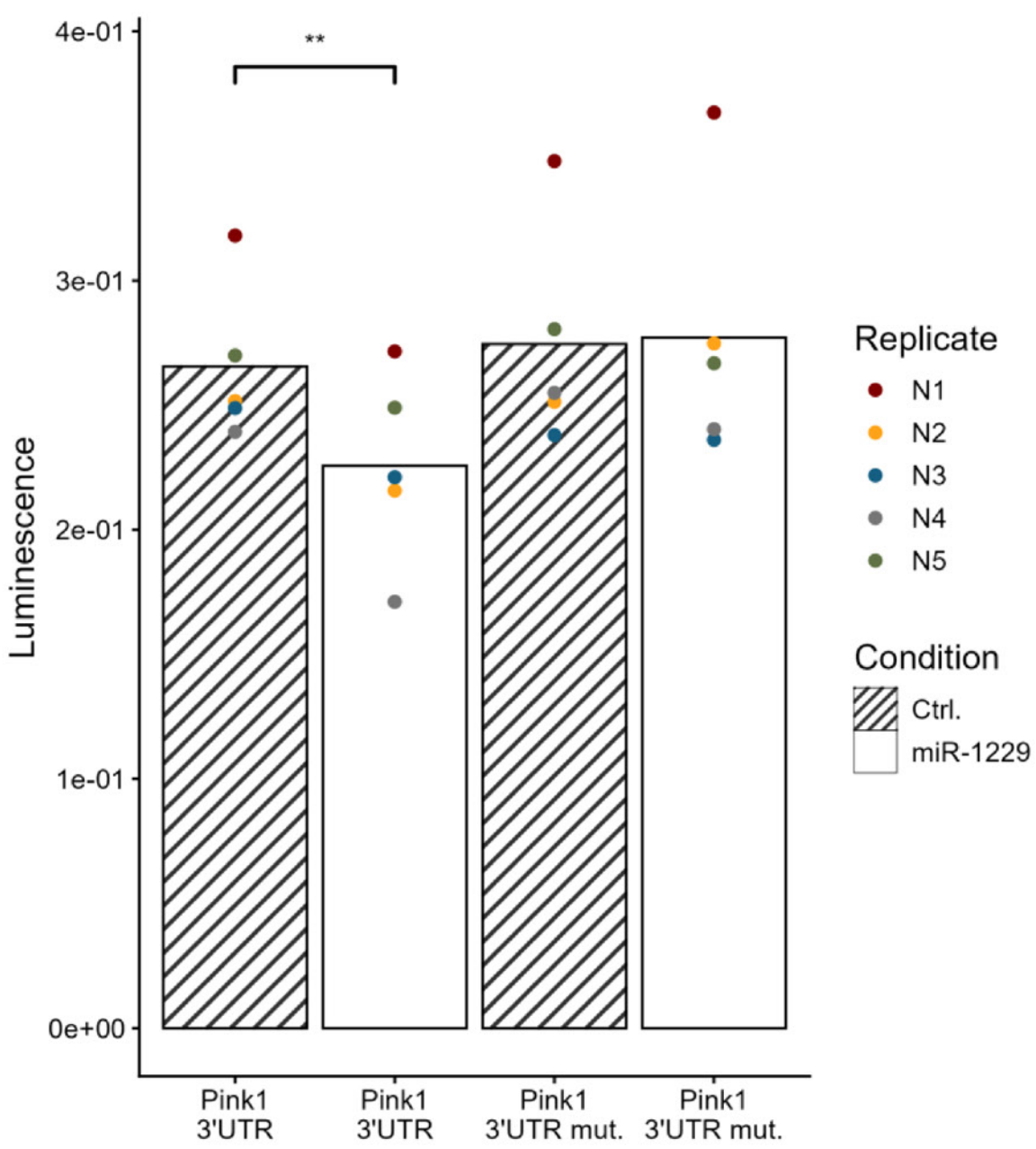

Supplementary figure S31): Luciferase measurements of the Pink1 3'UTR upon miR-1229-3p overexpression

a-b) Luciferase measurements of human Pink1 3'UTR constructs transfected into HEK-cells (a) or rat primary cortical neurons (b) together with synthetical miR-1229-3p or control (Ctrl.) mimics. "Pink1 3'UTR mut." contains three point mutations in the miR-1229-3p 8mer binding site. Two low-affinity miR-1229-3p 6mer binding sites were not mutated. Three different miR-1229-3p mimic concentrations were used for the HEK-cell experiment (5-20pmol). For the cortical neuron experiment the miRNA-mimic was transfected at a concentration of 10pmol. A linear model with an interaction effect for the miRNA mimic amount (if applicable), the sequence and miRNA mimic (including a fixed effect for the experiment) was used for the statistical analysis (~ condition*sequence*amount + experiment). Statistical comparisons were

acquired using emmeans by comparing the two conditions separately per amount (if applicable) and sequence (condition|sequence|amount, reference = Ctrl.). (a: n = 4, b: n = 5). (* = p-value < 0.05, ** = p-value < 0.01, **** = p-value < 0.0001)

Supplementary figure S32)

a)

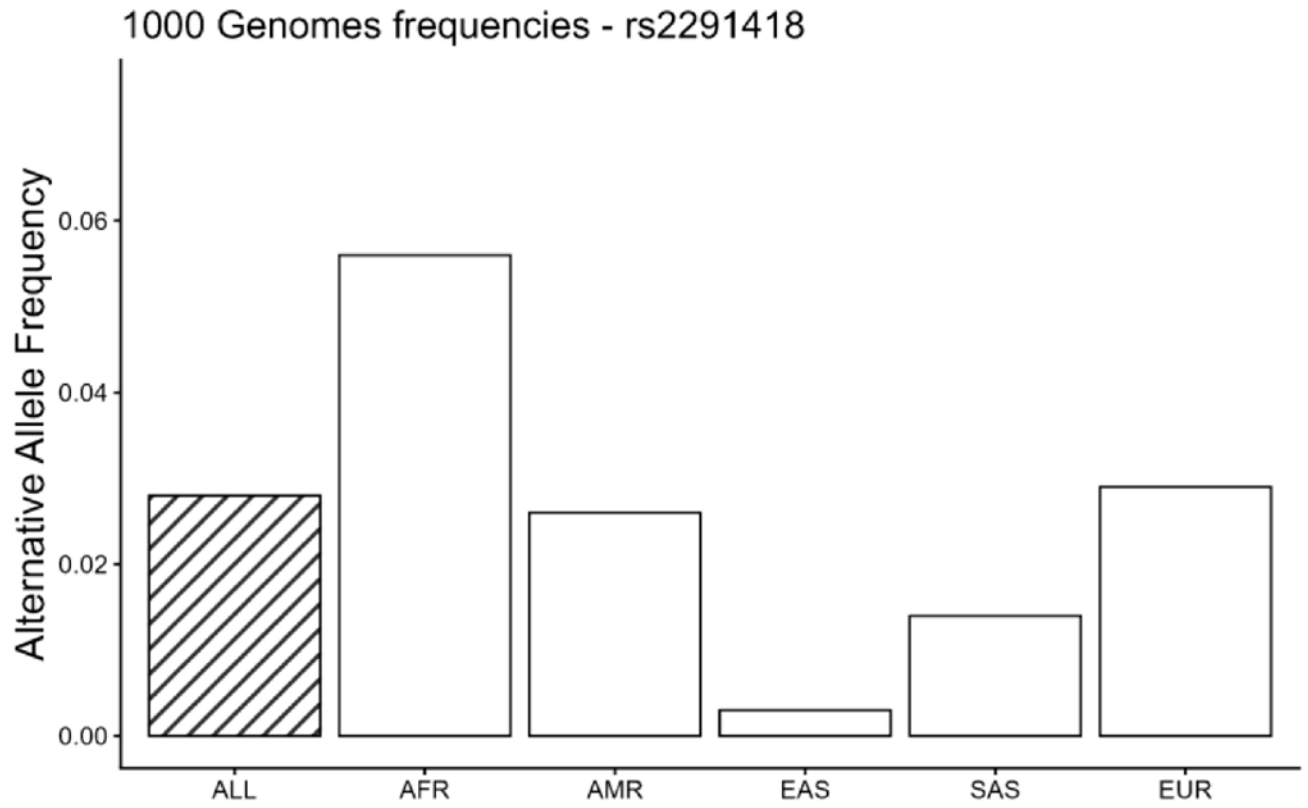

b)

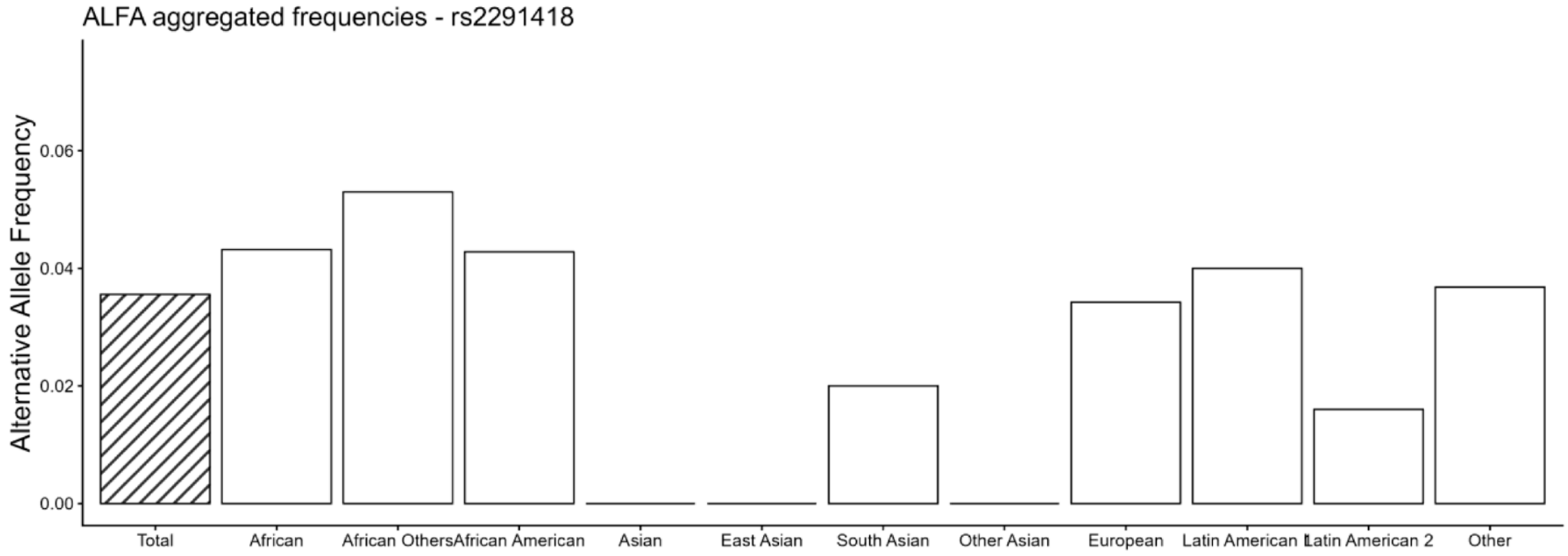

Supplementary figure S32): Allele frequencies of a SNP in the miR-1229 gene in the human population

a-b) Plots showing the alternative allele frequencies in the human population of a SNP (rs2291418) in the pre-miR-1229 sequence as determined by the 1000 Genomes (a) or the ALFA (b) project. AFR = African, AMR = American, EAS = East Asian, SAS = South Asian, EUR = European.

Supplementary Methods

Media overview for igNeuron differentiation

Neuronal induction medium:

DMEM/F12	Thermo - 11320074	
N2	Thermo - 17502048	1:100
NEAA	Thermo - 11140050	1:100
Doxycycline	Sigma - D9891	2 mg/L
Human BDNF	Peprotech - 450-02	10 ng/ml
Human NT3	Peprotech - 450-03	10 ng/ml
Mouse Laminin	Thermo - 23017015	0.2 µg/ml

Neuronal culture medium I:

NB Plus / B27 Plus	Thermo - A3653401	
Glutamax	Thermo - 35050038	*1 mM
Doxycycline	Sigma - D9891	2 mg/L
Human BDNF	Peprotech - 450-02	10 ng/ml
Human NT3	Peprotech - 450-03	10 ng/ml
Human GDNF	Peprotech - 450-10	10 ng/ml
Human CNTF	Peprotech - 450-13	10 ng/ml
Mouse Laminin	Thermo - 23017015	0.2 µg/ml
cAMP	StemCell - # 73886	0.5 mM

*NB/B27 iPSC already contains 0.5 mM of Glutamax, add therefore only 0.5mM.

Neuronal culture medium II:

NB Plus / B27 Plus	Thermo - A3653401	
Glutamax	Thermo - 35050038	*1 mM
Doxycycline	Sigma - D9891	2 mg/L
Human BDNF	Peprotech - 450-02	10 ng/ml
Human NT3	Peprotech - 450-03	10 ng/ml
Human GDNF	Peprotech - 450-10	10 ng/ml
Human CNTF	Peprotech - 450-13	10 ng/ml
Mouse Laminin	Thermo - 23017015	0.2 µg/ml
cAMP	StemCell - # 73886	0.5 mM
AraC	Sigma - C1768	50nM

*NB/B27 iPSC already contains 0.5 mM of Glutamax, add therefore only 0.5mM.

Primers:

Pink1 3'UTR

PINK1_genome_fw	AACCTGGAGTGTGAAACGCT
PINK1_genome_bw	AGATGACAGTGCTGGGGAAG
SYNJ2BP_genome_fw	AGCTCGCTAGCAAAACTTGCTCTCTTTCAATACT CCC

SYNJ2BP_genome_bw	CTGCAGGTCGACGATTACCTGCATATCTTCTTTG TTGC
PINK1_3UTR_cloning_fw	AGCTCGCTAGCTGTCCCTGCATGGAGC
PINK1_3UTR_cloning_bw	CTGCAGGTCGACTCAGTTGAAGACAACCTTTACT G
PINK1_3UTR_8mer-mut_s	TACTCTGAAGATCACAATATTTTGTGGGCAGGTA TCAACATTGG
PINK1_3UTR_8mer-mut_as	CACAAAATATTGTGATCTTCAGAGTATAAGAATC ATTCTTAAAGCC

PBS reporter

2xPBS_hsa-miR-1229-3p_s	ctagcCTGTGGGAGGGCAGTGGTGAGAGctCTGTGG GAGGGCAGTGGTGAGAGg
2xPBS_hsa-miR-1229-3p_as	tcgacCTCTCACCACTGCCCTCCCACAGagCTCTCAC CACTGCCCTCCCACAGg
2xPBS_chi-miR-1229-3p_s	ctagcCTGGGGGAGGGCAGCGCTGAGAGctCTGGGG GAGGGCAGCGCTGAGAGg
2xPBS_chi-miR-1229-3p_as	tcgacCTCTCAGCGCTGCCCTCCCCCAGagCTCTCAG CGCTGCCCTCCCCCAGg

miR-1229 Hairpin

pcDNA3_hsa1229_minigene_fw	AGA CCC AAG CTT GTT CTT CTT CCG CAG TGG
pcDNA3_hsa1229_minigene_bw	ATG CAT GCT CGA GCC TCG CTC AGA ATC ACC C
pcDNA3_chi1229_minigene_fragment	CGCCACCCTCCGGTACCCTCGGAGCCCCGACGGC TACCTCCAGATCGGTGGGTAGGGTTTGGGGGAG AGCGTGGGCTGGGGTTCGGGGACACCCTCTCAG CGCTGCCCTCCCCCAGGCTCCTTCTACAAGGGAG TGGCAGAGGGAGAGGTGGACCCAGCCTTCGGCC CTCTGGAAGCACTGCGCCTCTCGATCCAGACGGA CTCCCCTGTGTGGGTGATTCTGAGCGAGGCTCGA GCATGCATCTAGAGGGCCC

Synj2bp 2.3kb fragment with miR-1229-3p mutations:

agctcgctagcAAAACTTGCTCTCTTTCAATACTCCCAATGAAGATACATTTCACTCACC CTCCACCCCTGCTATTCTGCCATGTCTTTCCCTCTCTCTGCATAGCCAGATTTGAA GTGACTGATACCCACCCCAAACCTTGCTGTTCACAGTCTCCAATTCTTCATATTCT AATGGGAAAGTAAAGGTATTGTTTGAAGGAAAACTGAAGAAAAGACTTGGCTTA GAACAAATGAGGAGTTATATATTTTACTAGGACTTTTGATAGAAATTCAGCTACA ACCCAAAGAGAGAAAGATTGAGTCTTCCTGTCACCATAGGCAATACCTTTTTTCT TAGCTGGCATGCCATAAAGGCCAGCTATGTGATATTAGAGGAAGAAAGGATTTTT CTTTTTAATGATCTTCCTTGGGAAATTATTGTGGCCTTTATTTAATTTCTAACTACG

TACCTGGGTGCCTATATCGACAAAGAGTGtttAGAGCATTTTTACTTTTTTAAAAAA
GCAAATACATATATACACATACGTATGCAAATATTATAGTATAATAGTGATCCCT
ATGGAGAATTAAAGGTGtttAAGCTACTTTGTGGTGTCTAGGTTTCTGATAAAAGGG
ATGATCTTAACTGAAGAATTTAAAGAGATACTTAAACAGAGCAAATGTAGTAGG
AACAAGGGAGTGAGCCTTATAAGAGGACGTTCAGTCTCATTTATTAAAATAATAA
CTGAGACTGGGAGAGGTGGCTCATGCCTGTAAATCCCAGCACTTTGGTAGCCTGA
AGTGGGAGATTGCTTGAGTCCAGGAGACCAGCCTGGGCAACATAGCAAAACCTC
ATCTCTATTTAAAAAAAAAATAGAAAAAAATGTAATAACTGAAGCAACTAATTA
AAAATCTTAGAAGAACAATATTTATTCTTATAACAGCCAACTATGGtttGTTATTAT
AAGGTAAATTGCCATAATCCAGCGCTGCTCACATCTAAAAAACTTTGAGGTGTAA
AGGTAAATCGTACCAATCTCTGGGCTGCCCTAGTGACTTTTTTGATACCTCAGAGT
CCTATTGAAGAACCAGGTGGAATGATTGTGGTAAGGGCTATAGTGCTGGAGCCA
AACACCCCAGCAAGAAAACTCTAAAAGGACAAAAGATAAGGTAGTGCCTCCAAG
GTGGCACCTGAACCCTCTCAGGCTACTTTTTGGTTTCTACTAACAAACCTGAGGG
AAGGAACTGCCTCCCTGGCAGGATGGGCATCAAATCAACTCTGGCTTCAGATTTA
GTTAAAATGAGGCCGGGTGCAGCGGCTCACACCTGTCATCTCAGCACTTTGGGAG
GCCGAGGCAGGCAGATCACAAGGTCAGGAGTTCAAGACCAGCCTGGCCAACATG
GTGAAACCCCATGTCTACTGAAAATACAAAAATTACCTGGACATGGTGGCGCGTT
CCTGTAATTCCAGCTACTCAGGAGGCTGAGGCAGGAGAACTGTTTGAACCAGGAC
CTGGGAGACGGAGGTTGCAGTGAGCCAAGATCACGCCACTGCACTCCAGCCTGG
GCTACAGAGCAAGACTCCATCTCAAAAAAAGAAAGGCATGGATTGCTAGTATTTT
TGAAAGGCTTTATACACTACATACGGCTTTTAATAAACCTTTTTGCAGCAAGAGG
GAGGAAAAGAGTTTTGTTTGCTTTAGGAGGTAGGGAAATGTGGTAGGTGTTTCAT
GCGGCCTAGGAGGAGGATATGAATGAATGGATACTATGGAATGCCAGTGATCTC
GATAAGGTGtttACCATCCTGTTCATGTTCTTTTAAGGCTGGTTTTAAGCACTATGG
GTAGCTGAATTTTCTGGCTTTAAGAAAATCATTTCCATTAGCCAAATATATTGGCC
ATACTATAGGCCTTTTATAATTTTGTTAGCAAGATTATCAGGACTCAGGCAATCA
ACTGTATTTCATTTCTAGCTTGGACAGTCTGGTAATTAAATGTTGATTGGACATCA
GCTTTGTCAAGACACTTTTACAAAGAAGTACAGTGCATATCCTCCATCCCATAGG
GCTTTTGGGTTCCAACTGCGAAGACAACACTCTTATTGGCCACATAGGTAGGACA
GTGTAAGTAGGGGTCATGTTTATATTACAGACAGCAAAAGAGAAAAGTTACTGA
GAACTGGGGCGTTTGAGAAAGTTACGTGGAGGAAAGGGACAAGTTGAAGACCCT
TAAGGGCCTCACAGCCTAGTTAAGAGAATTCATAAACAGGTGtttAGTTAAAAGTA
TATCAGCAGGCTAAGTATTGAAAACAATATTAAAGGGGTACCATGCAAAACACG
ATGCAGCAACAAAGAAGATATGCAGGTAATCgtcgacctgcag

References:

1. Y. Zhang *et al.*, Rapid single-step induction of functional neurons from human pluripotent stem cells. *Neuron* **78**, 785-798 (2013).
2. H. C. Lin *et al.*, NGN2 induces diverse neuron types from human pluripotency. *Stem Cell Reports* **16**, 2118-2127 (2021).
3. S. Mayer *et al.*, Multimodal Single-Cell Analysis Reveals Physiological Maturation in the Developing Human Neocortex. *Neuron* **102**, 143-158 e147 (2019).
4. A. E. Trevino *et al.*, Chromatin and gene-regulatory dynamics of the developing human cerebral cortex at single-cell resolution. *Cell* **184**, 5053-5069 e5023 (2021).
5. A. J. Whipple *et al.*, Imprinted Maternally Expressed microRNAs Antagonize Paternally Driven Gene Programs in Neurons. *Mol Cell* **78**, 85-95 e88 (2020).
6. A. M. M. Sousa *et al.*, Molecular and cellular reorganization of neural circuits in the human lineage. *Science* **358**, 1027-1032 (2017).
7. V. Agarwal, G. W. Bell, J. W. Nam, D. P. Bartel, Predicting effective microRNA target sites in mammalian mRNAs. *Elife* **4**, (2015).
8. M. Soutschek, T. Germade, P. L. Germain, G. Schratt, enrichMiR predicts functionally relevant microRNAs based on target collections. *Nucleic Acids Res* **50**, W280-W289 (2022).
9. S. Rath *et al.*, MitoCarta3.0: an updated mitochondrial proteome now with sub-organelle localization and pathway annotations. *Nucleic Acids Res* **49**, D1541-D1547 (2021).
10. S. Müller-Dott *et al.*, Expanding the coverage of regulons from high-confidence prior knowledge for accurate estimation of transcription factor activities. *bioRxiv*, 2023.2003.2030.534849 (2023).
11. M. Soutschek, F. Gross, G. Schratt, P. L. Germain, scanMiR: a biochemically based toolkit for versatile and efficient microRNA target prediction. *Bioinformatics* **38**, 2466-2473 (2022).
