# Supplementary material for: A human-specific microRNA controls the timing of excitatory synaptogenesis
Source: Nat Commun. 2026 Jul 6;17:8365. doi: 10.1038/s41467-026-74753-y (PMC13473650; doi:10.1038/s41467-026-74753-y)
Supplement: Supplementary file 2 [file 41467_2026_74753_MOESM2_ESM.pdf]

Description of Additional Supplementary Files

File name: Supplementary Data S1
Description: Statistical information on the main figures

File name: Supplementary Data S2
Description: Genes used to plot the smoothed overlaps in Fig. 1f. Displayed are logcpm values for the ribosomal depletion sequencing and imputed values for the proteins obtained by proteomics.

File name: Supplementary Data S3
Description: Human miRs with orthologs

File name: Supplementary Data S4
Description: Human miRs without orthologs

File name: Supplementary Data S5
Description: Number of mismatches in precursor sequences

File name: Supplementary Data S6
Description: Seed mismatches in orthologs

File name: Supplementary Data S7
Description: Human specific seed mismatches

File name: Supplementary Data S8
Description: Significantly downregulated genes (FDR < 0.05) in the miR-1229-3p overexpression experiment that contain miR-1229-3p binding sites in their 3'UTR (7mer or 8mer).
