# Supplementary material for: A human-specific microRNA controls the timing of excitatory synaptogenesis
Source: Nat Commun. 2026 Jul 6;17:8365. doi: 10.1038/s41467-026-74753-y (PMC13473650; doi:10.1038/s41467-026-74753-y)
Supplement: Supplementary file 3 [file 41467_2026_74753_MOESM4_ESM.pdf]

nature portfolio

Corresponding author(s): Gerhard Schratt

Last updated by author(s): Nov 6, 2023

Reporting Summary

Nature Portfolio wishes to improve the reproducibility of the work that we publish. This form provides structure for consistency and transparency in reporting. For further information on Nature Portfolio policies, see our Editorial Policies and the Editorial Policy Checklist.

Statistics

For all statistical analyses, confirm that the following items are present in the figure legend, table legend, main text, or Methods section.

n/a	Confirmed	
☐	☒	The exact sample size (*n*) for each experimental group/condition, given as a discrete number and unit of measurement
☐	☒	A statement on whether measurements were taken from distinct samples or whether the same sample was measured repeatedly
☐	☒	The statistical test(s) used AND whether they are one- or two-sided<br>*Only common tests should be described solely by name; describe more complex techniques in the Methods section.*
☐	☒	A description of all covariates tested
☐	☒	A description of any assumptions or corrections, such as tests of normality and adjustment for multiple comparisons
☒	☐	A full description of the statistical parameters including central tendency (e.g. means) or other basic estimates (e.g. regression coefficient) AND variation (e.g. standard deviation) or associated estimates of uncertainty (e.g. confidence intervals)
☒	☐	For null hypothesis testing, the test statistic (e.g. *F*, *t*, *r*) with confidence intervals, effect sizes, degrees of freedom and *P* value noted<br>*Give P values as exact values whenever suitable.*
☒	☐	For Bayesian analysis, information on the choice of priors and Markov chain Monte Carlo settings
☒	☐	For hierarchical and complex designs, identification of the appropriate level for tests and full reporting of outcomes
☒	☐	Estimates of effect sizes (e.g. Cohen's *d*, Pearson's *r*), indicating how they were calculated

Our web collection on statistics for biologists contains articles on many of the points above.

Software and code

Policy information about availability of computer code

Data collection	Software applications and raw data are listed in the Data availability section of the paper as well as the methods. Further codes used in the paper are available at https://github.com/michasou/Soutschek_et_al.-2023
Data analysis	Data analysis was performed as described in the methods, mainly using R (4.5.0), Cell profiler (3 & 4) and Ilastik (1.3.3) for some mitochondrial segmentation. DNA sequences for cloning were analyzed using SerialCloner2 and SnapGene (8.2). SpectronautTM (Biognosys, version 19) was used for the proteomics analysis. Salmon (1.8 & 1.10) and STAR (2.7.0) were used for RNA-seq analysis. The mitochondrial SNP analysis was conducted with samtools.

For manuscripts utilizing custom algorithms or software that are central to the research but not yet described in published literature, software must be made available to editors and reviewers. We strongly encourage code deposition in a community repository (e.g. GitHub). See the Nature Portfolio guidelines for submitting code & software for further information.

Data

Policy information about availability of data

All manuscripts must include a data availability statement. This statement should provide the following information, where applicable:

- Accession codes, unique identifiers, or web links for publicly available datasets
- A description of any restrictions on data availability
- For clinical datasets or third party data, please ensure that the statement adheres to our policy

See the data availability statement at the end of the paper.

Research involving human participants, their data, or biological material

Policy information about studies with human participants or human data. See also policy information about sex, gender (identity/presentation), and sexual orientation and race, ethnicity and racism.

Reporting on sex and gender	see methods
Reporting on race, ethnicity, or other socially relevant groupings	NA
Population characteristics	NA
Recruitment	NA
Ethics oversight	NA

Note that full information on the approval of the study protocol must also be provided in the manuscript.

Field-specific reporting

Please select the one below that is the best fit for your research. If you are not sure, read the appropriate sections before making your selection.

[x] Life sciences [ ] Behavioural & social sciences [ ] Ecological, evolutionary & environmental sciences

For a reference copy of the document with all sections, see nature.com/documents/nr-reporting-summary-flat.pdf

Life sciences study design

All studies must disclose on these points even when the disclosure is negative.

Sample size	Three to four biological replicates (distinct differentiations) were used for most experiments (see methods) to allow statistic assessment of the hypothesis to test.
Data exclusions	See methods (one sample had to be excluded in the pLNA-RNA-sequencing experiment due to moderate degradation in the SMARTer amplification). For the mtKeima experiment we excluded differentiations with low virus transduction and hence lower signal intensity
Replication	We assume that the presented data can be replicated using the information provided in the methods.
Randomization	Specific sample randomization was applied for Proteomics and the time course as well as the pLNA RNA-Seq experiments, as well as for the data analysis of the two experiments that were not recorded blinded (see below).
Blinding	See methods: All experiments were performed and analyzed blinded, except for the calcium imaging and mitochondrial super-resolution live cell imaging that were recorded non-blinded.

Reporting for specific materials, systems and methods

We require information from authors about some types of materials, experimental systems and methods used in many studies. Here, indicate whether each material, system or method listed is relevant to your study. If you are not sure if a list item applies to your research, read the appropriate section before selecting a response.

Materials & experimental systems

n/a	Involved in the study	
[ ]	[x]	Antibodies
[ ]	[x]	Eukaryotic cell lines
[x]	[ ]	Palaeontology and archaeology
[x]	[ ]	Animals and other organisms
[x]	[ ]	Clinical data
[x]	[ ]	Dual use research of concern
[x]	[ ]	Plants

Methods

n/a	Involved in the study	
[x]	[ ]	ChIP-seq
[x]	[ ]	Flow cytometry
[x]	[ ]	MRI-based neuroimaging

Antibodies

Antibodies used	Antibodies in the following dilutions were used for the experiments: MAP2 (Thermo - PA1-16751, 1:2000), SYN1 (Merck - AB1543, 1:1000), PSD95 (Biolegend – 810401, 1:200), LAMP2 (Novus biologicals - NB300-591, 1:250), TUJ1 (Lucerna-Chem / Covance - 801201 // MMS-435P-100, 1:2500), SOX2 (Merck - MAB4343, 1:1000), GFAP (Antibody has been a gift, 1:1000) and MAP2 (Sigma - M9942, 1:4000). Secondary antibodies include Hoechst, Phalloidin (Abcam - ab176759, 1:1000) and Thermo Alexa Fluor secondary

	antibodies diluted 1:1000 (specifically 647-goat-anti-chicken (A-21449), 546-goat-anti-chicken (A-11040), 488-donkey-anti-mouse (A-21202), 647 goat-anti-rabbit (A27040) and 488-goat-anti-rabbit (A-11008). Some secondary antibodies were at some point switched to the "Alexa Plus" version.
Validation	Antibodies were not further validated besides the manufacturers information provided on their homepages.

Eukaryotic cell lines

Policy information about cell lines and Sex and Gender in Research

Cell line source(s)	HEK-293 cells (Merck – 85120602-1VL) and SH-Sy5y neuroblastoma cells (ECACC - kindly provided by Paul Johnson) were used in this study. Most human neuron experiments were performed with the SANi002-A induced pluripotent stem cell (iPSC) line (originated from a female donor). Ca-imaging was additionally conducted on MML6838.Cl2 cells (originated from a male donor)
Authentication	Cell lines were not further authenticated besides previous publications.
Mycoplasma contamination	Cell lines were not tested regularly for Mycoplasma.
Commonly misidentified lines (See ICLAC register)	No contaminated cell lines were used. HEK cells were bought from Sigma / Merck (85120602)

Plants

Seed stocks	NA
Novel plant genotypes	NA
Authentication	NA
