# Supplementary material for: A human-specific microRNA controls the timing of excitatory synaptogenesis
Source: Nat Commun. 2026 Jul 6;17:8365. doi: 10.1038/s41467-026-74753-y (PMC13473650; doi:10.1038/s41467-026-74753-y)
Supplement: Supplementary file 4 [file 41467_2026_74753_MOESM5_ESM.pdf]

nature portfolio

Peer Review File

A human-specific microRNA controls the timing of excitatory synaptogenesis

Corresponding Author: Professor Gerhard Schratt

This file contains all reviewer reports in order by version, followed by all author rebuttals in order by version.

Version 0:

Reviewer comments:

Reviewer #1

(Remarks to the Author)
Thanks to the editors for inviting me to comment on this manuscript.

Soutschek et al. present a study that explores the contribution that non-coding genes might make to the relative slowing of human neuronal maturation (neoteny). Using a glial free human iPSC-derived neuronal model (igNeurons), they present data showing widespread expression changes of non-coding RNAs, including microRNAs, during excitatory synapse development. They focus on a human-specific microRNA, miR-1229-3p, which specifically correlates with neuronal (and thus likely synaptic) maturation in this system. Inhibition of this particular microRNA appears to accelerate neuronal maturation, enhance Ca2+ transients and increase synaptic transmission. This potentially works via regulation of PINK1. Boosting mitochondrial metabolism reduces Ca2+ spiking activity, partially rescuing the impact of inhibition of miR-1229-3p.

The rationale for focusing on miR-1229-3p appears solid, since (as the authors show) its sequence (and secondary structure) is not conserved in other species and its expression tracks with synaptogenesis. Furthermore, human (but not chimp equivalent) miR-1229 mirtron can downregulate miR-1229-3p expression, inferred via a luciferase binding site reporter assay.

The authors assess the impact of miR-1229-3p KD by applying ASOs in igNeuron cultures from d9. Synaptic numbers (and sEPSC charge transfer) are increased at d21 but not d33, whereas there is decreased dendritic complexity at the later time point. Nevertheless, the effect sizes here are relatively modest. Transcriptomics reveals many DEGs associated with mitochondrial function, including downregulation of mtDNA encoded genes, and Pink1 upregulation. They then show that Pink1 is a direct target of miR-1229-3p. Human neurons treated with pLNA-1229 (hence derepressed Pink1) have mitochondria with disturbed MMP, more colocalisation with LAMP2 (suggesting mitophagosomes, the authors claim), elevated fragmentation (suggesting enhanced mitophagy, the authors claim), higher numbers of axonal mitochondria in pLNA-1229 condition (using mitotracker signal), increased lactate concentration in media (suggesting shift to glycolysis) and increased Ca2+ signals. Increasing mitochondrial metabolism via GSK-2837808A and AlbuMAX restored normal Ca2+ signal amplitude.

Overall, I found this to be a very interesting study. Many elements of the manuscript are very solid and well explained, and the findings are of significance to the field.

I have some general comments:

1. For me, to really clinch it would be to apply this miRNA to mouse neurons, and see a delay in synapse development and maturation, via Pink1 activation. Has this been attempted?
2. Since effect sizes are quite modest, in my opinion the authors' claim "that miR-1229 depletion leads to *pronounced* morphological and functional changes during human excitatory synapse development" is perhaps overstated.

Also, I have some queries regarding the data shown in Fig. 5:

1. In Fig. 5a, c, d, Mitotracker intensity overall looks altered in pLNA-1229 neurons compared to controls, possibly reflecting altered MMP. Is MMP altered in this condition? Could decreased MMP be reducing mitochondrial Ca2+ uptake, accounting

for why cytosolic Ca2+ buffering is impaired? The authors could test directly whether mitochondrial Ca2+ uptake is impaired in pLNA-1229 by using a fluorescent reporter of mitochondrial Ca2+ (e.g. mito-GCaMP or similar).
2. What does “mitochondrial entropy” mean exactly in 5d, and how is this calculated? These points were not clear to me, from the information available.
3. For the images shown in Fig 5b, please can each channel be displayed separately? Co-localisation of Mitotracker puncta with LAMP2 should be quantified in pLNA-1229 neurons and controls.
4. To support their claim of increased mitophagy, a reporter of acidified mitochondria (e.g. mito-QC, mito-SRAI, mito-Keima) could be used to demonstrate incorporation of mitochondria into an acidified compartment (e.g. mitophagosome), and/or co-labelling with parkin to demonstrate parkin recruitment to mitochondria.
5. In Fig. 5f and g, I would suggest imaging mitochondria with a non-MMP dependent marker (e.g. mitoDsRed or similar) to quantify mitochondria in axons to avoid the potential confounder of mitochondria with depleted MMP (perhaps more likely to be present in the pLNA-1229 neurons) not being visualised with a dye that is MMP dependent.
6. In Fig. 5j it would be helpful to see some representative micrographs of the Ca2+ imaging, as well as quantification.
7. How do the authors propose that mitochondrial activation (with Albumax or the LDHA inhibitor) leads to increased cytoplasmic Ca2+ buffering? Do they observe an increase in mitochondrial mass, MMP, change in morphology, and/or increased interaction with ER, when these treatments are administered?

Reviewer #2

(Remarks to the Author)
Soutschek et al. established an experimental system that allows human neurons to form synapses in a culture environment without feeder astrocytes, and by effectively using this system, showed that miR-1229-3p, miRNAs with human-specific sequences, can slow down excitatory synapse formation. The regulation of gene expression by this miRNA alters mitochondrial function and may thus control the timing of neuronal maturation, typically synaptogenesis. This is consistent with the results of several recent papers showing that mitochondria-related metabolic control regulates the rate of developmental progression, and in particular with a recent paper (Iwata et al. Science) showing that mitochondria regulate the rate of human neuronal maturation. The evolutionary mechanisms of human brain development are a topic of interest to many researchers, and the finding in this manuscript that human-specific miRNA may be relevant to the protracted neuronal maturation uniquely observed in humans is intriguing.

On the other hand, the paper has obvious shortcomings. All experiments were performed in igNeuron, an artificial experimental system where synapse formation can be observed dramatically earlier than in vivo by introducing the Ngn2 gene into iPS cells, and there is no evidence that miR-1229-3p actually works under human physiological conditions. Therefore, revisions with additional analyses bridging the results of in vitro experiments and in vivo physiology will significantly increase the impact of the manuscript. In addition, I felt that the first half of the paper (discovery that miR-1229-3p regulates synaptogenesis) and the second half (miR-1229-3p regulates mitochondrial function) is independent due to the lack of data linking them. It is important to propose a mechanistic explanation in which miR-1229-3p controls the tempo of human synaptogenesis "through" the regulation of mitochondrial function. To this end, the following issues need to be addressed.

Major concerns,
1. It is essential to document the expression of miR-1229-3p in various regions of the human brain during synapse formation, elimination, and maintenance, simply using publicly available data.
2. It is essential to show how igNeuron models the development of the human brain in vivo. I recommend a comparative transcriptome analysis to demonstrate the closeness of expression profiles between igNeurons and in vivo brain cells.
3. In the discussion, the genetic association between Alzheimer's disease and miR-1229-3p is briefly introduced, but it is worth showing the association with neurodevelopmental diseases that are causally related to synaptogenesis.
4. Can the phenotype of abnormal synaptogenesis in miR-1229-3p knockdown be rescued by improving mitochondrial function? In addition to Fig. 5g, a similar rescue experiment evaluating the synaptogenesis phenotype is needed.
5. Similarly, it would be interesting to perform simultaneous knockdown of miR-1229-3p and Pink1 and examine the number of synaptic puncta as in Fig3ef.
6. It is unclear whether synapse formation is really "slowed down" by miR-1229-3p. Knockdown experiments should be performed at more time points to verify whether the point of saturation of the synaptic growth curve (Fig3d) is prolonged without changing the peak/plateau level of the synaptic number.
7. Although the core sequences of miRNAs differ between humans and chimpanzees, their target sequences are highly conserved. Please interpret this. Was this miRNA really functional in the great apes? How conserved are they in great apes? More detailed documentation of the evolutionary process towards the status in modern humans is needed.

Minor concerns,
1. They found miRNAs conserved between mice and humans that are expressed during synaptogenesis only in humans (Fig2b). These are equally interesting as human-specific miRNAs. More functional studies for them?
2. In contrast to a weak phenotype on synaptic number (Fig3E), the changes in electrophysiological phenotypes such as EPSCs are relatively larger (Fig3i and j). Can this be explained in any way by gene expression changes in Fig4, such as the significant upregulation of channel genes but the modest change in the genes regulating synaptic formation?
3. In Fig3E and other plots. It is good to add statistical tests for the comparison in all possible pairs of conditions.
4. Which are the points of the four top DEGs (especially Pink1) in the volcano plot in Fig4a? It is good to indicate the plots of top-hit DEGs in a different color or mark.
5. Is the miR-1229-3p sequence in Neanderthals and Denisovans the same as in modern humans?
6. Is the miR-1229-3p sequence fixed in the human population when analyzing individual genomes of modern humans, such

as 1000 genome?

Reviewer #3

(Remarks to the Author)
The manuscript by Soutcheck et al. addresses an intriguing question: whether the evolutionary expansion of microRNAs contributes to the neoteny of human brain development. The authors formulate the hypothesis that human-specific microRNAs play a role in the prolonged maturation of neurons, specifically observed in humans.
To investigate this, they develop 2D cultures of human iPSC-derived cortical excitatory neurons in the absence of astrocytes (referred to as 'ig Neurons'). Subsequently, they conduct various assays, including the analysis of synapse number/function, transcriptome, and proteome analysis, aiming to correlate synapse maturation with these OMICS data. Their aim is to identify human-specific microRNAs associated with synapse maturation, leading them to detect miR-1229-3p. Inhibition of miR-1229-3p increases synapse number, impacts neuronal plasticity, and influences gene expression. One pathway that receives detailed analysis is mitochondrial function, with evidence provided that miR-1229-3p targets PINK1, affecting mitochondrial function.
In my opinion, this study is captivating, and the data presented are intriguing. However, the paper is somewhat challenging to digest, and the description of findings along with the data presentation in the figure panels could be enhanced for clarity. Unless I overlooked something—in which case, I apologize—it seems unfortunate that the raw or processed data from the various OMICS experiments are not included. Irrespective of the presented narrative, these data could be valuable for the field. I have a few specific suggestions that should be straightforward to address

1.
Fig. 1a shows igNeurons after 72 days of differentiation, while the analysis of synapses was only conducted up to day 40. It appears that the authors generated data for the 72 DIV time point; if so, this data should be included. Please provide a more detailed rationale for selecting different time points for analysis.
2.
Why was Ca+ imaging only performed until DIV 47?
3.
Fig S4 needs more explanation. What is meant by 1, 2, and 3... the 3 cells?
4.
Fig. S4d: This data might be important enough to be integrated into the main figure

5.
Fig 1f: Adding time points on top of the heatmap would enhance readability, especially as gray values aren't easily distinguishable.

6.
Fig S6A: It seems that the same color is used for day 0 and day 40.
7.
The transcript and protein changes during igNeuron maturation, as well as the examples of correlated and uncorrelated expression patterns, are not well explained in the text. This information, found within the figure legends of Fig S6, needs to be elaborated on in the main text.
8.
Given that igNeurons are presented as a novel model system, it would be beneficial to compare findings with previous datasets from 2-D cultures, organoids, and human brain tissue.
9.
Fig 1g: The word “methods” should be removed from the x-axis label. Additionally, it's unclear how the 25 proteins and 469 genes were selected from the gene-expression and proteomics datasets. The identity of these genes and proteins is unavailable, making it challenging to understand the synaptic clusters.

10.
The methods section lacks sufficient detail in various areas, and information on custom-made scripts is inconsistently provided. For example, while the parameters are given for mapping the transcriptomic data, info on other analysis tools is missing. The reporting summary refers to a Github link, but as far as I could see, info on the OMICS data and analysis is lacking. I suggest describing all bioinformatic analysis within the main text in detail at a level that will allow researchers to better understand and replicate the data.

11.
Data availability: The raw data from the transcriptome and protein analysis should be made available via common databases upon publication.

12.
Supplemental tables for all OMICS data (lncRNA, cirRNA, piRNAs, proteins, etc.) should be provided as the raw data is currently inaccessible.

13.

Fig 2A: The heatmap depicts 339 microRNAs, yet the text mentions both 339 and 181. Please clarify.

14.
Description of the data shown in Fig 2 and Fig S11 needs improvement in the main text. It's challenging to comprehend how the mouse data was generated and compared to ig Neuron data.

15.
The authors provide RNAscope images for miR-1229-3p. It would be interesting to describe if this microRNA has a synaptic localization synaptic mitochondria. Although the authors later focus on PINK1 it would be important to test if miR-1229-3p could target synaptic mRNAs. This could be done via bioinformatic approaches, since a number of suitable datasets on the synaptic RNAome and Proteome exist.

16.
Fig 4a. please provide the raw data and the list of diff. expressed genes as a supplementary table.

17.
Fig 17d. What are the genes up-regulated upon miR-1229-3p inhibition in igNeurons?

18.
Fig. 4. How many of the genes regulated by miR-1229-3p are changing during igNeuron development (Fig 1)?

19.
While the fact that miR-1229-3p seems to affect all mt-genes is interesting, I am not sure that its fair to say that “many” of the TOP15 GO terms were associated with mitochondrial function. Also please provide the entire list of significant GO terms as a supplementary table for all datasets.

20.
Does miR-1229-3p affect PINK1 protein levels?

21.
The authors mention a SNP within the miR-1229-3p gene that has been associated with Alzheimer’s disease. Does this SNP lead to loss of miR-1229-3p function and would it affect PINK1 levels? A simple approach would be to repeat the experiments shown in Fig. 3F if the SNP lies within the mature sequence of miR-1229-3p.

22.
It is interesting to see that miR-1229-3p als affects rat PINK1 when transfected into rat cortical neurons.

At the same time, there are several confirmed and predicted rodent microRNAs that can target PINK1.

Are any of these expressed in the igNeurons? If so, how is their expression pattern during differentiation?

Would the expression of a murine-specific microRNA that targets PINK1 in igNEurons affect mitochondria function and synapse development?

What is the expression pattern of Pink1 in igNeuron and rat neuronal development?

20.
As mentioned above it is sometimes very labboursome to follow the text, the figures and the legends.
Referring for example to Fig 1C, it's unclear what SC1, 2, and 3 stand for. While I assume they represent the three differentiations, 'sc' might also denote synapse clusters. Such ambiguities occur repeatedly throughout the manuscript and should be addressed for better understanding before publication.

The review process was also complicated since there are no page/line numbers.

Version 1:

Reviewer comments:

Reviewer #1

(Remarks to the Author)
Soutschek et al. have included new data in this revised manuscript that address the points that I have raised. Interestingly, these new data suggest that miR-1229-3p inhibition leads to lower mitochondrial activity and mitochondrial dysfunction, although not to the extent that mitophagy is increased, ultimately leading to altered Ca2+ regulation and dendritic arborisation. Despite this mitochondrial dysfunction, miR-1229-3p inhibition enhances synaptogenesis, and so this contrasts with Iwata et al. (ref. 32 in their manuscript) where it is reported that decreasing mitochondrial metabolism slows neuronal

development. How can the authors' findings be reconciled with Iwata et al.? Mito-ER interactions are considered in the discussion and indeed they mention that MERC regulation by miR-1229-3p could be a mechanism in human neurons contributing to slowing of mitochondrial metabolism and associated neuronal maturation. However, their findings don't appear to support the premise that impaired mitochondrial metabolism slows neuronal maturation. I would be interested in additional discussion on this point.

Reviewer #2

(Remarks to the Author)
The authors have sincerely addressed all reviewer comments, and the revised manuscript is considered to be of a much higher quality compared to the initial submission. While they have not performed a specific experiment for every point I suggested, their explanations for not doing so are convincing, which I find acceptable. The current version of the manuscript is suitable for publication and does not have any major issues.

Reviewer #3

(Remarks to the Author)
The authors have addressed all concerns sufficiently. Congratulations.

Open Access This Peer Review File is licensed under a Creative Commons Attribution 4.0 International License, which permits use, sharing, adaptation, distribution and reproduction in any medium or format, as long as you give appropriate credit to the original author(s) and the source, provide a link to the Creative Commons license, and indicate if changes were made.
In cases where reviewers are anonymous, credit should be given to 'Anonymous Referee' and the source.
The images or other third party material in this Peer Review File are included in the article's Creative Commons license, unless indicated otherwise in a credit line to the material. If material is not included in the article's Creative Commons license and your intended use is not permitted by statutory regulation or exceeds the permitted use, you will need to obtain permission directly from the copyright holder.
To view a copy of this license, visit https://creativecommons.org/licenses/by/4.0/

Dear Professor Schratt,

Thank you again for submitting your manuscript "A human-specific microRNA controls the timing of excitatory synaptogenesis" to Nature Communications. Again, we are sorry for the delay. We have now received reports from 3 reviewers. I'm happy to inform you that we found unanimous enthusiasm and interest in your study amongst the reviewers.

However, as you will see from the reports copied below, the reviewers raise important concerns. We find that these concerns limit the strength of the study, and so would invite major revisions of the manuscript.

In particular, we would want to see orthogonal data/approaches addressing the physiological relevance of the findings with published datasets--potentially showing miR-1229-3p expression in human tissue and/or comparing igNeuron data with previous in vivo/in vitro datasets. We would be keen to see further insights into miR-1229-3p and Pink1 interaction, as well as further support for miR-1229-3p in the regulation of mitochondria function. While highlighting these issues, we are not implying that the other concerns raised by the reviewers are less important. And we are open to the authors interpretation for how best to handle the reviewers' feedback; but do feel without substantial revisions, we will be unlikely to send the paper back to review.

I am more than happy to consult further, should you find it useful.

If you feel that you are able to comprehensively address the reviewers' concerns, please provide a point-by-point response to these comments along with your revision. Please show all changes in the manuscript text file with track changes or colour highlighting. If you are unable to address specific reviewer requests or find any points invalid, please explain why in the point-by-point response.

Important: In addition to the above, you must comply with the following editorial requests; we will not be able to proceed with your revised manuscript otherwise. Please also see the *Nature Communications* formatting instructions, which you may find useful while preparing your revised manuscript.

REVIEWER COMMENTS

General:

We want to thank the reviewers for the overall positive feedback and for the valuable suggestions to further improve our manuscript. Based on this feedback, we have compared our in vitro model with published in vitro/in vivo datasets and thereby obtained strong support for expression of miR-1229-3p in human post-mortem brain tissue as well as for the comparability of our igNeuron in vitro model to previously published protocols regarding the developmental timing. In addition, we revisited the effects of miR-1229-3p on mitochondria with several new readouts (mitoKeima for mitophagy, Rhod2 for mitochondrial calcium, TMRE for MMP, Sybr Gold for mitochondrial DNA). This extensive characterization surprisingly revealed that mitochondria, contrary to our initial hypothesis, become progressively dysfunctional upon miR-1229-3p inhibition over the course of human neuronal development. Due to this new data, we also revisited the potential involvement of the direct miR-1229-3p target PINK1 in synaptogenesis. Using a new tool to directly block the miR-1229-3p/PINK1 interaction, we however could not detect any significant alterations in synapse density compared to control conditions, arguing that upregulation of PINK1 upon miR-1229-3p inhibition is not sufficient to cause detectable alterations in human neuron development. To obtain additional mechanistic insight, we therefore added an entirely new set of experiments (new Fig. 6) whereby we analyzed changes in gene expression upon an acute miR-1229-3p overexpression in SH-Sy5y cells, with the goal to identify additional genes directly repressed by miR-1229-3p. Thereby, we identified not only a large group of putative miR-1229-3p target genes involved in mitochondria/ER biology but also several genes directly implicated in neuronal and synapse development. Considering the prominent observed mitochondrial phenotypes, we went on to validate Synj2bp as a direct miR-1229-3p target. Synj2bp is a protein involved in the regulation of mitochondrial-ER contact sites and local translation at mitochondria. Based on this extensive new data, we present an updated model whereby miR-1229-3p is required for mitochondrial homeostasis during human neuron development by controlling an entire network of genes at the mitochondria-ER interface. In that sense, perturbed mitochondrial/ER homeostasis initially induces compensatory (beneficial) effects, such as increased synaptogenesis, whereas prolonged mitochondrial dysfunction leads to impaired neuronal maturation and calcium buffering.

Reviewer #1 (Remarks to the Author):

Thanks to the editors for inviting me to comment on this manuscript.

Soutschek et al. present a study that explores the contribution that non-coding genes might make to the relative slowing of human neuronal maturation (neoteny). Using a glial free human iPSC-derived neuronal model (igNeurons), they present data showing widespread expression changes of non-coding RNAs, including microRNAs, during excitatory synapse development. They focus on a human-specific microRNA, miR-1229-3p, which specifically correlates with neuronal (and thus likely synaptic) maturation in this system. Inhibition of this particular microRNA appears to accelerate neuronal maturation, enhance Ca2+ transients and increase synaptic transmission. This potentially works via regulation of PINK1. Boosting mitochondrial metabolism reduces Ca2+ spiking activity, partially rescuing the impact of inhibition of miR-1229-3p.

The rationale for focusing on miR-1229-3p appears solid, since (as the authors show) its sequence (and secondary structure) is not conserved in other species and its expression tracks with synaptogenesis. Furthermore, human (but not chimp equivalent) miR-1229 mirtron can downregulate miR-1229-3p expression, inferred via a luciferase binding site reporter assay.

The authors assess the impact of miR-1229-3p KD by applying ASOs in igNeuron cultures from d9.

Synaptic numbers (and sEPSC charge transfer) are increased at d21 but not d33, whereas there is decreased dendritic complexity at the later time point. Nevertheless, the effect sizes here are relatively modest. Transcriptomics reveals many DEGs associated with mitochondrial function, including downregulation of mtDNA encoded genes, and Pink1 upregulation. They then show that Pink1 is a direct target of miR-1229-3p. Human neurons treated with pLNA-1229 (hence derepressed Pink1) have mitochondria with disturbed MMP, more colocalisation with LAMP2 (suggesting mitophagosomes, the authors claim), elevated fragmentation (suggesting enhanced mitophagy, the authors claim), higher numbers of axonal mitochondria in pLNA-1229 condition (using mitotracker signal), increased lactate concentration in media (suggesting shift to glycolysis) and increased Ca2+ signals. Increasing mitochondrial metabolism via GSK-2837808A and AlbuMAX restored normal Ca2+ signal amplitude.

Overall, I found this to be a very interesting study. Many elements of the manuscript are very solid and well explained, and the findings are of significance to the field.

We thank the reviewer for this general positive assessment of our study and the statement on its significance!

I have some general comments:

1. For me, to really clinch it would be to apply this miRNA to mouse neurons, and see a delay in synapse development and maturation, via Pink1 activation. Has this been attempted?

While the application of miR-1229-3p to rat or mouse primary neurons is in principle possible, the 3'UTRs of the vast majority of mRNAs is not conserved between humans and mice, e.g. the Pink1 and Synj2bp 3'UTRs, and hence the observed phenotypic effects are most likely completely unrelated. To highlight the specificity of 3'UTRs and corresponding miR-1229-3p binding sites, we included a correlation of miRNA repression predictions of putative miR-1229 binding sites on mouse 3'UTRs in comparison to the ones predicted in humans in the revised version of the manuscript (Suppl. Fig. 15).

2. Since effect sizes are quite modest, in my opinion the authors' claim "that miR-1229 depletion leads to *pronounced* morphological and functional changes during human excitatory synapse development" is perhaps overstated.

We agree with the reviewer and changed the respective wording in the abstract and main text of the manuscript.

Also, I have some queries regarding the data shown in Fig. 5:

1. In Fig. 5a, c, d, Mitotracker intensity overall looks altered in pLNA-1229 neurons compared to controls, possibly reflecting altered MMP. Is MMP altered in this condition?

Could decreased MMP be reducing mitochondrial Ca2+ uptake, accounting for why cytosolic Ca2+ buffering is impaired?

We thank thank the reviewer for this suggestion, we have now further assessed the membrane potential (MMP) of mitochondria in pLNA-1229 compared to control-treated neurons. For measurement of MMP, we have decided to use tetramethylrhodamine ethyl ester (TMRE), rather than Mitotracker intensity, since this has been shown to be a more accurate measure for MMP (Mitotracker binds covalently post-uptake into mitochondria and is thus even retained after depolarization). Using TMRE measurements, we did not observe significantly altered MMP (Suppl.

Fig. S25), suggesting that elevations in Mitotracker intensity and impaired Ca-buffering are not due to a compromised MMP. To avoid misleading conclusions, we have decided to removeexperiments using Mitotracker from the revised manuscript and only present data about mitochondrial morphology obtained with SIM live-cell imaging using a dye which is insensitive to alterations in MMP (new Fig. 5).

The authors could test directly whether mitochondrial Ca2+ uptake is impaired in pLNA-1229 by using a fluorescent reporter of mitochondrial Ca2+ (e.g. mito-GCaMP or similar).

We thank this reviewer for their suggestion and now measured mitochondrial Ca2+ levels directly at Day 25 using a fluorescent Ca2+ sensor (Rhod2), which is preferentially located in mitochondria with the employed staining conditions. We observed a significant reduction in mitochondrial matrix Ca2+ levels in pLNA-1229-3p compared to control-transfected human iNeurons (Fig. 5j-l). These findings are consistent with impaired mitochondrial Ca2+ homeostasis, which in turn could result in elevated cytosolic Ca2+ levels.

2. What does "mitochondrial entropy" mean exactly in 5d, and how is this calculated? These points were not clear to me, from the information available.

Entropy serves here as a measurement of the univariance of the Mitotracker signal in the soma and is a direct output of ourCellProfiler pipeline to quantify the Mitotracker signal. We initially choose this measurement to underline the visual appearance of an inhomogeneous mitochondrial signal particularly in the soma of neurons treated with the pLNA-1229. However, as described above, we have now decided to remove all data obtained with Mitotracker stainings from the revised manuscript.

3. For the images shown in Fig 5b, please can each channel be displayed separately? Co-localisation of Mitotracker puncta with LAMP2 should be quantified in pLNA-1229 neurons and controls.

We included a more thorough analysis of mitophagy in human neurons treated with pLNA-1229 in the revised manuscript using mtKeima probes (see reply to point 4). Since this is a more direct measurement of mitophagy, we removed the Lamp2 colocalization panel from the revised manuscript. See our comment on point 4 for more details.

4. To support their claim of increased mitophagy, a reporter of acidified mitochondria (e.g. mito-QC, mito-SRAI, mito-Keima) could be used to demonstrate incorporation of mitochondria into an acidified compartment (e.g. mitophagosome), and/or co-labelling with parkin to demonstrate parkin recruitment to mitochondria.

We agree with the reviewer that our claim of elevated mitophagy needed to be more substantiated. In that regard, we added to the revised version of the manuscript an experiment where we infected induced human neurons with a mito-Keima lentiviral construct and analyzed mitophagy in neurons treated with the pLNA-Ctrl and pLNA-1229. Contrary to our initial hypothesis, we observed a significant reduction of mitochondria incorporated into an acidified compartment, indicative of reduced mitophagy, upon pLNA-1229 treatment (new Fig. 5d).

Our surprising finding of reduced mitophagy in pLNA-1229 treated neurons also prompted further experiments addressing the fate of mitochondrial DNA (mtDNA), since mitophagy has been shown to

reduce the amount of cytosolic mtDNA release and prevent downstream inflammatory responses (Ma et al., Front. Cell Dev. Biol. 2020). Consistent with these previous reports, we found that reduced mitophagy correlated with increased cytosolic mtDNA in pLNA-1229-3p treated human neurons (Fig. 5a-c). Furthermore, our results are also in agreement with our new Rhod2 measurements, which show reduced mitochondrial calcium concentration in pLNA1229 neurons (Fig. 5j-l), since it has been previously shown that mitochondrial calcium signaling and mitophagy mutually regulate each other (Borbolis et al., NPJ Met. Health and Disease, 2025). Together, our data indicates that mitochondrial morphology and physiology gets progressively altered over the course of differentiation in pLNA-1229-treated neurons, but do not (yet) enter a mitophagy state at the measured time point. Interestingly, the altered mitochondrial Ca2+ signaling and the results from the miR-1229 overexpression sequencing pinpoint to an impairment of mitochondrial ER contact sites, in line with increased ER stress signaling. It has been previously shown that ER stress, particularly in the context of neurodegenerative diseases, inhibits rather than promotes autophagy (Rashid et al., Autophagy, 2015). Increased synaptogenesis at an early stage of differentiation therefore likely represents an adaptive response to mild mitochondrial stress and ER stress, i.e., metabolic activation (Carreras-Sureda et al., Science Signaling, 2022), which has been shown to promote neuronal development (Iwata et al., Science, 2023).

5. In Fig. 5f and g, I would suggest imaging mitochondria with a non-MMP dependent marker (e.g. mitoDsRed or similar) to quantify mitochondria in axons to avoid the potential confounder of mitochondria with depleted MMP (perhaps more likely to be present in the pLNA-1229 neurons) not being visualised with a dye that is MMP dependent.

To measure mitochondrial length in the previous figures Fig. 5f and g, we used the mitochondrial dye Pk Mito Orange that is not membrane potential dependent (Liu et al., 2022, PNAS). According to the suggestion of this reviewer and to some discrepancies between data obtained with Mitotracker and non-MMP dependent dyes (cf. Figs 5), we decided to remove the Mitotracker data entirely from the revised manuscript.

6. In Fig. 5j it would be helpful to see some representative micrographs of the Ca2+ imaging, as well as quantification.

We include representative snapshots of the time-lapse videos recorded for the Ca-Amplitude analysis in the revised version of the manuscript. In addition, we also included a quantification of spike frequency in the the revised version of the manuscript. Strikingly, the frequency of cytosolic calcium spikes could be likewise rescued by activation of mitochondrial respiration (Fig. 5m)

7. How do the authors propose that mitochondrial activation (with Albumax or the LDHA inhibitor) leads to increased cytoplasmic Ca2+ buffering? Do they observe an increase in mitochondrial mass, MMP, change in morphology, and/or increased interaction with ER, when these treatments are administered?

Although we have no direct experimental evidence, it has been previously shown that elevated mitochondrial calcium concentration promotes mitochondrial respiration and ATP production (McCormack et al., Phys. Reviews, 1990; Lee et al., Bioch. Soc. Trans., 2023) and that ATP in turn promotes mitochondrial Ca2+ buffering (Nichols & Scott, Bioch. J., 1980). In this regard, we speculate that the regulation of mitochondria/ER contact sites by miR-1229-3p could play an important role by affecting the transfer of calcium between mitochondria and the ER. Further, by activating

mitochondrial metabolism with Albumax and GSK-2837808A, we increase mitochondrial respiration and Ca2+ buffering. . This is now discussed in the revised manuscript. Investigating the detailed mechanisms underlying increased Ca2+ buffering in response to mitochondrial activation is in our opinion beyond the scope of the current manuscript.

Reviewer #2 (Remarks to the Author):

Soutschek et al. established an experimental system that allows human neurons to form synapses in a culture environment without feeder astrocytes, and by effectively using this system, showed that miR-1229-3p, miRNAs with human-specific sequences, can slow down excitatory synapse formation. The regulation of gene expression by this miRNA alters mitochondrial function and may thus control the timing of neuronal maturation, typically synaptogenesis. This is consistent with the results of several recent papers showing that mitochondria-related metabolic control regulates the rate of developmental progression, and in particular with a recent paper (Iwata et al. Science) showing that mitochondria regulate the rate of human neuronal maturation. The evolutionary mechanisms of human brain development are a topic of interest to many researchers, and the finding in this manuscript that human-specific miRNA may be relevant to the protracted neuronal maturation uniquely observed in humans is intriguing.

On the other hand, the paper has obvious shortcomings. All experiments were performed in igNeuron, an artificial experimental system where synapse formation can be observed dramatically earlier than in vivo by introducing the Ngn2 gene into iPS cells, and there is no evidence that miR-1229-3p actually works under human physiological conditions. Therefore, revisions with additional analyses bridging the results of in vitro experiments and in vivo physiology will significantly increase the impact of the manuscript. In addition, I felt that the first half of the paper (discovery that miR-1229-3p regulates synaptogenesis) and the second half (miR-1229-3p regulates mitochondrial function) is independent due to the lack of data linking them. It is important to propose a mechanistic explanation in which miR-1229-3p controls the tempo of human synaptogenesis "through" the regulation of mitochondrial function. To this end, the following issues need to be addressed.

Major concerns,
1. It is essential to document the expression of miR-1229-3p in various regions of the human brain during synapse formation, elimination, and maintenance, simply using publicly available data.

The availability of microRNA expression data during human brain development is still relatively scarce, since most omics studies have so far focused on the measurement of long RNAs (especially mRNAs) and proteins. One of the most comprehensive studies is done by Sousa et al. (Science, 2017), where the authors profiled miRNA expression from various brain regions in post-mortem samples obtained from human, chimpanzee and macaque. In the revised manuscript, we included an analysis of miR-1229-3p expression over various brain regions in these three species and compared its abundance with a conserved microRNA (miR-134-5p) (new Fig. 2d and Suppl. Fig. S13). These results demonstrate that miR-1229-3p is expressed in various regions in the human brain. Another resource for miRNA expression in human tissues is the miRNA tissue atlas (https://ccb-compute2.cs.uni-saarland.de/mirnatissueatlas_2025). By querying this database, we found high expression of miR-1229-3p in human brain tissue, ranking 5th among all organs present in the database. Finally, in collaboration with the group of B. Elfving (Aarhus), we performed qPCR analysis with RNA obtained from post-mortem human brain tissue of psychiatric patients. Thereby, we observed robust expression of miR-1229-3p, also in comparison to other more broadly conserved microRNAs such as

miR-146a-5p, miR-485-5p or miR-210-3p (Suppl. Fig. S14). In conclusion, both database searches and own experimental data strongly support robust miR-1229-3p expression in various human brain regions. Knowledge about the developmental trajectory will require more extensive collection of human tissue samples across development.

2. It is essential to show how igNeuron models the development of the human brain in vivo. I recommend a comparative transcriptome analysis to demonstrate the closeness of expression profiles between igNeurons and in vivo brain cells.

We have now performed a comparative transcriptome analysis of our igNeuron data with published RNA-seq datasets (Lin et al., Stem Cell Rep. 2021; Trevino et al., Cell 2021 & Mayer et al., Neuron, 2017) (new Fig. 1g and Suppl. Fig. S8). Thereby, we observed strong correlations of the expression profiles of specific developmental time points of our igNeuron model with corresponding stages of human in vivo development, for example with glutamatergic neurons between stage 16-24 in the Trevino et al. dataset. This data thereby confirms that our reductionist in vitro differentiation model is suitable to model at least some important aspects of human in vivo neuronal maturation.

3. In the discussion, the genetic association between Alzheimer's disease and miR-1229-3p is briefly introduced, but it is worth showing the association with neurodevelopmental diseases that are causally related to synaptogenesis.

To our knowledge, there are to date no published associations between miR-1229-3p variants and neurodevelopmental diseases. This, however, might be simply because most studies until recently focused on variations in protein-coding genes and did not further investigate SNPs occurring in non-coding DNA regions.

4. Can the phenotype of abnormal synaptogenesis in miR-1229-3p knockdown be rescued by improving mitochondrial function? In addition to Fig. 5g, a similar rescue experiment evaluating the synaptogenesis phenotype is needed.

According to our new data on mitochondrial function in pLNA-1229-3p treated neurons, we propose that miR-1229-3p knockdown leads to a decline in mitochondrial activity over the course of differentiation, which manifests in impaired Ca2+ buffering and dendritic arborization (Fig. 5). In contrast, the early synaptogenesis phenotype is likely associated with a temporary increase in (mitochondrial) processes counteracting stress, such as the ER stress and unfolded protein response (UPR), both of which have been shown to have metabolic and synaptogenesis-promoting effects (Carreras-Sureda et al., Science Signaling, 2022, i.e. Godin et al., Trends in Neuro., 2016). Thus, improving mitochondrial function is unlikely to rescue the premature synaptogenesis phenotype in miR-1229-3p knockdown neurons, and we therefore decided not to perform the suggested experiment. Beyond the scope of this manuscript, it would be important to identify the specific mechanism(s) which trigger premature synaptogenesis. This would allow to devise specific pharmacological or genetic strategies for rescue experiments, which are needed to demonstrate a causal role in the process.

5. Similarly, it would be interesting to perform simultaneous knockdown of miR-1229-3p and Pink1 and examine the number of synaptic puncta as in Fig3ef.

As suggested by this reviewer, we have now attempted to test the impact of perturbing the putative miR-1229-3p – Pink1 interaction on synaptic puncta in human igNeurons. Instead of performing a simultaneous knockdown of both molecules, which is difficult to achieve in human neurons, we decided to block the miR-1229-3p binding motif within the Pink1 3UTR using a target-site blocking oligonucleotide (TSB). This approach is supposed to relieve specifically Pink1 from miR-1229-3p repression, without affecting other target genes. As a result, if upregulation of Pink1 is required for premature synaptogenesis, we would expect that, similar to miR-1229-3p knockdown, the Pink1 TSB should lead to increased formation of synaptic co-clusters compared to control-treated neurons. Reduced repression of the Pink1 3'UTR when applying the Pink1 TSB could be validated in HEK cells, using our miR-1229-3p reporter gene system (not shown).. However, Pink1 TSB had no effect on synapse co-cluster density during human igNeuron development (see below). Therefore, we conclude that regulation of Pink1 is not sufficient to explain the effect of miR-1229-3p knockdown on synaptogenesis. This result is also in agreement with our new data from miR-1229-3p overexpression in SH-Sy5y cells (Fig. 6), where we didn't observe a significant downregulation of Pink1 mRNA levels in miR-1229-3p overexpressing cells based on RNA-seq. Our results thereforesuggest that miR-1229-3p controls the expression of an entire network of genes coordinating both synapse and mitochondrial function (new Fig. 6). Manipulating the expression of a single gene within these networks is unlikely to recapitulate the full spectrum of phenotypes observed upon miR-1229-3p knockdown. We have now extensively discussed the potential role of miR-1229-3p regulated gene networks in mitochondria and synapse homeostasis in the revised manuscript.

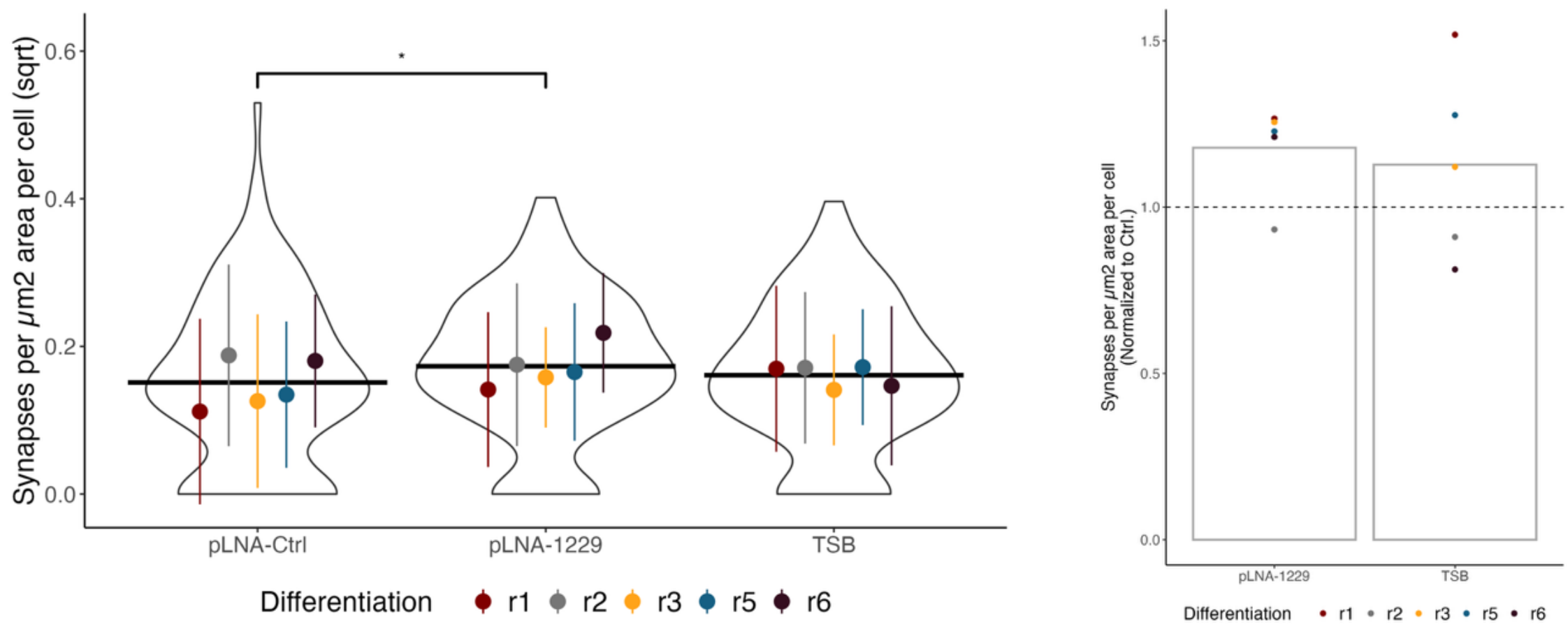

Synapse co-cluster density of five independent differentiations upon applying the pLNA-1229 and a Pink1 miR-1229 target site blocker (TSB). Violin plots of all datapoints together with the mean and standard deviation of each differentiation are shown on the left, the normalized ratio of each differentiation (compared to the pLNA-Ctrl) on the right. Statistics were performed using a linear model accounting for the individual differentiations as fixed effects.

6. It is unclear whether synapse formation is really "slowed down" by miR-1229-3p. Knockdown experiments should be performed at more time points to verify whether the point of saturation of the synaptic growth curve (Fig3d) is prolonged without changing the peak/plateau level of the synaptic number.

We agree with this reviewer that making conclusive statements about a role of miR-1229-3p in the timing of synapse formation would require a much more extensive characterization of synaptic co-clusters at multiple time points along the course of differentiation. However, we want to stress that the determination of synaptic parameters in human igNeurons is an extremely time- and labor-intensive process, involving several weeks of imaging followed by image analysis for each individual time point. Since the editor and other reviewers were asking for a detailed characterization of the "mitochondrial phenotype", which also required significant imaging time, we were unable to perform in parallel additional time points for the synaptogenesis time course. Since we acknowledge this weakness, we toned down our statements regarding the regulation of developmental timing and rather discussed the dichotomy of synaptic and dendritic phenotypes caused by miR-1229-3p inactivation.

7. Although the core sequences of miRNAs differ between humans and chimpanzees, their target sequences are highly conserved. Please interpret this. Was this miRNA really functional in the great apes?

Due to its structural conformation, we suggest that there is no mature miR-1229 generated in the great apes. This is further supported by the luciferase experiment shown in Fig. 2i). Coming from an evolutionary perspective, it might make more sense to evolve certain nucleotides in a microRNA in comparison to individual target site nucleotides.

How conserved are they in great apes?

Please see our conservation analysis in Fig. 2).

More detailed documentation of the evolutionary process towards the status in modern humans is needed.

We carefully inspected available genomic information from Neandertal and Denisovan sequencing experiments aligned to the human hg19 genome. It appears that there is a lack of sequencing information (and reads) at the specific loci of miR-1229 and hence a more detailed statement regarding the evolutionary development towards modern humans is currently impossible to make. Please find below alignments of the Neandertal and Denisovan sequence reads to the human genome, downloaded from the USCS genome browser.

Neandertal:

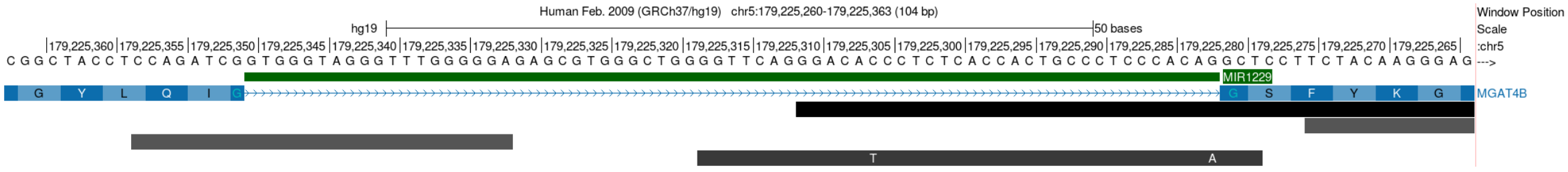

Denisovan:

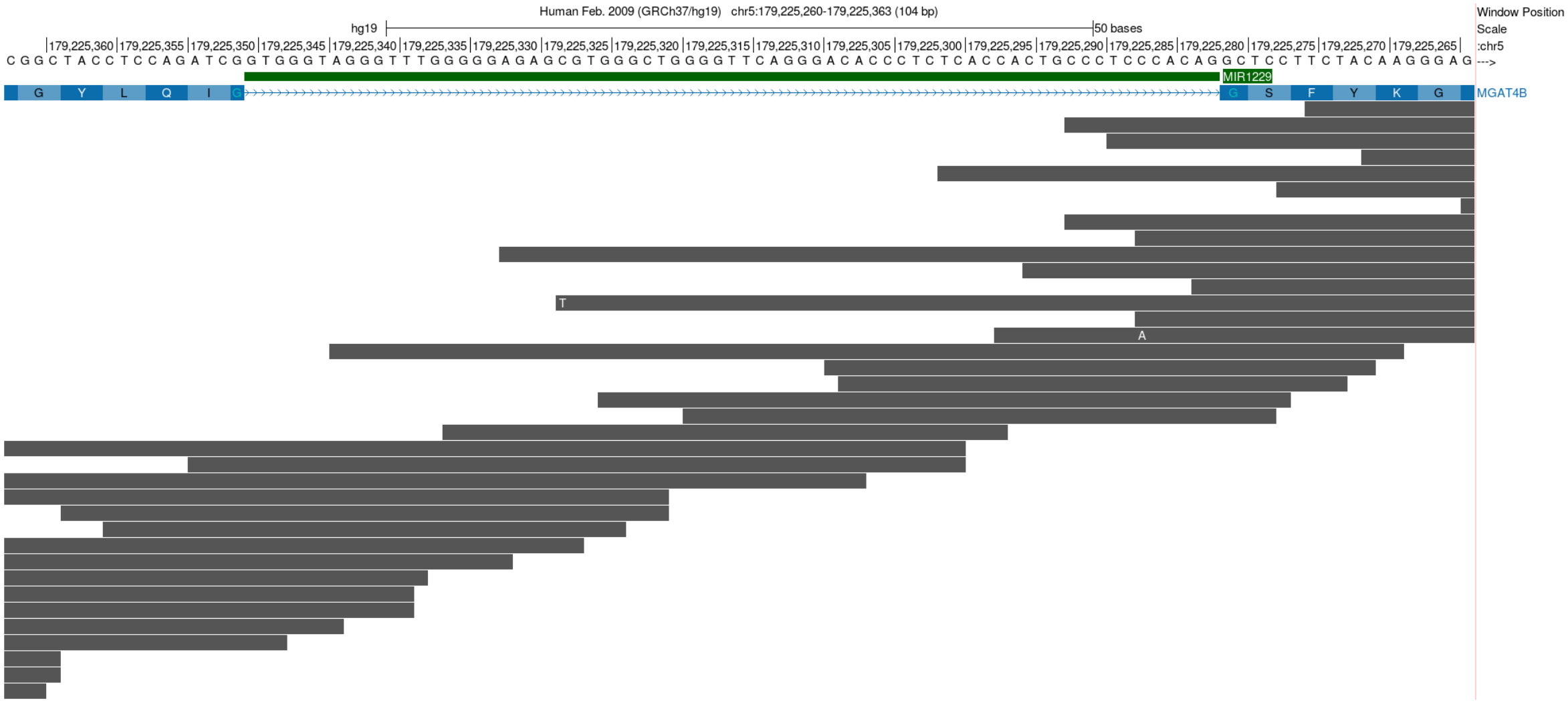

Minor concerns,

1. They found miRNAs conserved between mice and humans that are expressed during synaptogenesis only in humans (Fig2b). These are equally interesting as human-specific miRNAs. More functional studies for them?

Indeed, our analysis revealed several additional miRNAs which are differentially expressed between human and mouse neurons. However, for this initial characterization, we focused on a truly human-specific candidate, miR-1229-3p, as the most promising candidate for controlling human-specific traits. Other interesting candidates will be followed up in future studies but are in our opinion beyond the scope of the present manuscript. Therefore, we have moved the corresponding figure (Fig. 2b) to the supplement of the revised manuscript.

2. In contrast to a weak phenotype on synaptic number (Fig3E), the changes in electrophysiological phenotypes such as EPSCs are relatively larger (Fig3i and j). Can this be explained in any way by gene expression changes in Fig4, such as the significant upregulation of channel genes but the modest change in the genes regulating synaptic formation?

We agree with the observation that effects on EPSCs appear larger compared to effects on synapse number upon miR-1229-3p inhibition. However, we want to remind that the RNA-seq was performed at an early time point (day 21) of the differentiation concomitant with the synapse phenotype, while elctrophysiological (ephys) recordings were doneat later stages (day 40-42). Therefore, it is difficult to directly link gene expression changes to the ephys results. One possible explanation for the strong ephys phenotypes at later time points, which correlate with a marked increase in cytosolic Ca2+ concentrations, is the apparent progressive mitochondrial dysfunction (e.g., mitochondrial fragmentation, impaired Ca2+ buffering, switch to glycolysis) upon prolonged miR-1229-3p inhibition (see also our comments to reviewer 1).

3. In Fig3E and other plots. It is good to add statistical tests for the comparison in all possible pairs of conditions.

We conducted statistical tests for all pairs, however, show only significant comparisons of inhibited miRNAs versus the control (Ctrl.)condition. For further details please consult the Github repository.

4. Which are the points of the four top DEGs (especially Pink1) in the volcano plot in Fig4a? It is good to indicate the plots of top-hit DEGs in a different color or mark.

We now moved a heatmap with the most significant gene expression changes upon miR-1229 inhibition from the supplement to the main manuscript (Fig. 4b). In addition, we would like to mention that our new data from miR-1229-3p overexpression in SH-Sy5y cells argues that genes upregulated in the pLNA-sequencing (such as Pink1)are not the ones most strongly regulated by miR-1229-3p. In fact, the long period of miR-1229-3p inhibition in igNeurons (up to 2weeks) might cause many secondary effects due to alterations in synapse and mitochondrial biology, which obscure the direct effects of miR-1229-3p inhibition. This could explain why we see more genes being significantly downregulated upon the inhibition of miR-1229-3p (opposite to what one would expect with a microRNA knockdown) and why the miR-1229-3p binding motif, in contrast to the miR-1229-3p overexpression experiment in SH-Sy5y cells (Fig. 6) is not significantly enriched in differentially expressed genes in igNeurons (Suppl. Fig. S21b). While some of the initially identified mitophagy-associated genes, such as Pink1, might still be under direct control of miR-1229-3p (initial Fig. 4e), we currently do not have any causal evidence for their involvement in the observed mitochondrial or synaptic alterations. Therefore, we did not highlight these particular genes in the mentioned volcano plot. Instead, we now provide a figure wherein we describe an entire gene network which likely controls mitochondrial and synaptic function downstream of miR-1229-3p based on results from miR-1229-3p overexpression in SH-SY5Y cells (Fig. 6).

5. Is the miR-1229-3p sequence in Neanderthals and Denisovans the same as in modern humans?

See above

6. Is the miR-1229-3p sequence fixed in the human population when analyzing individual genomes of modern humans, such as 1000 genome?

We thank this reviewer for their suggestion. Interestingly, it appears that the SNP rs2291418, which has been previously associated with Alzheimer's disease, is present at almost 3% among modern humans according the 1000genome project and ALFA aggregated frequencies. We included two new figures describing the alternative allele frequencies of this SNP in individual populations in the new Suppl Fig. S32

Reviewer #3 (Remarks to the Author):

The manuscript by Soutcheck et al. addresses an intriguing question: whether the evolutionary expansion of microRNAs contributes to the neoteny of human brain development. The authors formulate the hypothesis that human-specific microRNAs play a role in the prolonged maturation of

neurons, specifically observed in humans.
To investigate this, they develop 2D cultures of human iPSC-derived cortical excitatory neurons in the absence of astrocytes (referred to as 'ig Neurons'). Subsequently, they conduct various assays, including the analysis of synapse number/function, transcriptome, and proteome analysis, aiming to correlate synapse maturation with these OMICS data. Their aim is to identify human-specific microRNAs associated with synapse maturation, leading them to detect miR-1229-3p. Inhibition of miR-1229-3p increases synapse number, impacts neuronal plasticity, and influences gene expression. One pathway that receives detailed analysis is mitochondrial function, with evidence provided that miR-1229-3p targets PINK1, affecting mitochondrial function.
In my opinion, this study is captivating, and the data presented are intriguing. However, the paper is somewhat challenging to digest, and the description of findings along with the data presentation in the figure panels could be enhanced for clarity. Unless I overlooked something—in which case, I apologize—it seems unfortunate that the raw or processed data from the various OMICS experiments are not included. Irrespective of the presented narrative, these data could be valuable for the field. I have a few specific suggestions that should be straightforward to address

We thank the reviewer for the positive perception of our study and emphasizing that it is “captivating”.

Please note that we did upload the raw RNA sequencing data to the GEO repository (including processed tables) and the proteomics data to the PRIDE database. Until the paper is published, the raw data can though only be accessed by reviewers with tokens mentioned in the data availability sections. We highlighted the availability of this data now further also in the main text (not only at the end) and refer to the Web Application (containing all analyzed and processed data of the time course experiment) not only in the legend of figure 1 but also in the main text. Additionally, we would like to highlight that we reanalyzed the proteomics dataset with a newer version of the Spectronaut software, now revealing over 6400 proteins (5280 sign. changing), instead of the roughly 3300 proteins that we had detected before.

1.
Fig. 1a shows igNeurons after 72 days of differentiation, while the analysis of synapses was only conducted up to day 40. It appears that the authors generated data for the 72 DIV time point; if so, this data should be included. Please provide a more detailed rationale for selecting different time points for analysis.

The image shown at day 72 is an example image for the robustness of our protocol. We have not conducted the synapse analysis beyond day 40. Different time points were selected dependent on the specific readouts. For example, for ephys experiments, we chose later time points in the differentiation (beyond day 30) since these recordings require that synapses have already sufficiently matured. On the other hand, earlier time points (e.g. day 21) were chosen since synapse density at this stage is still increasing before reaching a plateau phase from day 27 on (Fig. 1c). For detailed follow-up experiments we mostly chose this early time pointsince no effects of miR-1229 inhibition on synapse density were observed beyond day 21.

2.
Why was Ca+ imaging only performed until DIV 47?

Since these imaging experiments were all performed from the same differentiations, keeping coverslips for more days was technically challenging. Further, we observed that both frequency and

amplitude seem to start plateauing following 33 days of culturing igNeurons (as well as that the variability between coverslips became larger with increasing days). Therefore, we decided to only include time points until day 47.

3.
Fig S4 needs more explanation. What is meant by 1, 2, and 3... the 3 cells?

Yes, these are indeed traces of individual cells. We explained the panels of this figure more carefully in the legend and refer readers for more information to the web page of the calcium analysis app.

4.
Fig. S4d: This data might be important enough to be integrated into the main figure

Since this panel represents only a qualitative example trace of action potentials recorded by the patch-clamp technique without further quantification, we would prefer to leave it in the supplement.

5.
Fig 1f: Adding time points on top of the heatmap would enhance readability, especially as gray values aren't easily distinguishable.

We added an annotation of the individual days below each column in Fig. 1f.

6.
Fig S6A: It seems that the same color is used for day 0 and day 40.

We chose more distinguishable colors for the three time-course PCA plots.

7.
The transcript and protein changes during igNeuron maturation, as well as the examples of correlated and uncorrelated expression patterns, are not well explained in the text. This information, found within the figure legends of Fig S6, needs to be elaborated on in the main text.

We included a more thorough explanation of the molecular development of igNeurons in the main text, specifically referring to the pluripotency and astrocytic markers mentioned in Fig. S6.

8.
Given that igNeurons are presented as a novel model system, it would be beneficial to compare findings with previous datasets from 2-D cultures, organoids, and human brain tissue.

Please refer to our comment to reviewer 2 regarding this point.

9.
Fig 1g: The word “methods” should be removed from the x-axis label. Additionally, it's unclear how the 25 proteins and 469 genes were selected from the gene-expression and proteomics datasets. The identity of these genes and proteins is unavailable, making it challenging to understand the synaptic clusters.

We thank the reviewer for this remark and removed the word as methods as well as clarified in the corresponding figure legend. For a more detailed description (than what has been explained in figure S6 and the methods) as well as the exact analysis and gene lists, we refer to the published Github repository (particularly the following script:
01_TimeCourse_MultiOmics/Figures_Omics/SmoothMeans_Cluster9/Cluster9_Overlap.Rmd. Here we describe the plotting and make the individual gene lists available.The script where we particularly clustered time course ribosomal depletion dataset to obtain these genes can be found in
01_TimeCourse_MultiOmics/RNA_GRCh38.p10_Ens91/Long_RNA_R/longRNA_Clustering.Rmd.In addition, we provide in the revised manuscript a supplementary table (S1) containing the identity of the referred genes and proteins. We refer to this table in the figure legend.

10.
The methods section lacks sufficient detail in various areas, and information on custom-made scripts is inconsistently provided. For example, while the parameters are given for mapping the transcriptomic data, info on other analysis tools is missing. The reporting summary refers to a Github link, but as far as I could see, info on the OMICS data and analysis is lacking. I suggest describing all bioinformatic analysis within the main text in detail at a level that will allow researchers to better understand and replicate the data.

We again refer to the public Github repository which contains all the relevant scripts and parameters that are not explained in the methods. Since it appears that the scripts relevant for the OMICS data processing could not be found easily, we cleaned up the GitHub repository for the revised submission of the manuscript and explained with more detail in the ReadMe file where individual scripts can be found.

11.
Data availability: The raw data from the transcriptome and protein analysis should be made available via common databases upon publication.

See above.

12.
Supplemental tables for all OMICS data (lncRNA, cirRNA, piRNAs, proteins, etc.) should be provided as the raw data is currently inaccessible.

We can include Supplementary tables if the editor wishes also in the manuscript. For now, they are all available via the web application and in the GEO repository.

13.
Fig 2A: The heatmap depicts 339 microRNAs, yet the text mentions both 339 and 181. Please clarify.

We apologize for the confusion, as described in the main text, the 339 miRNAs correspond to microRNAs significantly changing over the entire time course while there where 181 miRNAs significantly changing when only considering the neuronal time points (Day 9 – Day 40). In total we detected 455 as being robustly expressed in our sequencing dataset.

14.
Description of the data shown in Fig 2 and Fig S11 needs improvement in the main text. It's challenging to comprehend how the mouse data was generated and compared to ig Neuron data.

As described in the methods section, this data was generated by Whipple et al., Mol. Cell, 2020. For a detailed description of how the original data was generated we refer to their methods section. With regards to how we processed the data we would like to refer to our methods section and to the GitHub repository where the script to generate the panels initially found in Fig. 2 and Fig. S11 can be found under
01_TimeCourse_MultiOmics/Figure_Omics/SmallRNA_Mouse/Whipple_2020_SmallRNA_Figures.Rmd. The script for the differential expression analysis can be found in 01_TimeCourse_MultiOmics/smallRNA/smallRNA_R.

15.
The authors provide RNAscope images for miR-1229-3p. It would be interesting to describe if this microRNA has a synaptic localization synaptic mitochondria. Although the authors later focus on PINK1 it would be important to test if miR-1229-3p could target synaptic mRNAs. This could be done via bioinformatic approaches, since a number of suitable datasets on the synaptic RNAome and Proteome exist.

Regarding the miR-1229-3p in situ images, we want to stress that detection of this relatively lowly expressed microRNA was extremely challenging. Due to the stringent protocol required to obtain a specific signal, we can only detect a few miR-1229-3p positive puncta per cell, and only a very small fraction of those is localizing to processes. Therefore, making any claims regarding synaptic localization of this miRNA based on a co-localization of miR-1229-3p FISH puncta with a synaptic protein is impossible. This would require an extensive optimization of the current protocols for single molecule miRNA FISH which is clearly beyond the scope of this manuscript.

Regarding potential synaptic targets of miR-1229-3p, we indeed obtained evidence from our new experiments in SH-Sy5y cells, which favors the identification of direct mir-1229-3p targets. Indeed, synapse-associated GO-terms are among the most enriched in differentially expressed genes in this dataset, especially when focusing on transcripts containing miR-1229-3p binding sites (Fig. 6e). Examples of such putative synaptic targets are provided in Fig. 6h and further discussed.

16.
Fig 4a. please provide the raw data and the list of diff. expressed genes as a supplementary table.

See above (we can include a specific supplementary table for the manuscript).

17.
Fig 17d. What are the genes up-regulated upon miR-1229-3p inhibition in igNeurons?

See above (the DEA table is currently available on GEO, however we can include it in the manuscript if required).

18.
Fig. 4. How many of the genes regulated by miR-1229-3p are changing during igNeuron development (Fig 1)?

To determine how many genes potentially regulated by miR-1229-3p are changing during neuronal development, we filtered for genes significantly downregulated in the SH-Sy5y sequencing (FDR < 0.05) that contain 7mer or 8mer sites in their UTR (as for Fig. 6e)). We obtained 100, which we subsequently compared to our time course expression data. 33 of these candidate target genes change during the time course of igNeuron differentiation (FDR < 0.01 & logFC > 2) and 6 of them during the neuronal time points (day 9 – day 40, FDR < 0.01 & logFC > 2). These 6 genes are: "IGFBPL1", "E2F1", "SLC6A2", "P2RY6", "TRIM56", & "CDK2".

19.
While the fact that miR-1229-3p seems to affect all mt-genes is interesting, I am not sure that its fair to say that "many" of the TOP15 GO terms were associated with mitochondrial function. Also please provide the entire list of significant GO terms as a supplementary table for all datasets.

We thank the reviewer for the clarification and replaced many with several. The entire GO-Term results tables are already in the GitHub repository. For the pLNA sequencing these can be found in 03_pLNA_GRCh38.p13_Ens107/pLNA_experiment/longRNA_R/GO_Terms/GOdata and for the SH-Sy5y Sequencing in the corresponding longRNA_R and Target_Analysis folders. Since we performed individual GO-Term analysis for DEGs as well as up- and downregulated genes on all three ontologies for the pLNA-sequencing and SH-Sy5y sequencing datasets as well as an analysis of the potential targets of the SH-Sy5y dataset (not all are individually displayed), these would comprise in total 21 tables which we can of course include if required. We would like to further clarify that, for consistency, we changed all GO-Term analyses in the revised manuscript to using gene symbol identifiers.

20.
Does miR-1229-3p affect PINK1 protein levels?

Since we couldn't validate Pink1 regulation in the SH-Sy5y miR1229 overexpression experiment, and Pink1 TSB treatment did not recapitulate the synaptogenesis phenotype (see comment before), we decided not to provide additional data regarding the potential regulation of Pink1 expression by miR-1229-3p in the revised version of the manuscript.

21.
The authors mention a SNP within the miR-1229-3p gene that has been associated with Alzheimer's disease. Does this SNP lead to loss of miR-1229-3p function and would it affect PINK1 levels? A simple approach would be to repeat the experiments shown in Fig. 3F if the SNP lies within the mature sequence of miR-1229-3p.

The reported SNP in the miR-1229-3p gene was reported to enhance production of mature miR-1229 (Sci. Rep. paper) and hence should cause a stronger downregulation of a perfect binding site reporter

compared to the wild-type. To validate these previous findings, we tested the activity of the wild-type and mutant miR-1229-3p (rs2291418) with a luciferase reporter gene assay (see below). However, in this assay, we didn't observe a clear difference in their ability to downregulate the reporter gene. Therefore, a more detailed characterization of the functional relevance of this SNP will have to be done in future studies, which is beyond the scope of the present manuscript.

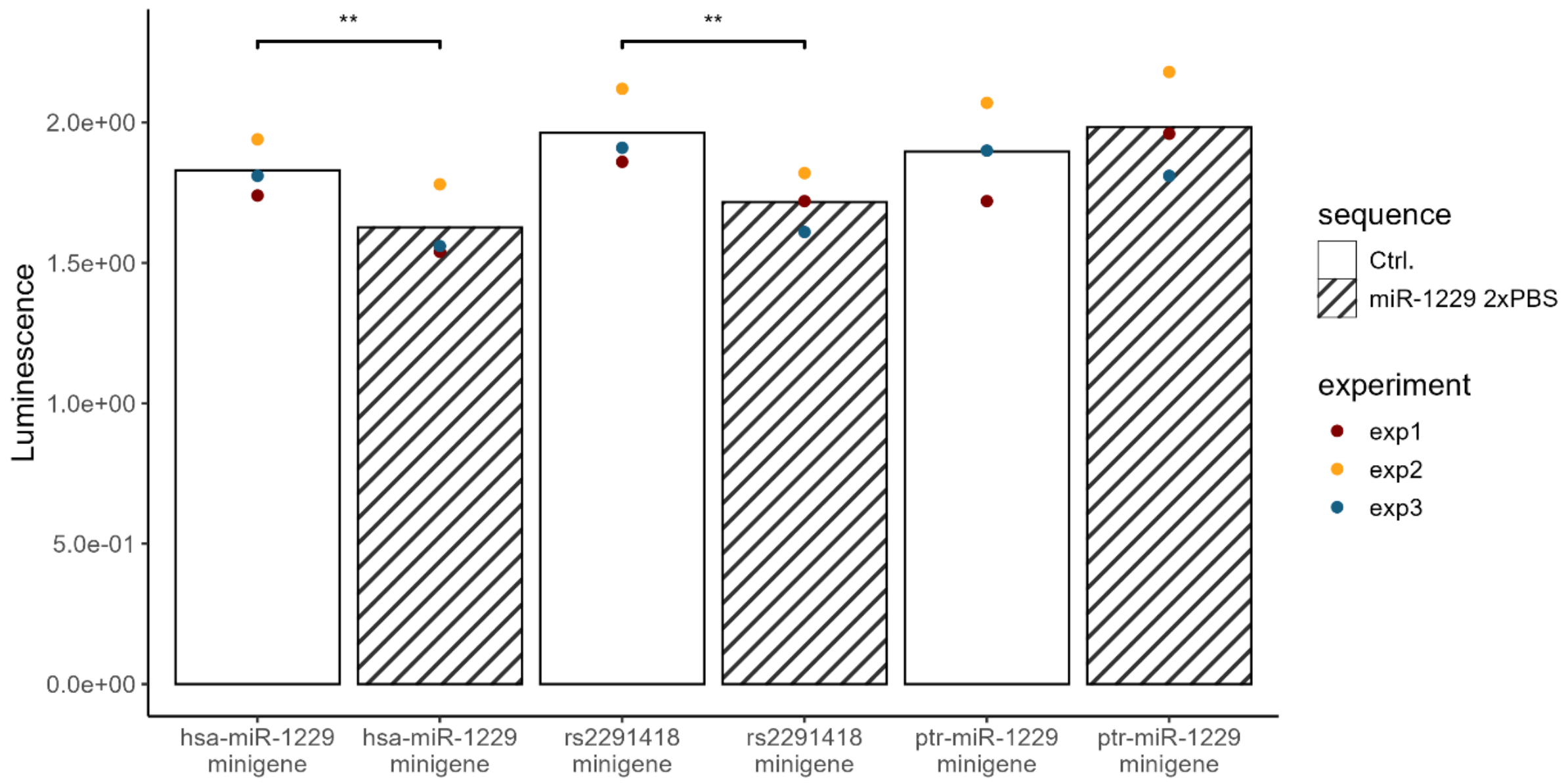

*Luciferase measurements of different versions of a hsa-miR-1229 minigene together with a PBS-reporter or the respective control. A linear model with an interaction effect for the sequence and minigene version and a fixed effect for the replicate was used for the statistical analysis (~ condition*sequence + experiment). Statistical comparisons were acquired using emmeans by comparing the effects on the sequence seperately per condition (sequence|condition, reference = Ctrl.).*

22.
It is interesting to see that miR-1229-3p also affects rat PINK1 when transfected into rat cortical neurons.

We would like to clarify that we transfected the human Pink1 3'UTR into rat cortical neurons and also specifically clarified that in the figure legend of the revised manuscript.

At the same time, there are several confirmed and predicted rodent microRNAs that can target PINK1.

Are any of these expressed in the igNeurons? If so, how is their expression pattern during differentiation?

Would the expression of a murine-specific microRNA that targets PINK1 in igNEurons affect mitochondria function and synapse development.

What is the expression pattern of Pink1 in igNeuron and rat neuronal development?

As alluded to in our previous comments, we have de-emphasized the role of Pink1 downstream of miR-1229-3p, mainly due to our new results from RNA-seq upon miR-1229-3p overexpression (new

Fig. 6) and Pink1 TSB (see above). We therefore felt that identifying additional microRNAs targeting Pink1 was not a high priority for the manuscript revision. For the expression patterns of individual genes during igNeuron development, we would like to refer to our web app at https://ethz-ins.org/igNeuronsTimeCourse

20.
As mentioned above it is sometimes very laboursome to follow the text, the figures and the legends. Referring for example to Fig 1C, it's unclear what SC1, 2, and 3 stand for. While I assume they represent the three differentiations, 'sc' might also denote synapse clusters. Such ambiguities occur repeatedly throughout the manuscript and should be addressed for better understanding before publication.

We have now carefully addressed all these issues in the revised manuscript.

The review process was also complicated since there are no page/line numbers.

We apologize and have now included page and line numbers in the revised manuscript.

Reviewer #1 (Remarks to the Author)

Soutschek et al. have included new data in this revised manuscript that address the points that I have raised. Interestingly, these new data suggest that miR-1229-3p inhibition leads to lower mitochondrial activity and mitochondrial dysfunction, although not to the extent that mitophagy is increased, ultimately leading to altered Ca2+ regulation and dendritic arborisation. Despite this mitochondrial dysfunction, miR-1229-3p inhibition enhances synaptogenesis, and so this contrasts with Iwata et al. (ref. 32 in their manuscript) where it is reported that decreasing mitochondrial metabolism slows neuronal development. How can the authors' findings be reconciled with Iwata et al.? Mito-ER interactions are considered in the discussion and indeed they mention that MERC regulation by miR-1229-3p could be a mechanism in human neurons contributing to slowing of mitochondrial metabolism and associated neuronal maturation. However, their findings don't appear to support the premise that impaired mitochondrial metabolism slows neuronal maturation. I would be interested in additional discussion on this point.

We are happy that this reviewer states that we have addressed the points they originally raised. Moreover, we are convinced that our findings can be easily reconciled with the data presented in Iwata et al. (Ref. 32). At early time points of miR-1229-3p inhibition, the upregulation of genes involved in Mito-ER interactions and the likely induction of an ER stress response is expected to initially promote mitochondrial metabolism (Carreras-Sureda et al., *Sci Signal* **15**, eabm7524 (2022)), which in turn is consistent with the observed accelerated synaptogenesis. In contrast, prolonged inhibition of miR-1229-3p leads to mitochondrial dysfunction, including reduced mitochondrial metabolism, which aligns with the observed reduced dendritic arborization at later time points of neuronal development. We have now attempted to more clearly explain this time-dependent regulation of mitochondrial metabolism by miR-1229-3p in the discussion of the revised manuscript (p. 25-26).

We further would like to indicate that we had to remove our data from mitochondrial calcium imaging experiments (Fig. 5i-k of the revised manuscript) due to technical issues with the filter settings of the microscope. After submission of the revised paper, we realized that the filter wheels were exchanged without proper communication, during imaging of the calcium dye Rhod2, which yielded a high degree of non-specific signal which might obscure the actual specific calcium signal. We were therefore unable to assign the observed differences in fluorescence intensity between the pLNA-control and -miR-1229-3p condition unequivocally to the Rhod2 signal and decided to dismiss these measurements. We want to stress that miR-1229-3p-dependent control of mitochondrial function is still supported by results from several orthogonal experiments, e.g.,

mitochondrial DNA (Fig. 5a-c), mitophagy (Fig. 5d), morphology (Fig. 5e-f) and metabolism (Fig. 5g-h).
